# Supplementary material for: Secondary acceleration of slip fronts driven by slow slip event coalescence in subduction zones
Source: Nat Commun. 2025 Oct 29;16:9561. doi: 10.1038/s41467-025-64616-3 (PMC12572340; doi:10.1038/s41467-025-64616-3)
Supplement: Supplementary file 1 — Supplementary Information [file 41467_2025_64616_MOESM1_ESM.pdf]

Supporting Information

for

Secondary acceleration of slip front driven by slow slip event coalescence in subduction zones

Ji Wang[1,2], Kejie Chen[1,3*], Sylvain Michel[4,5,6], Luca Dal Zilio[7,8], Hai Zhu[1], Lei Xia[1], Jun Xie[1], Shunqiang Hu[9]

[1]Department of Earth and Space Sciences, Southern University of Science and Technology, Shenzhen, China

[2]Shanghai Innovation Institute, Shanghai, China

[3]Key Laboratory of Earthquake Forecasting and Risk Assessment, Ministry of Emergency Management, Southern University of Science and Technology, Shenzhen, 518055, China.

[4]Université Côte d'Azur, IRD, CNRS, Observatoire de la Côte d'Azur, Géoazur, Valbonne, France

[5]CNRS-INSU, Institut des Sciences de la Terre Paris, Sorbonne Université, ISTeP UMR 7193, 75005 Paris, France

[6]Laboratoire de Géologie, Département de Géosciences, Ecole Normale Supérieure, PSL Université, Paris, France

[7]Earth Observatory of Singapore, Nanyang Technological University, Singapore, Singapore

[8]Asian School of the Environment, Nanyang Technological University, Singapore, Singapore

[9]Key Laboratory of Poyang Lake Wetland and Watershed Research, Ministry of Education, Jiangxi Normal University, Nanchang, China

* Corresponding author: K. Chen, chenkj@sustech.edu.cn

Contents of this file

Supplementary Text S1 to S5

Supplementary Table 1

Supplementary Figures S1 to S65

Supplementary Figures Overview:

1. Figures S1. Conceptual diagram of SSEs coalescence.
2. Figures S2-S3. Station distribution and fault geometry of the Cascadia subduction zone.
3. Figures S4. vbICA preprocessing of GNSS time series.
4. Figures S5-S6. Deep learning detection results of SSEs.
5. Figures S7-S16. Supplementary files for SSE 44.
6. Figures S17-S23. Supplementary files for numerical simulations.
7. Figures S24-S25. Supplementary files for scaling law.
8. Figures S26-S68. Supplementary files for the inversion results of all SSEs.

Text S1. Time Series Preprocessing

In our original study [1], we use a signal decomposition technique based on the variational Bayesian Independent Component Analysis (vbICA) to distinguish the signals related to slow slip events (SSEs) from the signals associated with tectonic and non-tectonic processes in the GNSS detrended position time series [2] [3]. The specific process is as follows: First we select GNSS time series at 240 stations (see distribution in Fig. S2) in the CSZ, the observed epochs are T = 4108 days (11 years with daily sample from 2012 to 2022). This dataset is archived by SOPAC at ftp://garner.ucsd.edu/pub/timeseries/measures/ats/WesternNorthAmerica, corresponding to the "WNAM Clean Detrend" product. These time series have been corrected to eliminate a long-term linear trend and offset variations, encompassing both instrumental and tectonic influences. We employ the Interquartile Range method to remove outlier values and the regularized expectation–maximization algorithm to fill the missing values[4] [5]. Then we use vbICA three times to select the components interpreted as SSEs. In the first round of vbICA, we divide the Cascadia subduction zone (CSZ) into northern and southern regions along the 45°N for separate analysis. For the northern region, we set the number of extracted components to be 7 (account for over 70.17% variation of data), we do not remove any components because there are no clear elements associated with the earthquakes. For the southern region, we set the number of extracted components to be 8 (account for over 70.90% variation of data) and removed seismic signal components that were not corrected in the dataset. In the second round of vbICA, we set the number of extracted components to be 11 (account for over 72.44% variation of data) and removed the first 7 components that associate with seasonal signal, common mode errors and local efects. In the third round of vbICA, we set the number of extracted components to be 23 (the Negative Free Energy is -4.79×10^6) and remove the noise components to ensure the Signal-to-Noise Ratio is maximized. All the removed components are shown in Fig. S3. The processed data serves as input for the deep learning detection model and the finite fault inversion model.

Text S2. Deep learning model

We use the deep learning model [1] to detect SSEs in GNSS time series of 240 stations. The input of the model is the GNSS time series, and the output is the occurrence time and duration of the SSEs. The model can simultaneously input three-component GNSS time series for detection. We normalized the three-component data of 240 stations before inputting it into the model. The model's output represents the probability, ranging from 0 to 1, that each station is classified as experiencing an SSE at each time point. We set the detection threshold for SSEs at 0.98, meaning that any time point with an output probability greater than 0.98 is considered to indicate the presence of an SSE. This high threshold helps to minimize false positives, ensuring that only the most confident detections are identified as actual SSE occurrences.

Although we performed preprocessing of the GNSS time series using vbICA, noise still persists in the data from each station. To minimize the likelihood of detection errors (false positives) due to noise, we require that at least four stations must detect an SSE for us to consider that an SSE has actually occurred. This collective detection approach helps to filter out random noise and enhances the robustness of our SSE identification process by relying on corroboration from multiple s
tations before confirming an SSE event. The detection results are displayed in Fig. S5 and S6.

Text S3. Dynamic resolution tests

We assess the accuracy of the dynamic inversion to model deformation of SSEs 50&51 in the southern part of the CSZ in March 2021. We construct a synthetic model slip composed of two patches, roughly corresponding to the two rupture fronts in the cumulative slip map (see Fig. S11).

We simulate the time evolution of slip in each sub-fault using the following exponential function:

$$d(t) = d_{cum}/(1 + exp(-(t - t_{max})/(t_{dur}/\sigma)))$$

where $d(t)$ is the slip amplitude of all dates, d_{cum} is the cumulative slip, t_{max} is the time when the slip rate is at its maximum, t_{dur} is the duration of slow slip, and σ is the parameter controlling the slip rate.

We introduce white noise to the horizontal components in the time series data derived from forward modeling simulations. We perform inversion with the regularization $\lambda_{space} = 4$ and λ_{time}=1. The kinematic inversion can retrieve with good accuracy the synthetic slow slip distribution in space and time (see Fig. S11). The simulated moment is Mw 6.4, and the inversion result is Mw 6.5, which is 0.1 larger than the simulated value. The source time functions show that the date of the maximum slip rate in the main slip zones is accurately reflected. The maximum cumulative slip is lower after the inversion, 9mm in the southern part compared to 8.9mm, and 8.5mm in the northern part compared to 6.8mm.

Text S4. The rate-and-state model set up

We consider a megathrust fault segment embedded into an elastic medium, loaded by deep-seated slip at the long-term slip rate, and governed by an updated rate-and-state friction law with a transition from VW to VS at steady-state with increasing slip rate. When the cut-off velocity to the evolution effect is significantly smaller than that of a direct effect, steady state friction behaves as velocity weakening at low slip velocity and velocity strengthening at high slip velocity. We set the fault extends 330 km along the strike direction and 60 km depth (see Fig. 3a). The depth of the VW area ranges from 20 to 40 km. The edge of the VW area is designed with a curved arc using the following function:

$$a = 2 * b * (1 - 0.9e^{\frac{(x-x_{center})^2+(z-z_{center})^2}{2*scale^2}})$$

To simulate the elongated heterogeneous coupling features of the coupling map. We set the value to -0.0035 for x>75 km in the VW area. In the region where x<75 km, the $a - b$ value gradually increases. The model setup is shown in Fig. 3a, and the relevant model parameters are listed in Supplementary Table 1.

Text S5. Fit of Data to the Gutenberg-Richter Frequency-Magnitude Distribution

To fit SSEs data to the Gutenberg-Richter Frequency-Magnitude Distribution (GR-FMD), we use the discretized maximum likelihood method [6]. The minimum magnitude of complete recording (M_{c1}) is an important parameter that can significantly impact the resultant GR-FMD. In order to consider the effect of the inflection point (M_{c2}) for the GR law, we perform maximum likelihood fits to the GR-FMD dataset based on a range of M_{c1} and M_{c2}. To estimate the goodness of the fit, we compute the square difference, R^2, of the number of events in each magnitude bin between the observed and synthetic distribution:

$$R^2(a, b, M_i, M_j) = 1 - \left(\frac{\sum_{M_i}^{M_{1max}}(B_i - S_i)^2}{\sum_i B_i} + \frac{\sum_{M_j}^{M_{2max}}(B_j - S_j)^2}{\sum_j B_j}\right)$$

where B and S are the observed and predicted cumulative number of events in each magnitude bin. We set the magnitude bin width as 0.05. The goodness of fit R^2 as shown in Fig. S17. The M_{c1} and M_{c2} selected are the magnitudes at which 90% of the observed data are modeled by the power law fit. Ultimately, we obtained M_{c1}=5.6 and M_{c2}=6.3.

Supplementary Table 1. Frictional parameters used in the rate-and-state model

**parameter**	**symbol**	**value**
fault length	L	330km
fault depth	Z	10-50km
fault dip	φ	20°
shear modulus	G	30GPa
reference friction coefficient	μ^*	0.6
velocity plate	V_{pl}	40mm/yr
effective normal stress	σ_n	1MPa
characteristic slip distance	D_c	0.7mm
a value in VW region	a	0.0065
b value in VW region	b	0.01
a value in VS region	a	0.013
b value in VS region	b	0.01
length of VW region	L_{vw}	300km
depth of VW region	Z_{vw}	20-40km
cut-off velocity of direct effect	V_1	0.01m/s
cut-off velocity of evolution effect	V_2	1e-7m/s

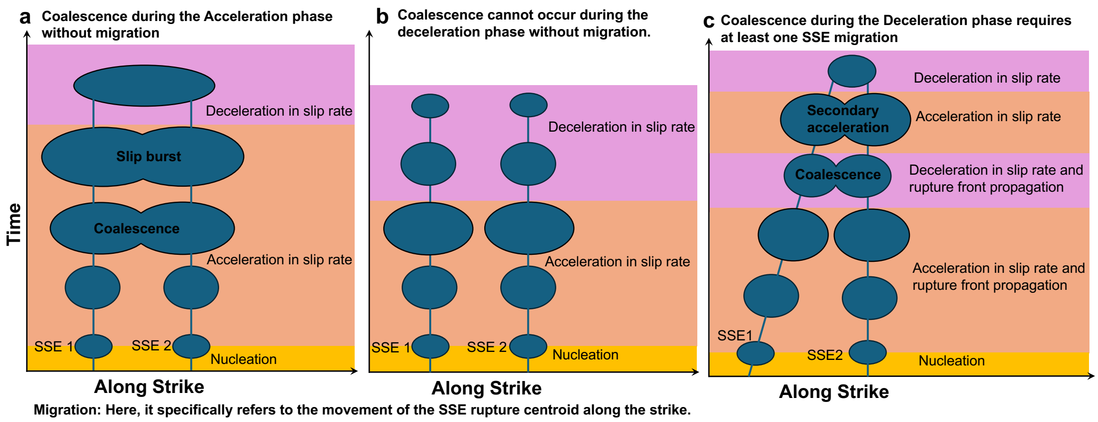

Figure S1: Schematic diagram of the movement of the slow slip event rupture centroid along strike. (a) The coalescence process during the acceleration phase without migration. (b) The coalescence cannot occur during the deceleration phase without migration. (c) The coalescence during the deceleration phase requires at least one SSE migration. The yellow area represents the nucleation phase, while the orange and pink areas indicate the acceleration and deceleration phases, respectively.

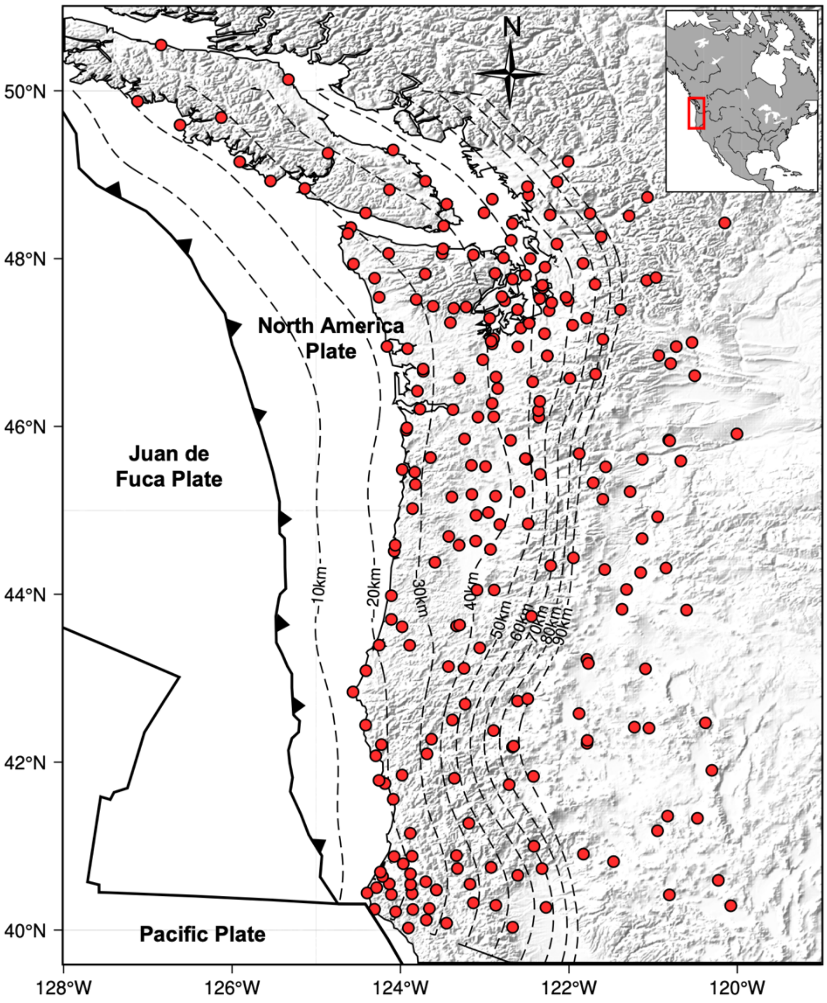

Figure S2: The CSZ geographical location and GNSS station distribution. The GNSS stations are denoted by red circles, with a total of 240 stations. The black solid lines are the plate boundaries, and the black dashed lines are the depth contours every 10 km provided by Slab2.0 [7].

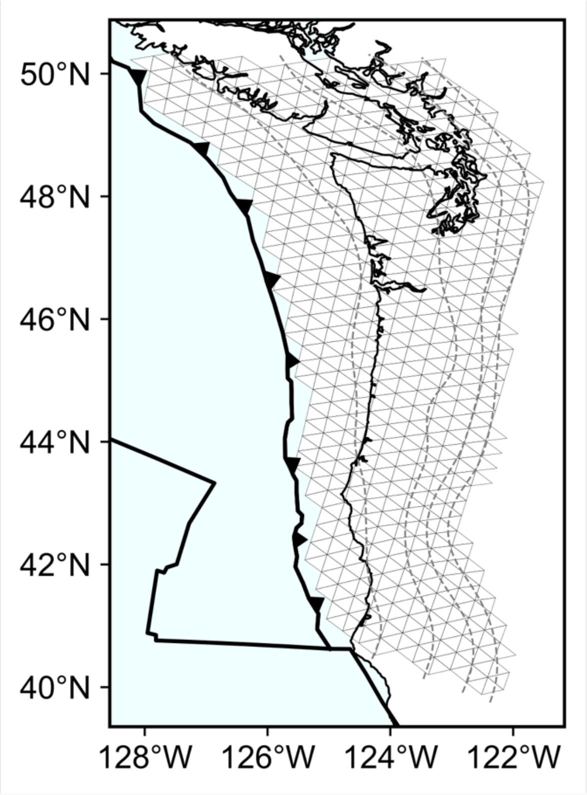

Figure S3: Fault geometry used for the kinematic inversion. The CSZ fault is discretized into 802 quasi-equilateral triangular sub-faults with 19.6 km long edges, following the curved surface of the Slab2.0 model [7]. The fault area extends from 39°N to 51°N and from the trench down to 90 km depth. The dashed lines are the Slab2.0 iso-depth contours every 20 km [7].

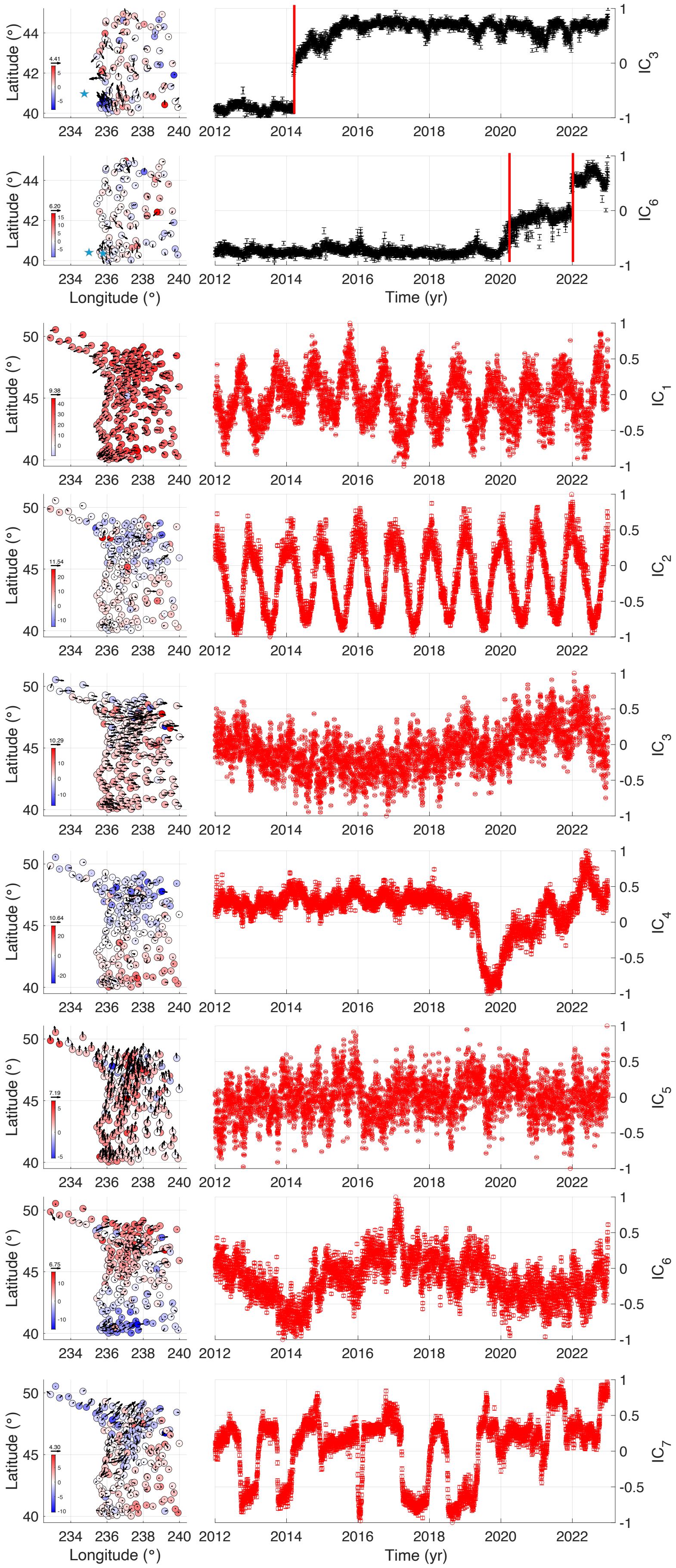

Latitude (°)
Longitude (°)
Time (yr)
IC$_3$
IC$_6$
IC$_1$
IC$_2$
IC$_3$
IC$_4$
IC$_5$
IC$_6$
IC$_7$

Figure S4: Components removed in the first and second round of vbICA. The left column panels display the spatial patterns of the component. Arrows represent horizontal motion, while colored dots represent vertical motion. The right column panels illustrate the temporal evolution of the components. For the first round of vbICA, The yellow stars indicate the spatial locations of 3 earthquakes at their epicenters occurring near the triple junction point in left column panels. Red lines indicate the time of the earthquakes in the right column panels. The three earthquakes are as follows: March 10, 2014 - Magnitude 6.8, March 9, 2020 - Magnitude 5.8, December 21, 2021 - Magnitude 6.2. For the second round of vbICA, IC1 and IC2 show seasonal signals, IC3 and IC5 related to common mode error, IC4 exhib the north–south diference in the first round of vbICA, IC6 and IC7 is associated with the local effects.

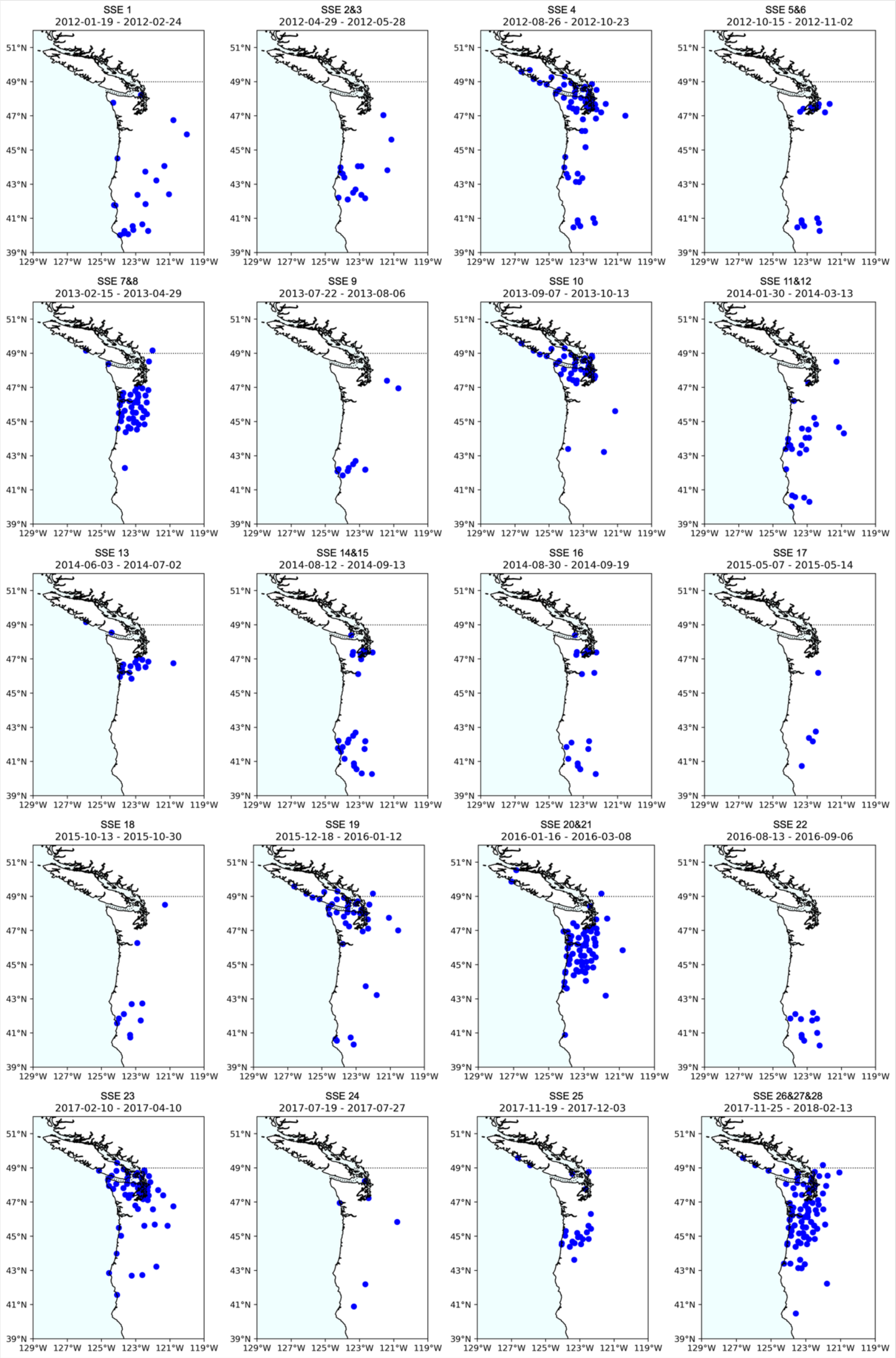

Figure S5: SSEs detection results of the deep learning model for SSEs 1-28. The blue dots represent stations where slow earthquakes were detected during the duration of the SSES.

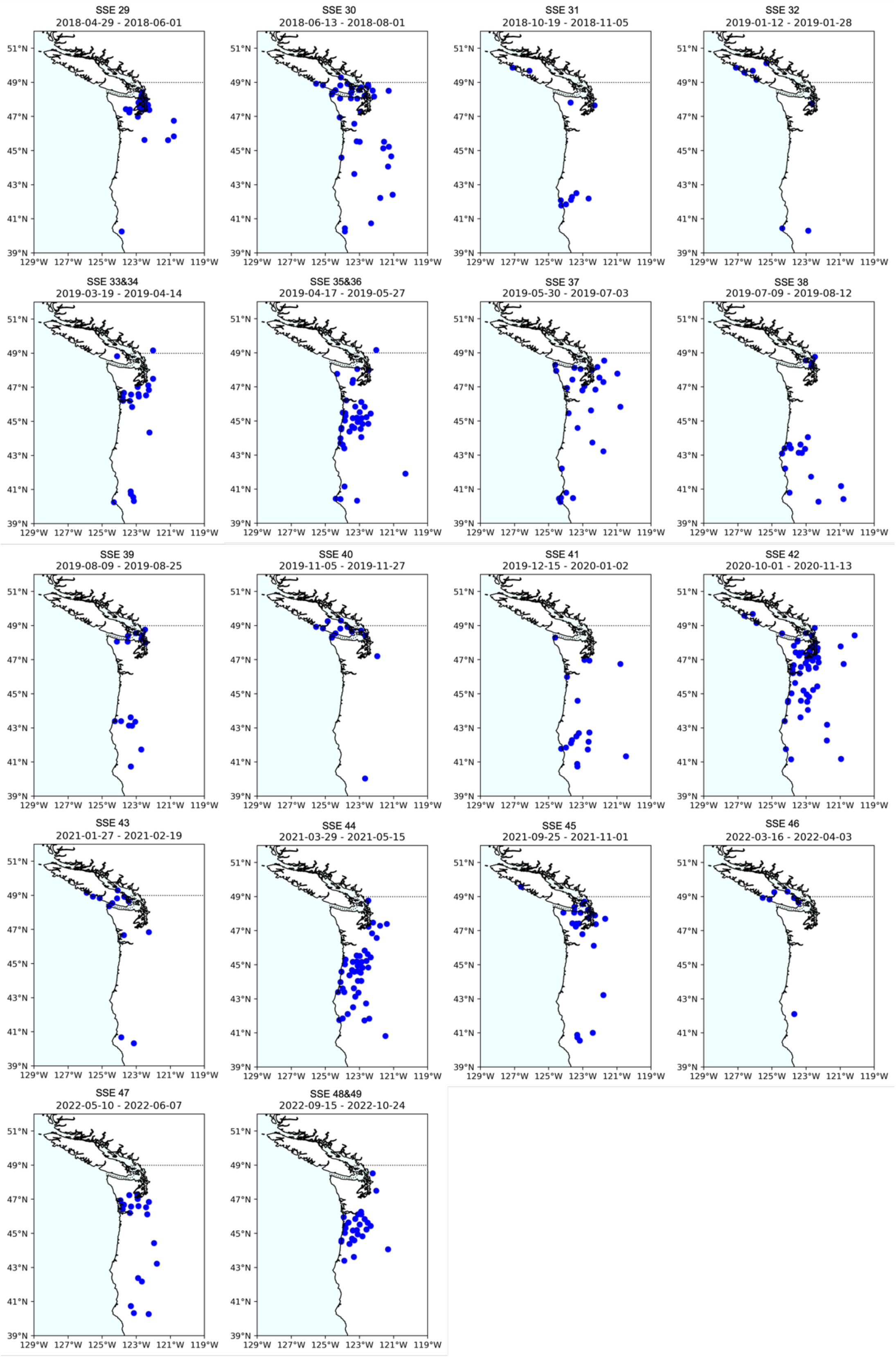

Figure S6: SSEs detection results of the deep learning model for SSEs 29-49. The blue dots represent stations where slow earthquakes were detected during the duration of the SSEs.

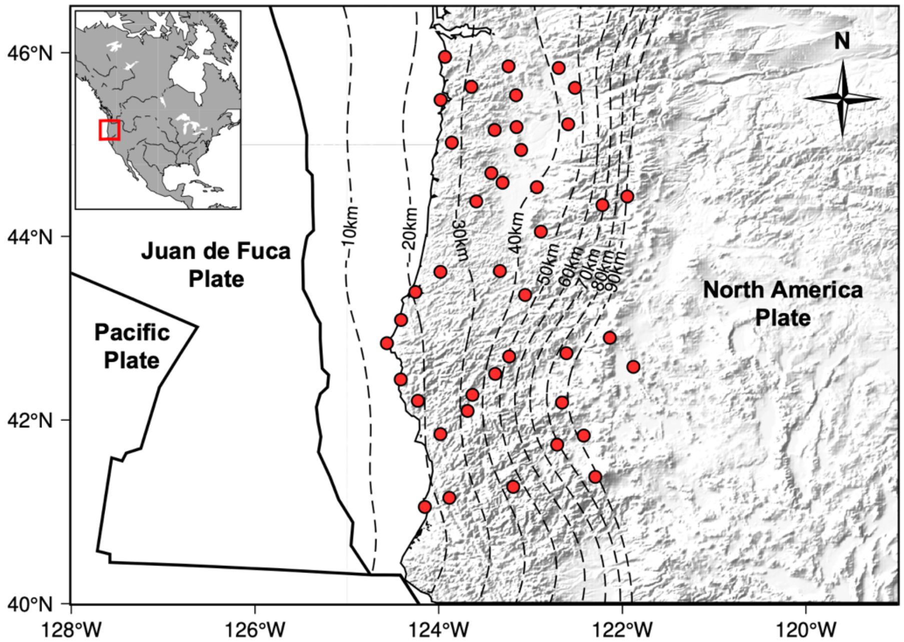

Figure S7: GNSS network used in SSE 44. The circles mark the locations of the GNSS sites used for kinematic inversion. The dashed lines are the Slab2.0 iso-depth contours every 20 km [7].

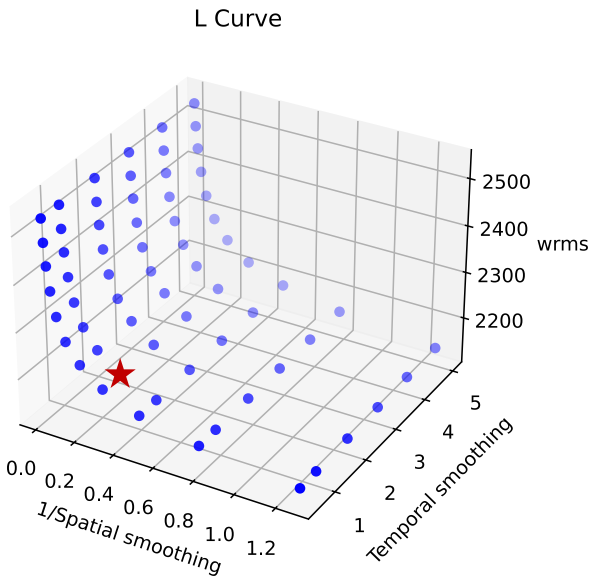

Figure S8: L-curve for inversion in SSE 44. Weighted Root Mean Squared (WRMS) is a function of the model roughness which is defined as the reciprocal of the spatial smoothing factor and the temporal smoothing factor. The red star indicates the selected value for the optimal inversion, corresponding to λ_{space} = 4 and λ_{time} = 1.

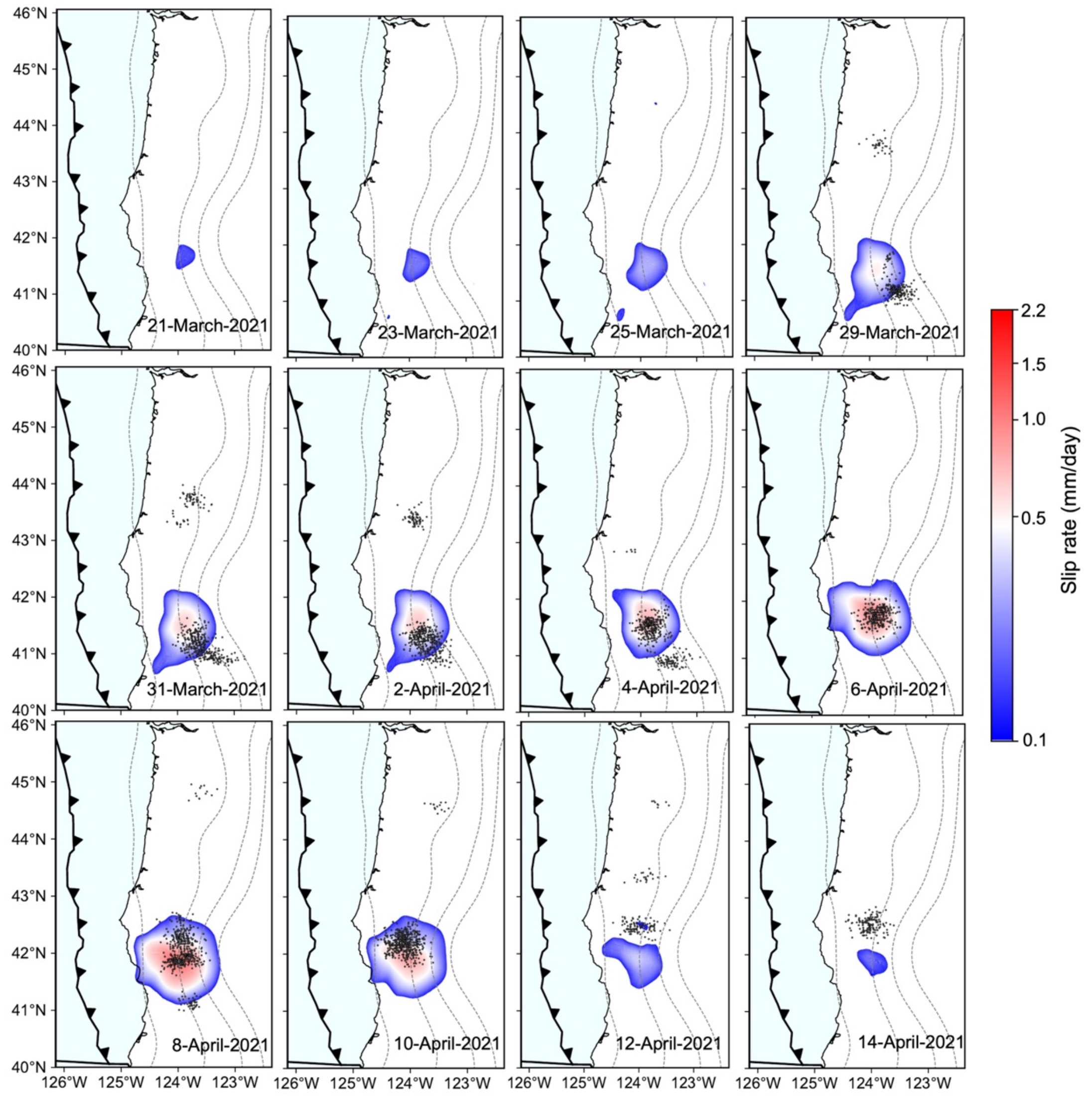

Figure S9: The evolution of ETS in the southern part of Cascadia from March 21 to April 14, 2021. Daily slip rate contoured in color maps. The black dots represent the corresponding daily tremor locations from the Pacific Northwest Seismic Network (PNSN) catalog. The dashed lines indicate the Slab2.0 depths every 20 km[7].

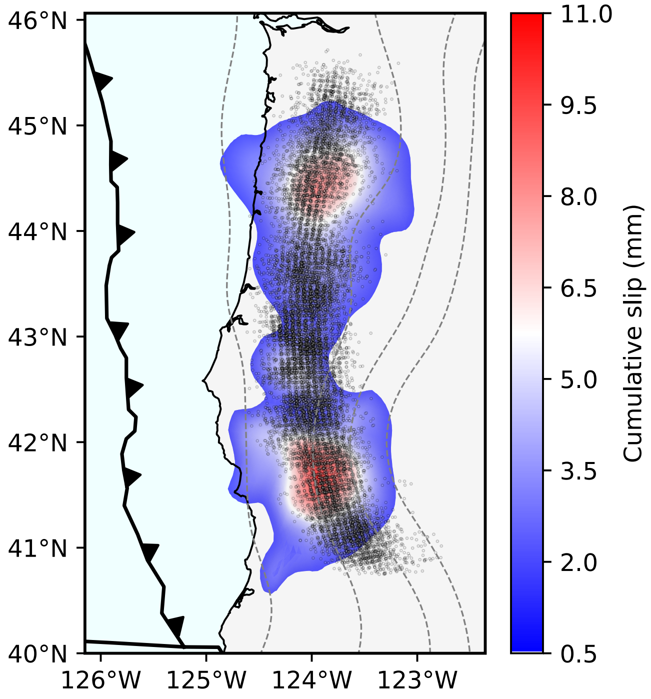

Figure S10: The cumulative slip distribution from March 27 to May 10, 2021. Daily tremors recorded by the Pacific Northwest Seismic Network are depicted as gray dots. The dashed lines represent the Slab2.0 depths at 20 km intervals.

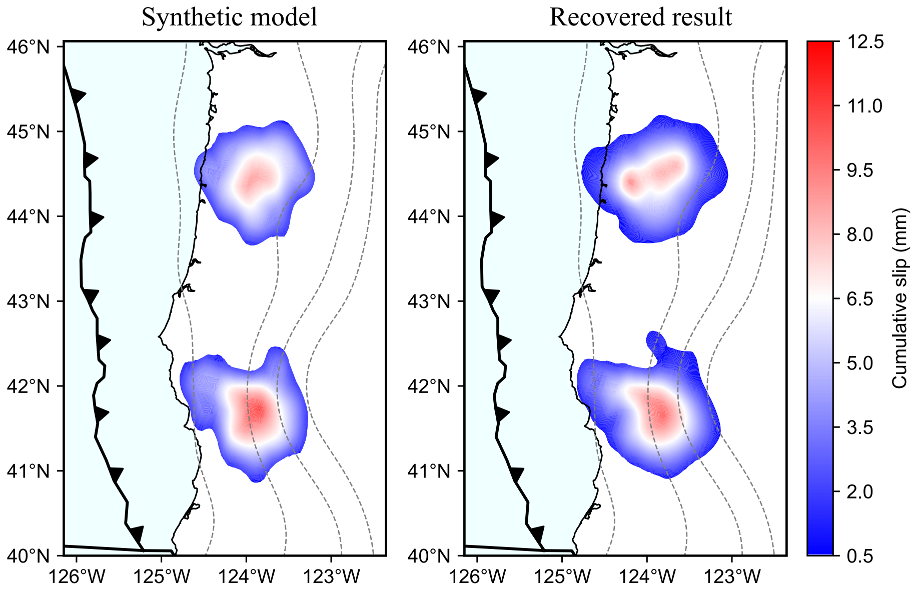

Figure S1: The cumulative slip distribution of dynamic resolution test for SSE 44. With the synthetic model (left) as input, we get the recovered result (right). The dashed lines represent the Slab2.0 depths at 20 km intervals.

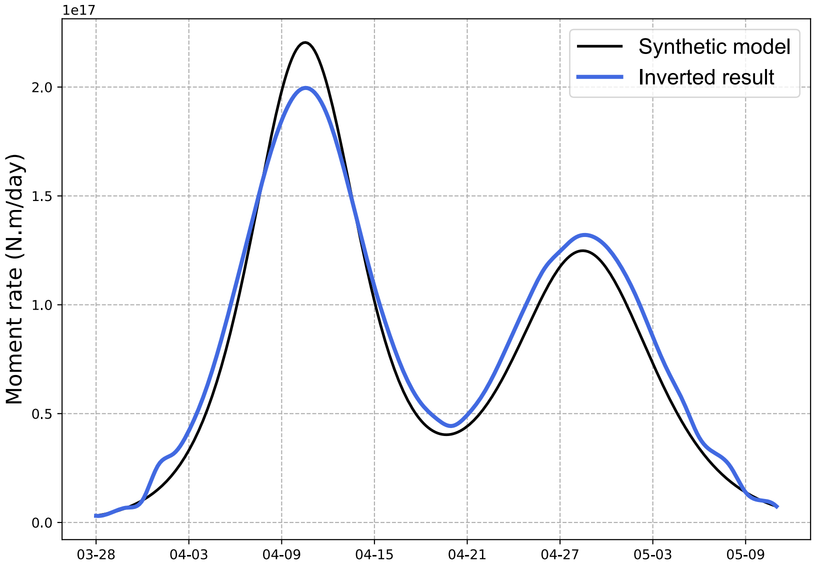

Figure S2: The moment rate of the dynamic resolution test. The black and blue solid lines show the synthetic and the inverted moment rate values, respectively.

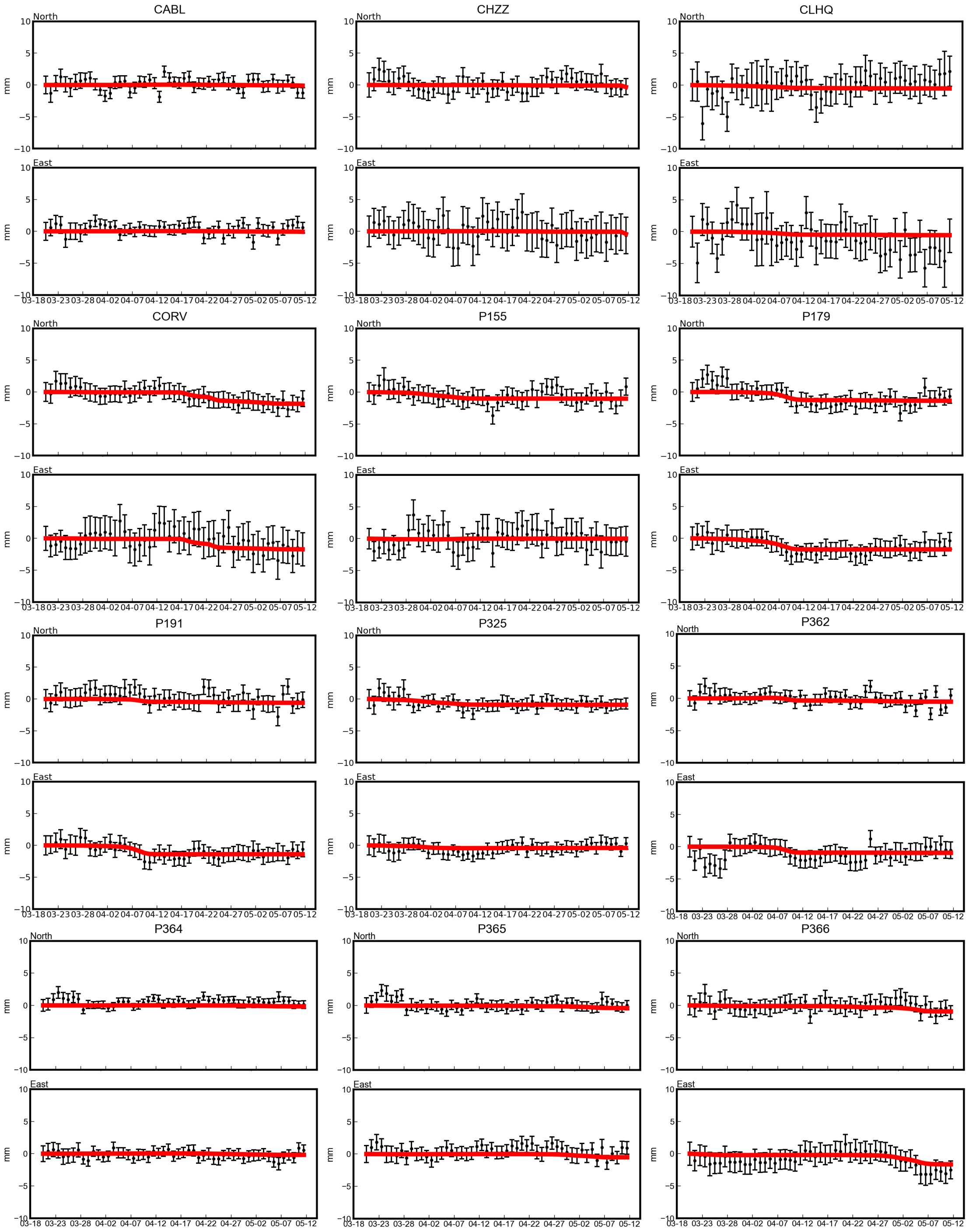

Figure S3. Data fit for stations CABL, CHZZ, CLHQ, CORV, P155, P179, P191, P325, P362, P364, P365, P366.

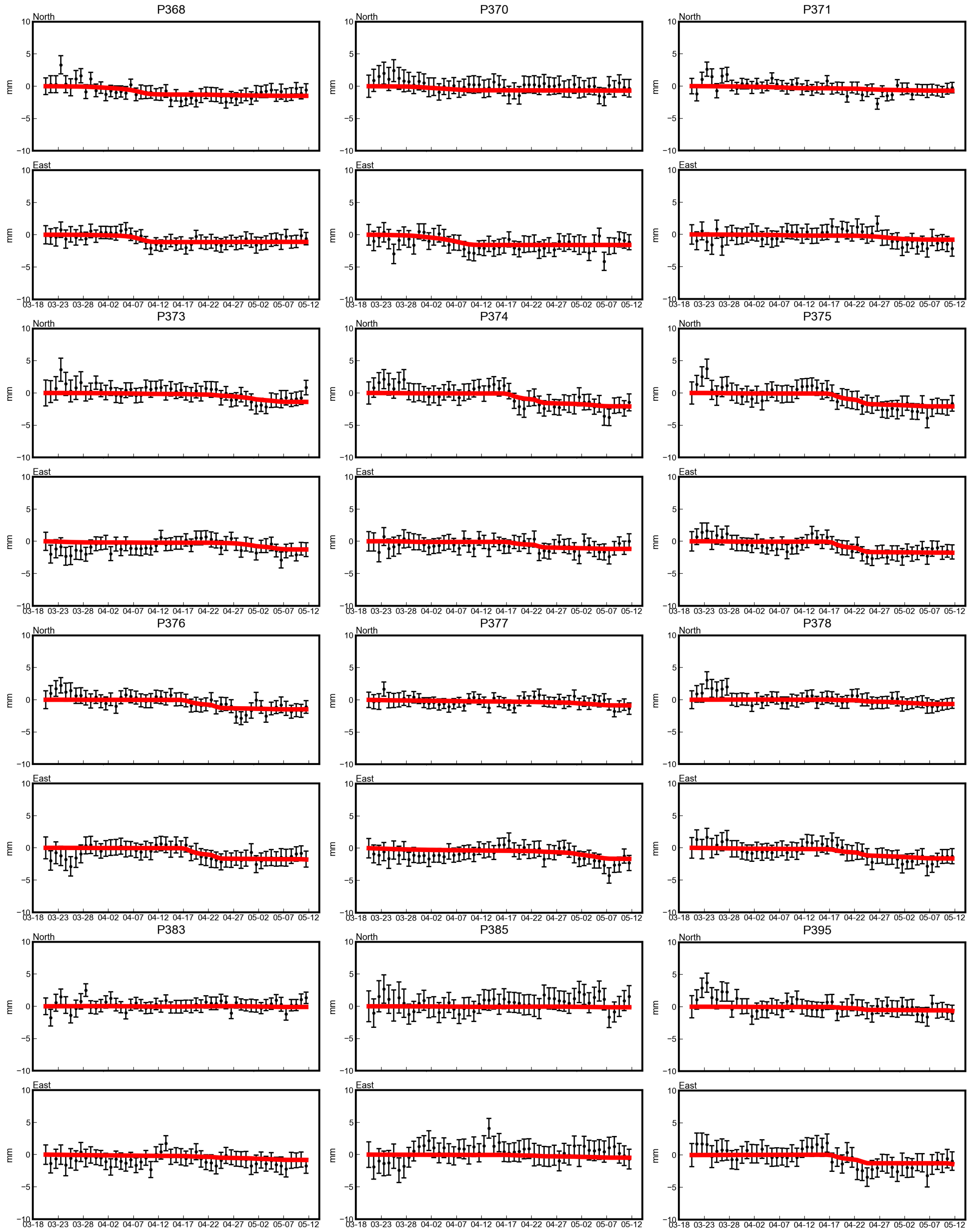

Figure S14. Data fit for stations P368, P370, P371, P373, P374, P375, P376, P377, P378, P383, P385, P395.

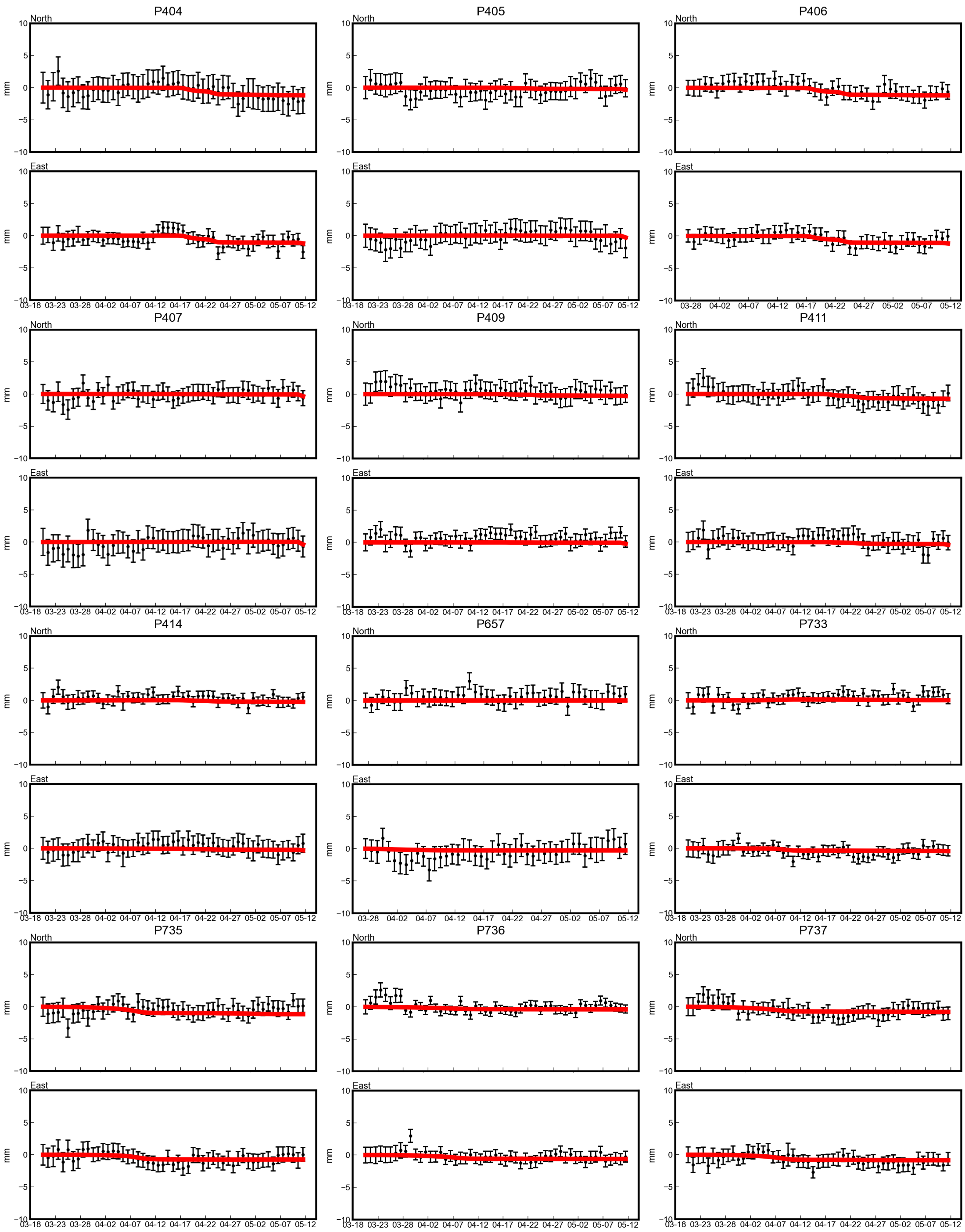

Figure S4. Data fit for stations P404, P405, P406, P407, P409, P411, P414, P657, P733, P735, P736, P737.

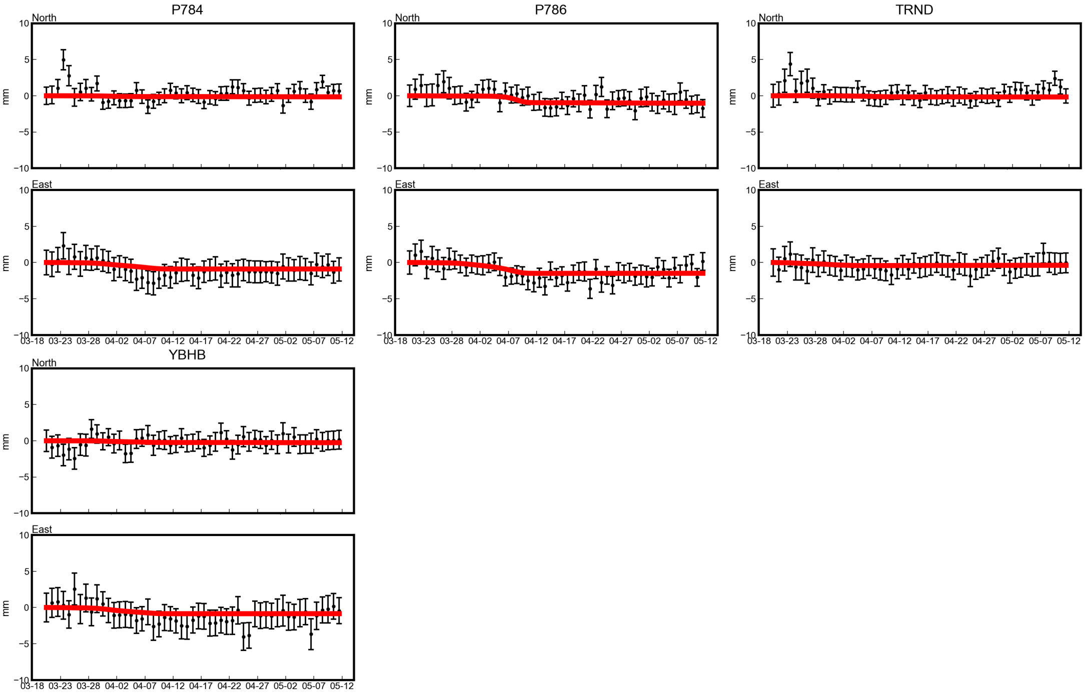

Figure S5. Data fitting for stations P784, P786, TRND, YBHB.

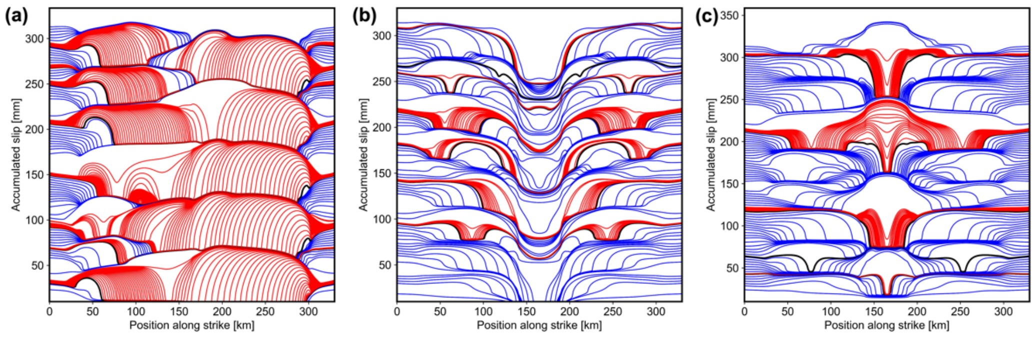

Figure S17. **Accumulated slip of three models for 10 years simulations** Accumulated slip along the fault during interseismic periods (blue, every one month) and during SSEs (red, every one day); black lines depict the start and end of SSEs.

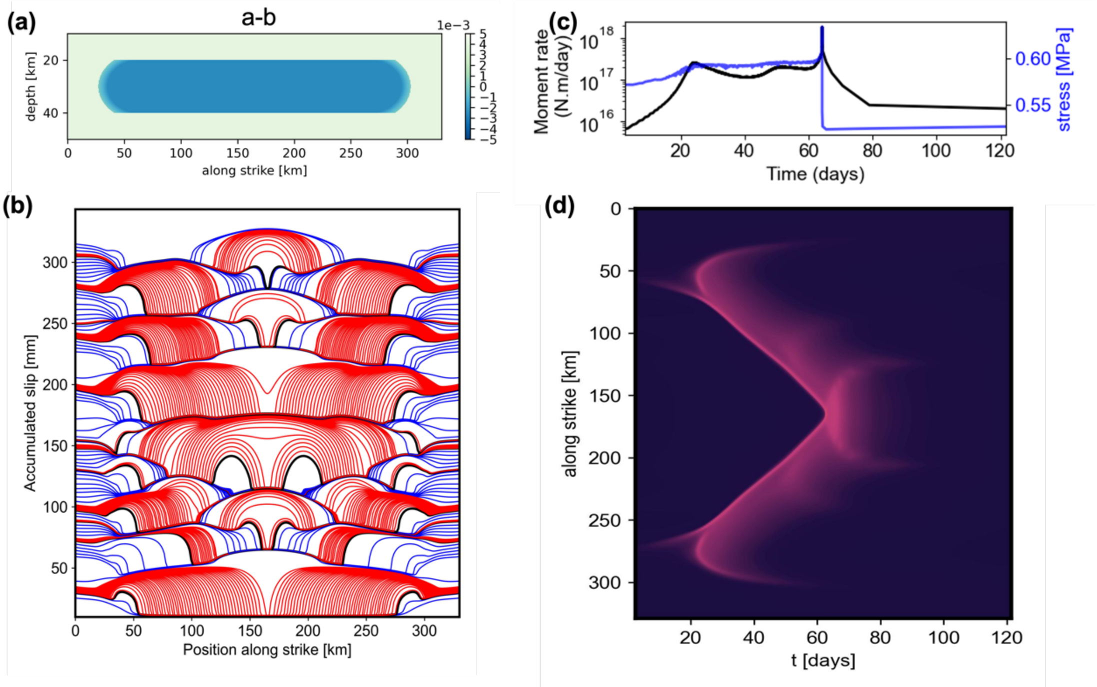

Figure S18. **a-b frictional homogeneous model.** (a) model setting. (b) accumulated slip along the fault during interseismic periods (blue, every one month) and during SSEs (red, every one day); black lines depict the start and end of SSEs. (c) Temporal evolution of moment rate and maximum stress for the coalesced events. (d) Spatiotemporal evolution of slip rates following the coalescence of SSEs during the deceleration phase.

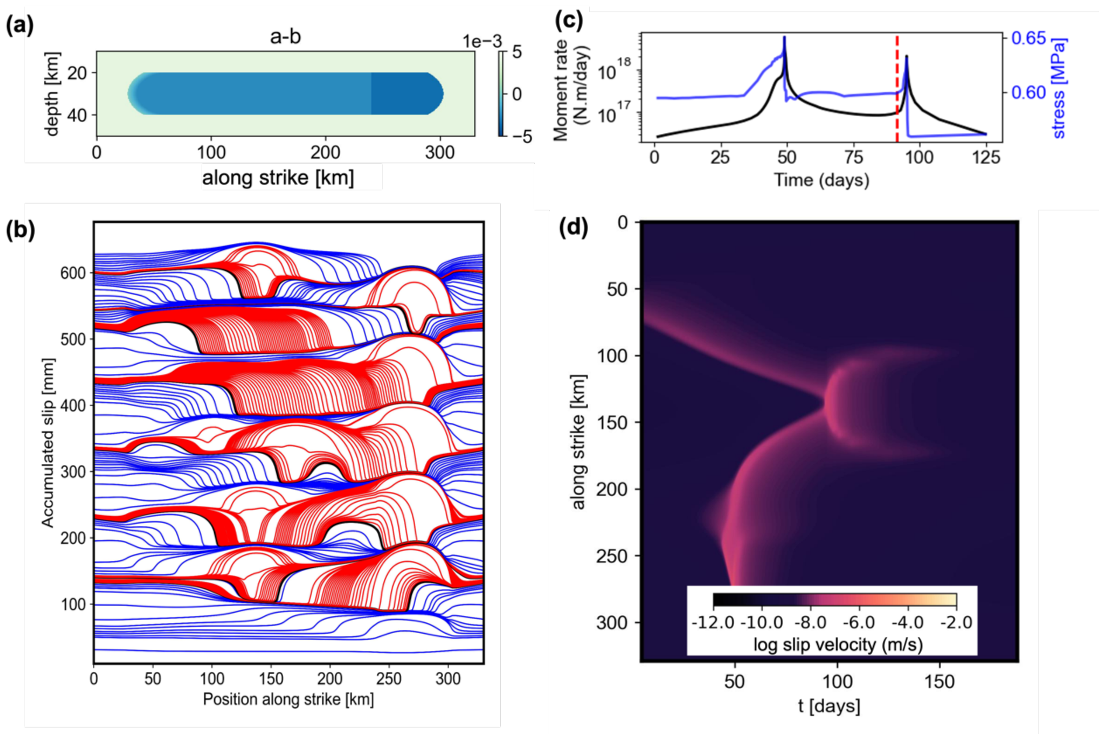

Figure S19. **a-b friction heterogeneous model (asymmetric)**. (a) model setting. (b) accumulated slip along the fault during interseismic periods (blue, every one month) and during SSEs (red, every one day); black lines depict the start and end of SSEs. (c) Temporal evolution of moment rate and maximum stress for the coalesced events. (d) Spatiotemporal evolution of slip rates following the coalescence of SSEs during the deceleration phase.

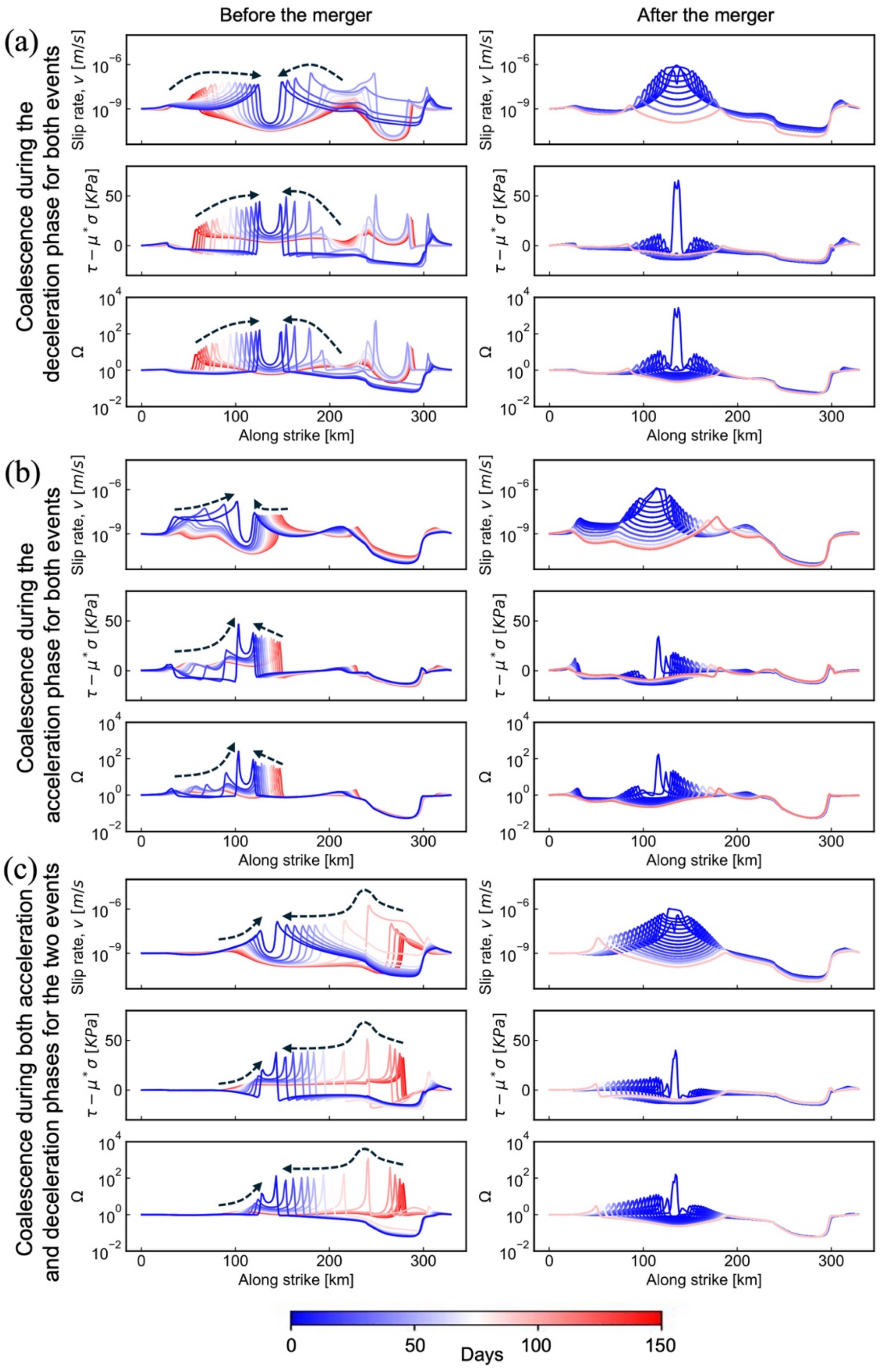

Figure S20. **The evolution of slip rate, stress, and fault state along the fault strike before and after the merger of two SSEs during different phases of coalescence for model in Figure S19.** The left column shows the snaps of the system before the merger, while the right column shows the snaps of the system after the merger. (a), the coalescence during the deceleration phase for both events is shown, with the top row displaying the slip rate, the middle row showing the shear stress τ with respect to a

reference stress value $\mu^*\sigma$, and the bottom row representing $\Omega = V\theta/D_c$. Arrows indicate the propagation direction of the peak values in each plot. The color represents the time relative to the merging time point. (b), the coalescence during the acceleration phase for both events. (c), the coalescence during both the acceleration and deceleration phases for the two events.

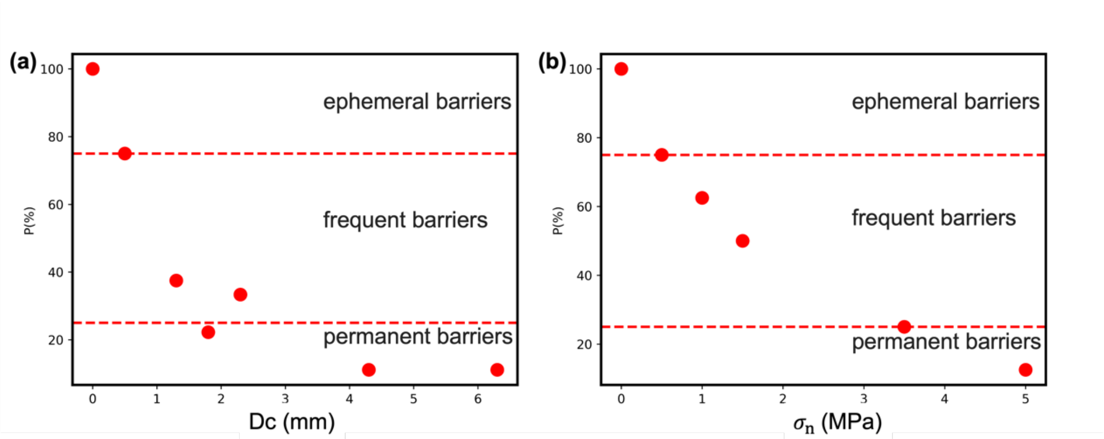

Figure S21. (a) The likelihood (P in percentage) of SSEs ruptures crossing a barrier is plotted as a function of Dc contrast in the central patches for Model I. The horizontal red lines delineate barriers into three categories: permanent ($P \leq 25\%$), frequent ($25\% < P < 75\%$), and ephemeral ($P \geq 75\%$) [8]. (b) The probabilityof SSEs ruptures crossing a barrier is presented as a function of the σ_n contrast in barriers for Model II.

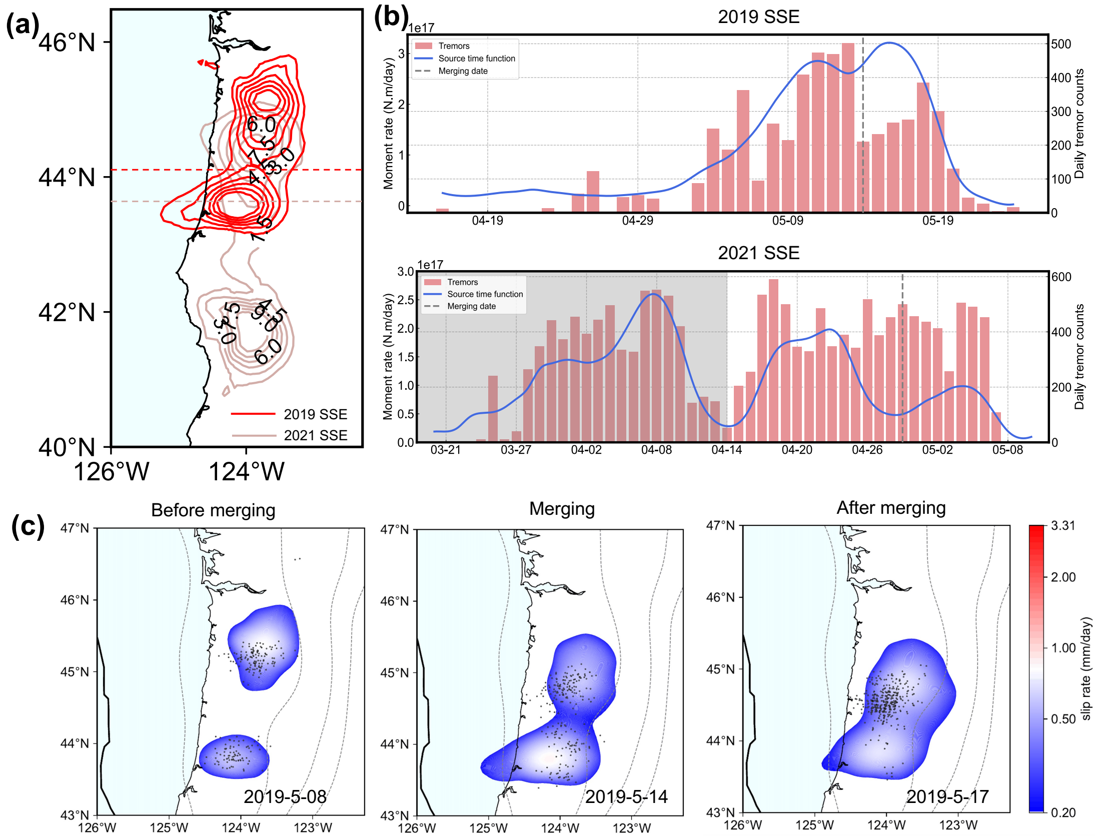

Figure S22. **Comparison of SSEs in 2019 and 2021.** (a) Accumulative slip distribution, with the red solid line representing the cumulative slip of the 2019 SSE, the brown solid line representing the cumulative slip of the 2021 SSE. The red dashed line indicate the latitude of SSE coalescence in 2019, and the brown solid line indicate the latitude of SSE coalescence in 2021. (b) Moment rate curves for the two events, with the gray dashed line indicating the coalescing time. (c) Snapshots before and after SSE coalescence in 2019, with black scatter points representing the distribution of tremors.

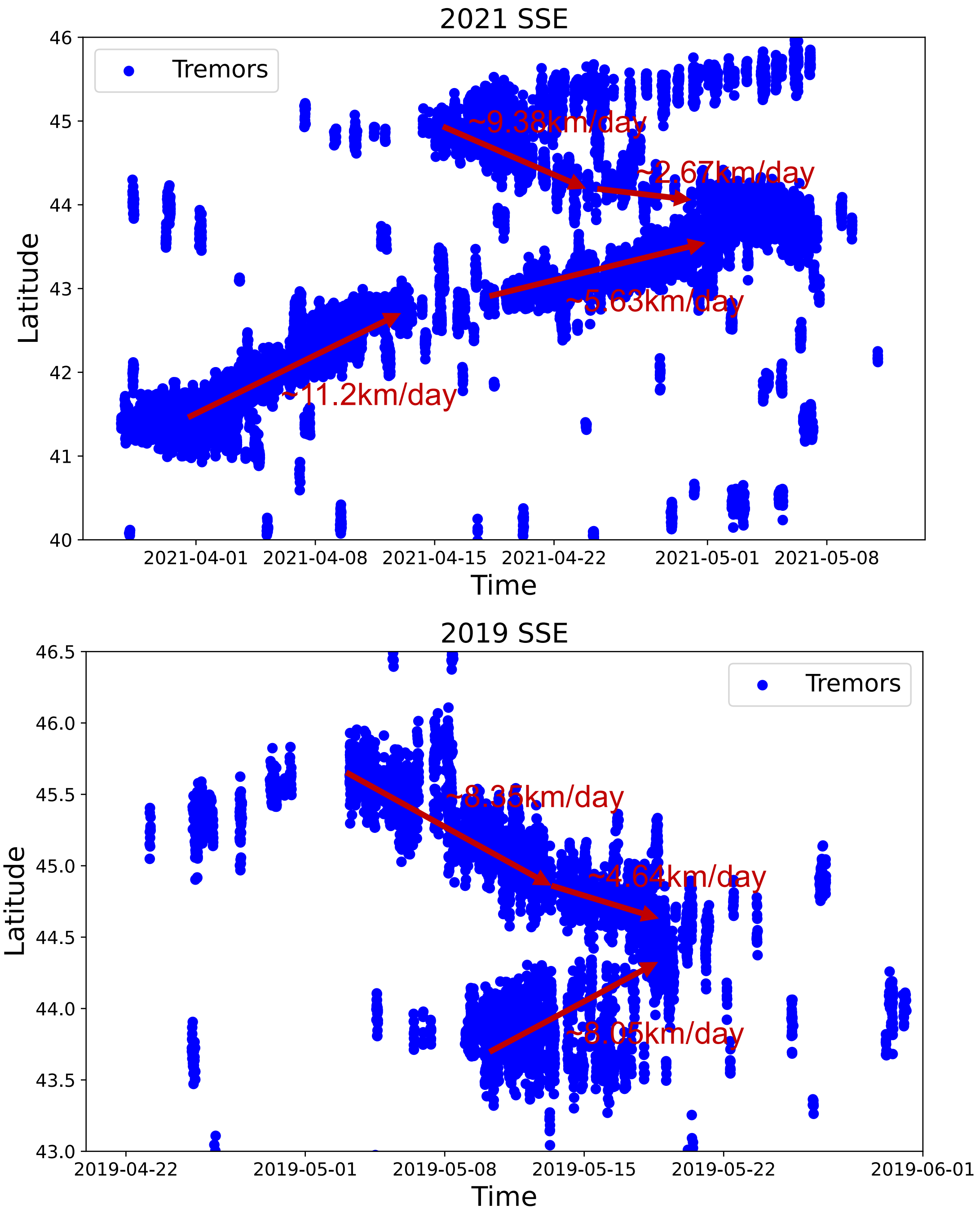

Figure S23. Temporal and spatial evolution of tremors in 2019 and 2021.

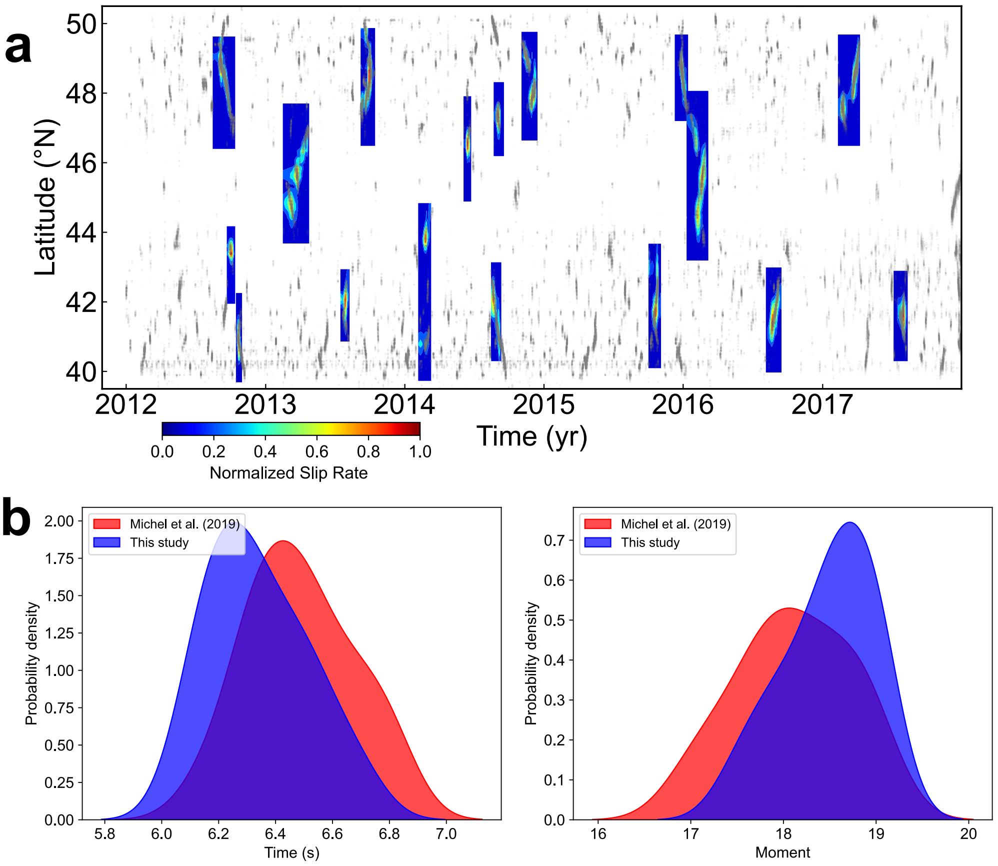

Figure S24. **The consistency of the two catalogs.** a, The spatiotemporal distribution of common SSEs, with slip rates normalized. Gray dots represent the spatiotemporal distribution of tremors from the PNSN catalog. b, Probability density distribution in the duration ($\log_{10}$[duration(s)]) and moment ($\log_{10}$[moment (s)]) of common SSEs between the two catalogs.

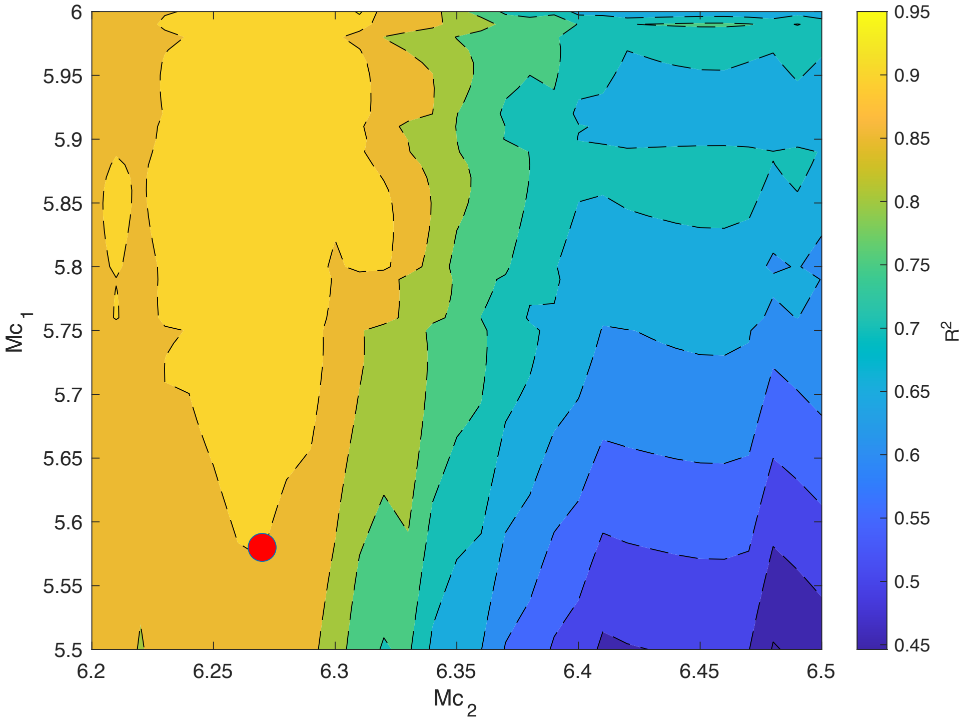

Figure S25. **The goodness of fit R^2.** The red dot is the magnitude at which 90% of the observed data are modeled by the power law fit. In the red dot, M_{c1}=5.58 and M_{c2}=6.27.

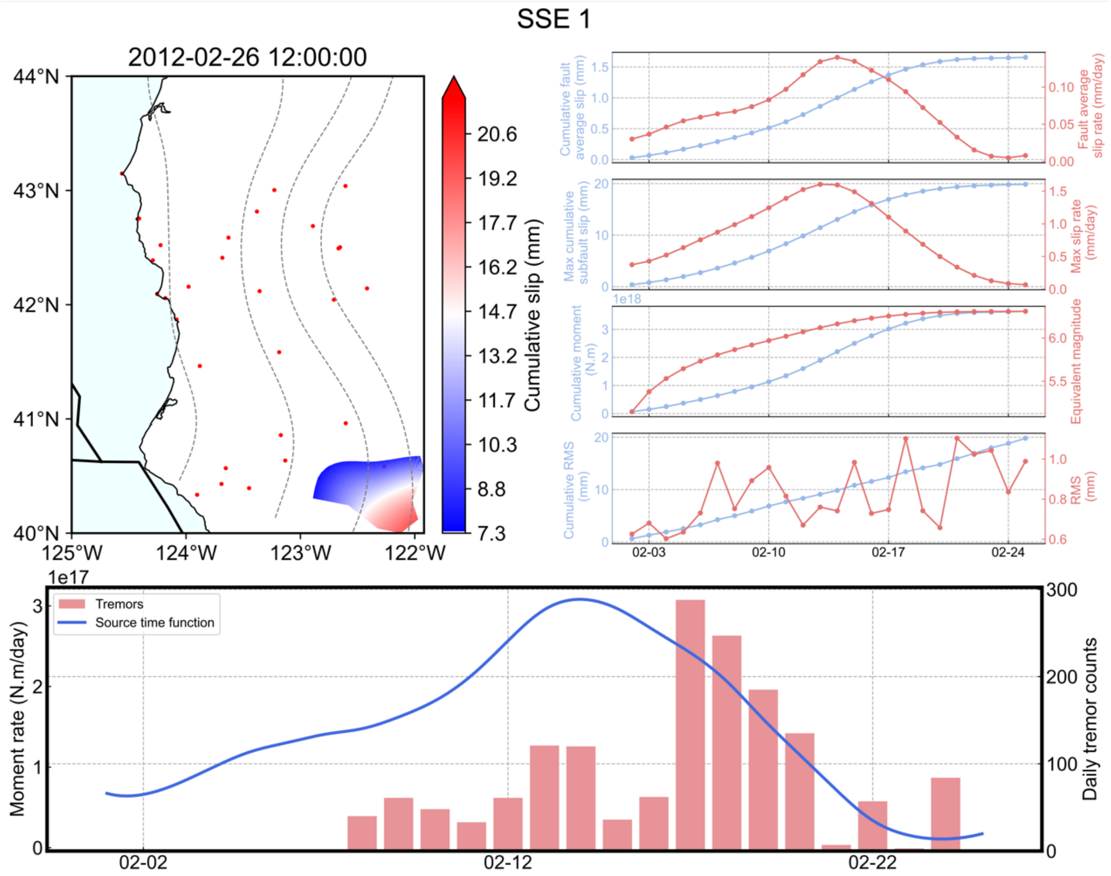

Figure S26: SSE 1. The top left panel shows the cumulative slip of the SSE. The red dots are the stations used in the kinematic slip inversion. The thick black lines are the plate boundaries and the dashed lines are the Slab2.0 iso-depth contours every 20 km[7]. The color bar scale is saturated for clarity. The top right panel indicates the time series analysis: The first row displays the cumulative average slip (blue) and average slip rate (red); The second row displays the cumulative slip of the subfault with the maximum slip (blue) and the slip rate of the sub-fault with the maximum slip (red); The third row displays the cumulative moment (blue) and the equivalent magnitude (red). The fourth row displays the cumulative RMS (blue) and RMS (red) of the data fitting. The bottom panel shows the source time function. The blue line indicates the moment rate curve and the red histograms show the tremor counts recorded by PNSN. For the event 1, we select 54 stations used for inversion. The event has a main peak of moment rates that precedes the peak in the number of tremors by three days. The slip occurs along the southern edge of the plate, constrained by the Slab2.0 fault model [7].

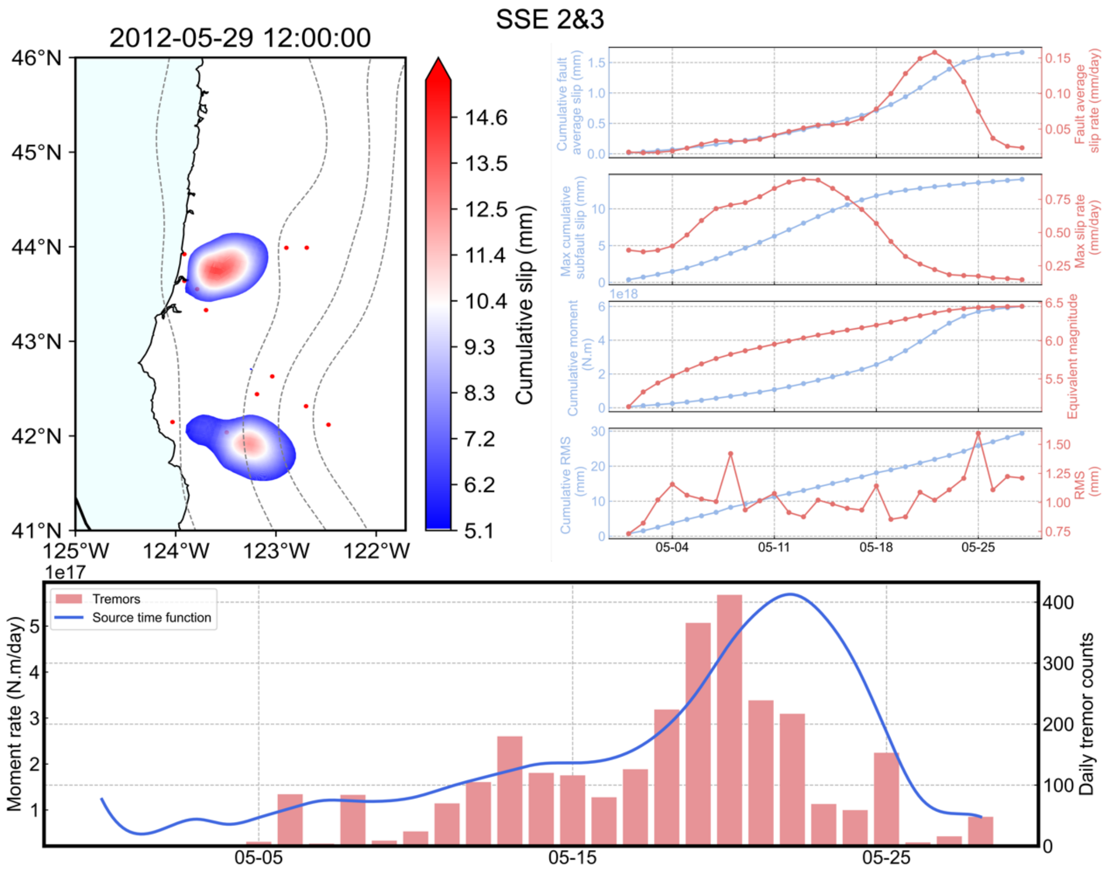

Figure S27: SSE 2&3. We select 13 stations used for inversion. This event contains two small SSEs that coincide with a continuous tremor burst. The moment is Mw 6.5. The event has a main peak of moment rates that follows the peak in the number of tremors by two days.

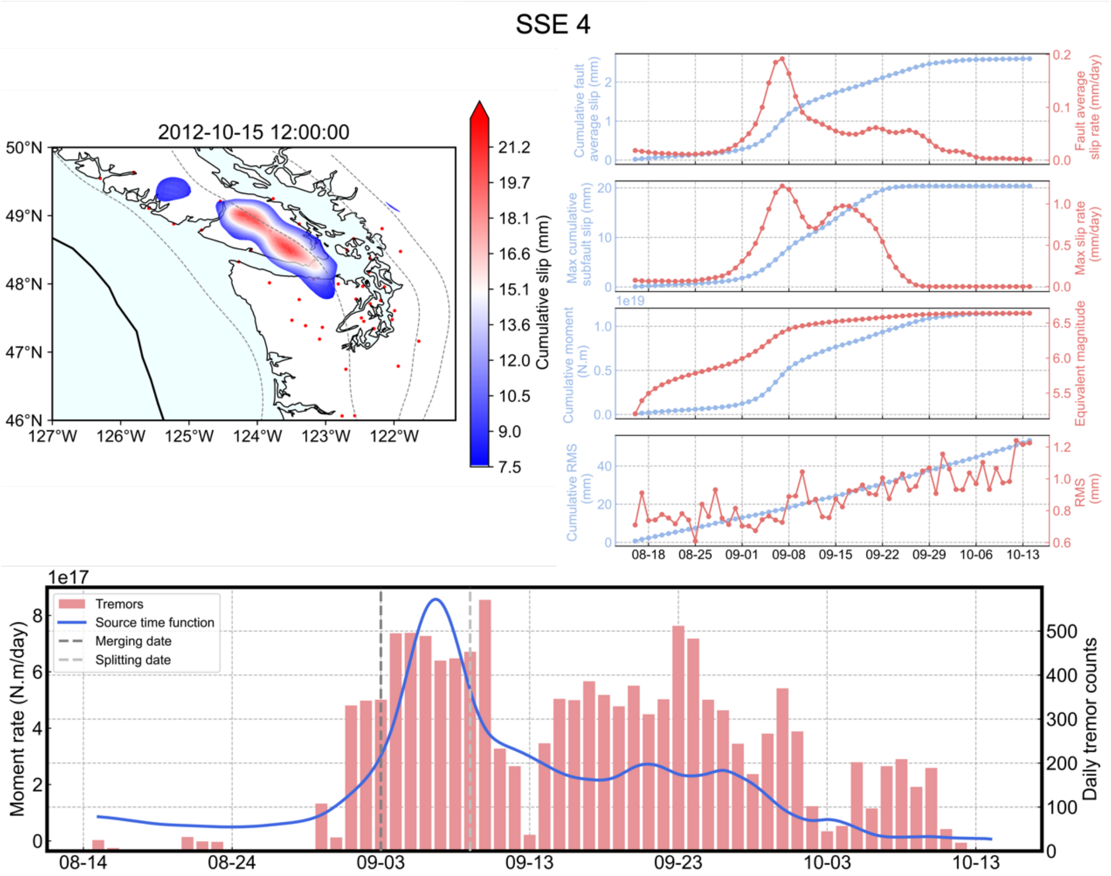

Figure S28: SSE 4. We select 43 stations used for inversion. This event exhibits three types of slow slip event behaviors including coalescence, segmentation, and migration. At the onset, a slip occurred in the southern part of Vancouver Island, soon followed by tremors. Then slip occurred in the middle part of Vancouver Island coinciding with a continuous tremor burst. On September 3rd, the two patches merged. During this phase, the moment rate release rises by a factor of 3 in 4 days, and the daily maximum slip increases by a factor of 2 in 4 days. Subsequently, tremors migrated from the coalesced region along the strike to the south and north, with the slip rate gradually decreasing, until on September 9th, it split into two separate slip patches again. The northern patch gradually diminished, while the southern patch continued to migrate along the strike until it stopped at 47.5°N and eventually dissipated. the sub-fault with the maximum slip has two peaks, one resulting from the coalescence of slip and the other from the migration of slip.

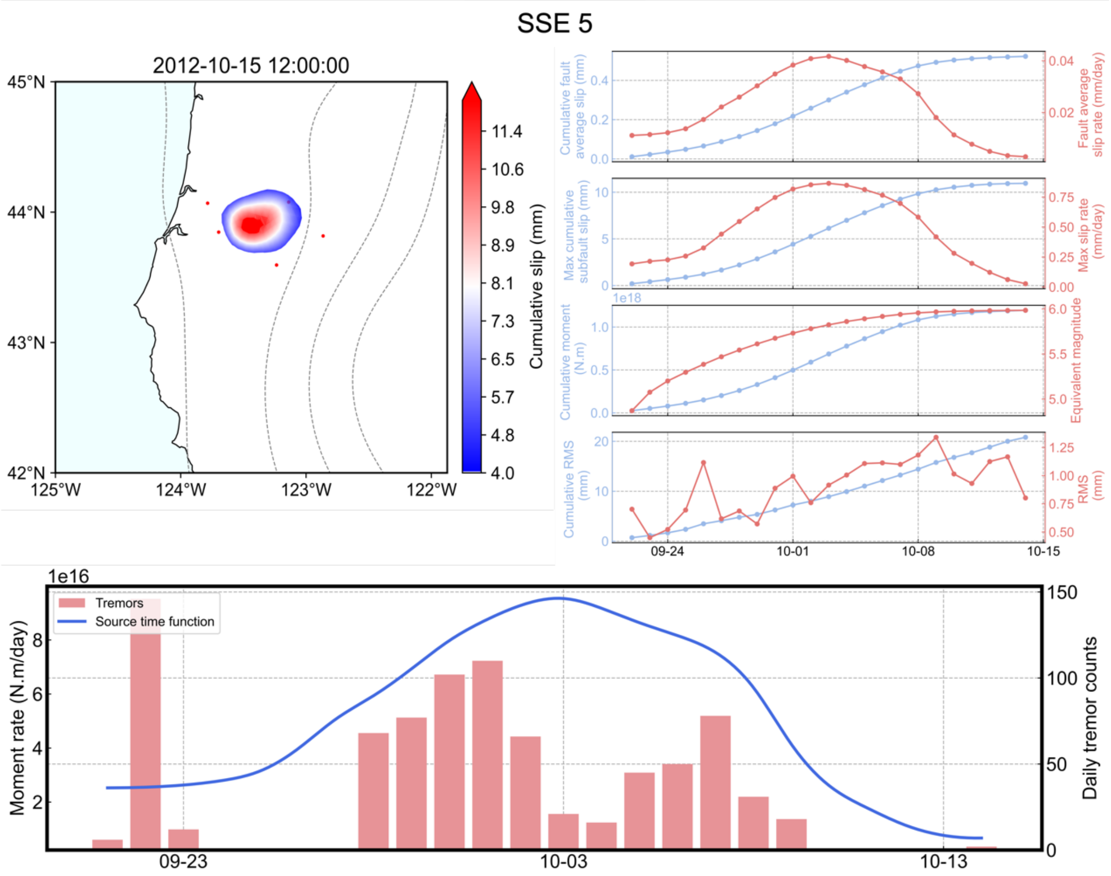

Figure S29: SSE 5. We select 7 stations used for inversion. This is a small event and the magnitude is Mw 6.

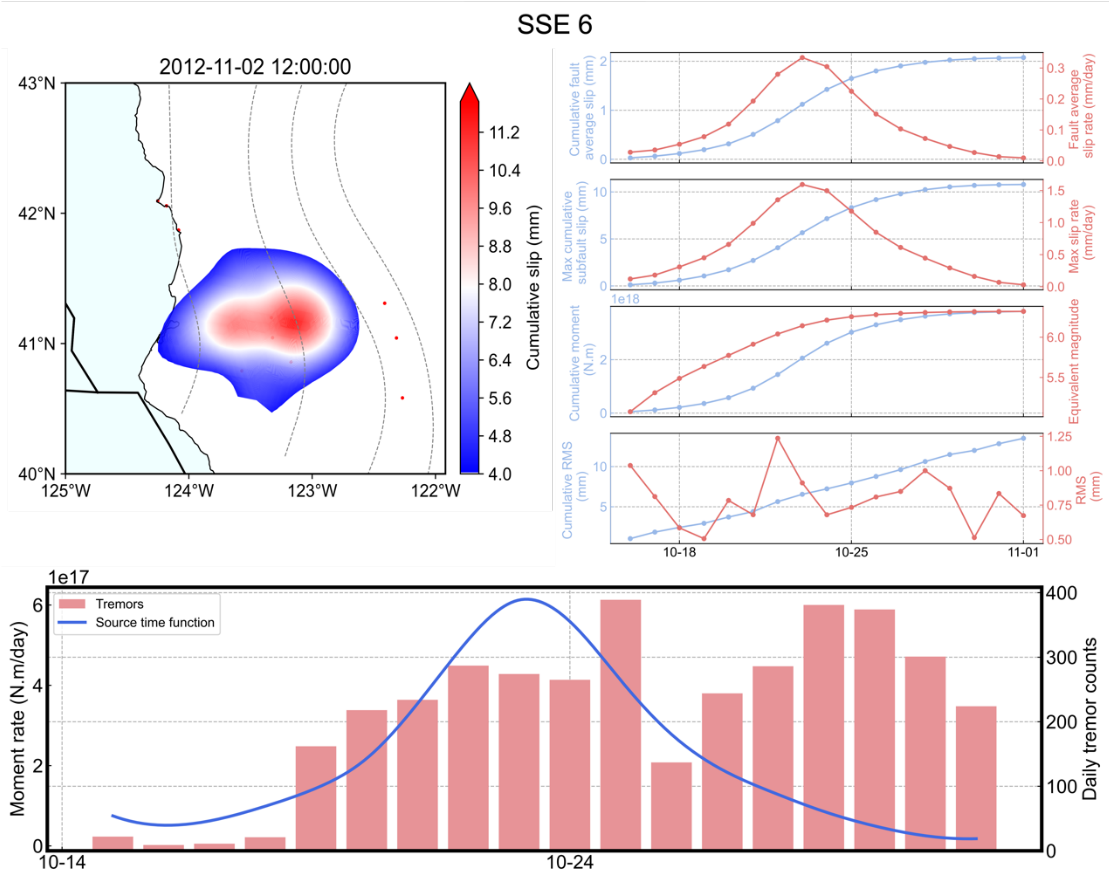

Figure S30: SSE 6. We select 10 stations used for inversion. This SSE has 1 peak of moment rate release and the magnitude is Mw 6.6. After the slip ceased, tremors without slip persisted in the south.

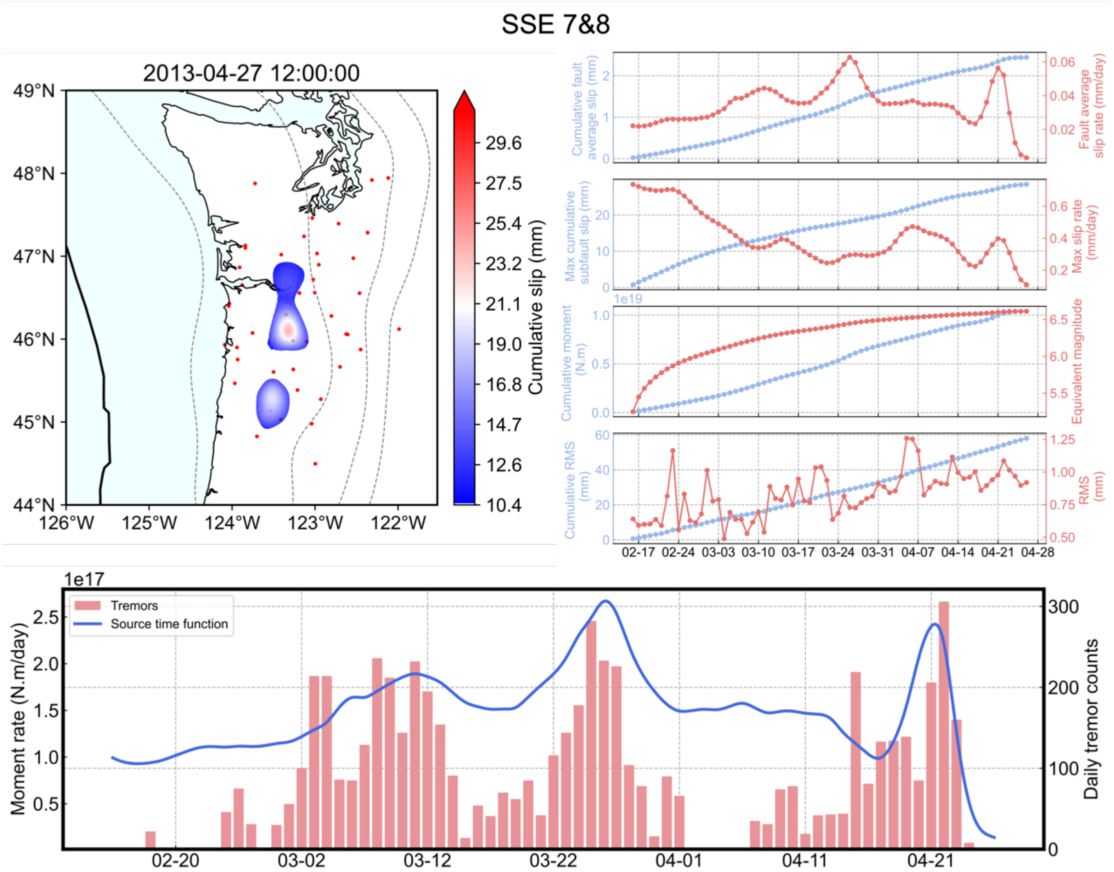

Figure S31: SSE 7&8. We select 46 stations used for inversion. This SSE has 3 peaks of moment rate release coincides with a continuous tremor burst and the magnitude is Mw 6.6. At the onset, the slip patch in the south appeared and diminished normally. After reaching its peak, the slip patch in the north gradually migrated northward along the strike during the decaying phase of the slip rate. The migration rate is 5.6 km/day, and during this migration process, there were days when no tremors occurred. After the migration stopped, a large number of tremors occurred in the northern part of the slip, followed by a secondary slip peak in the same slip area.

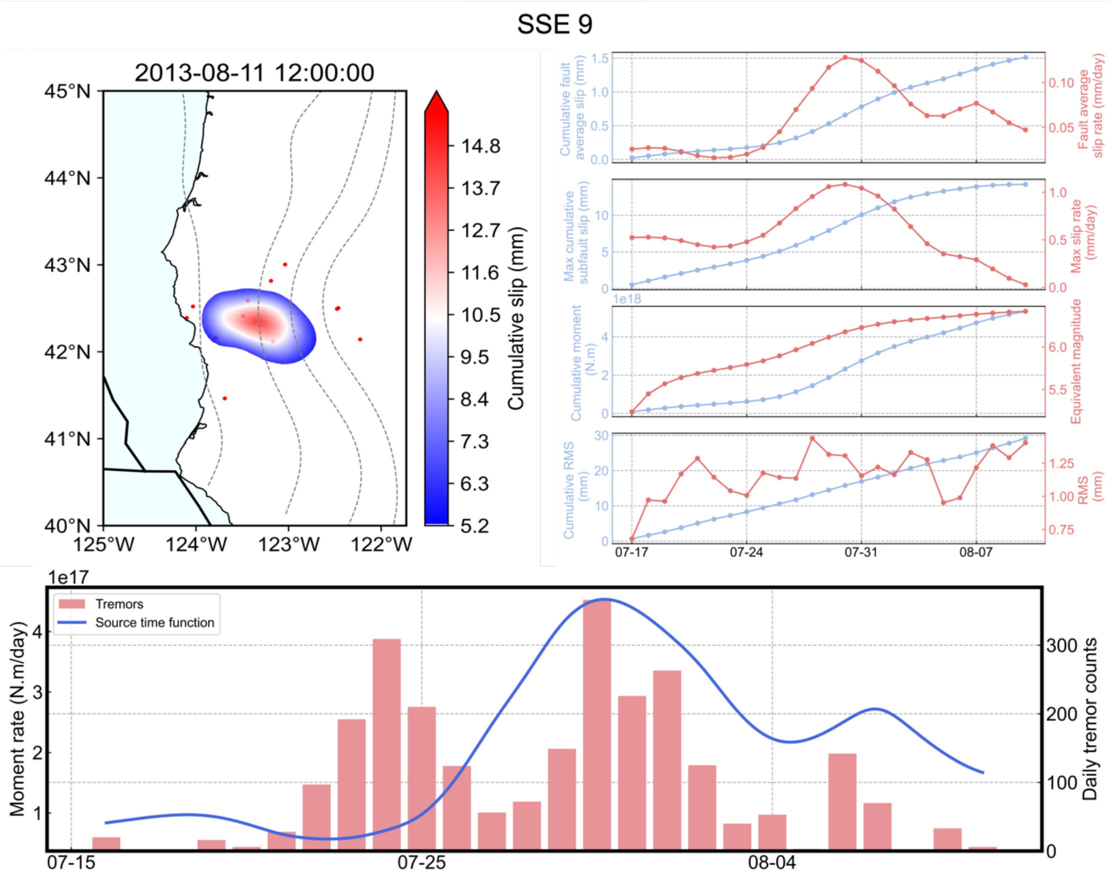

Figure S32: SSE 9. We select 12 stations used for inversion. This SSE has 1 peak of moment rate release. The peak of the slip rate coincides with the peak in tremor counts.

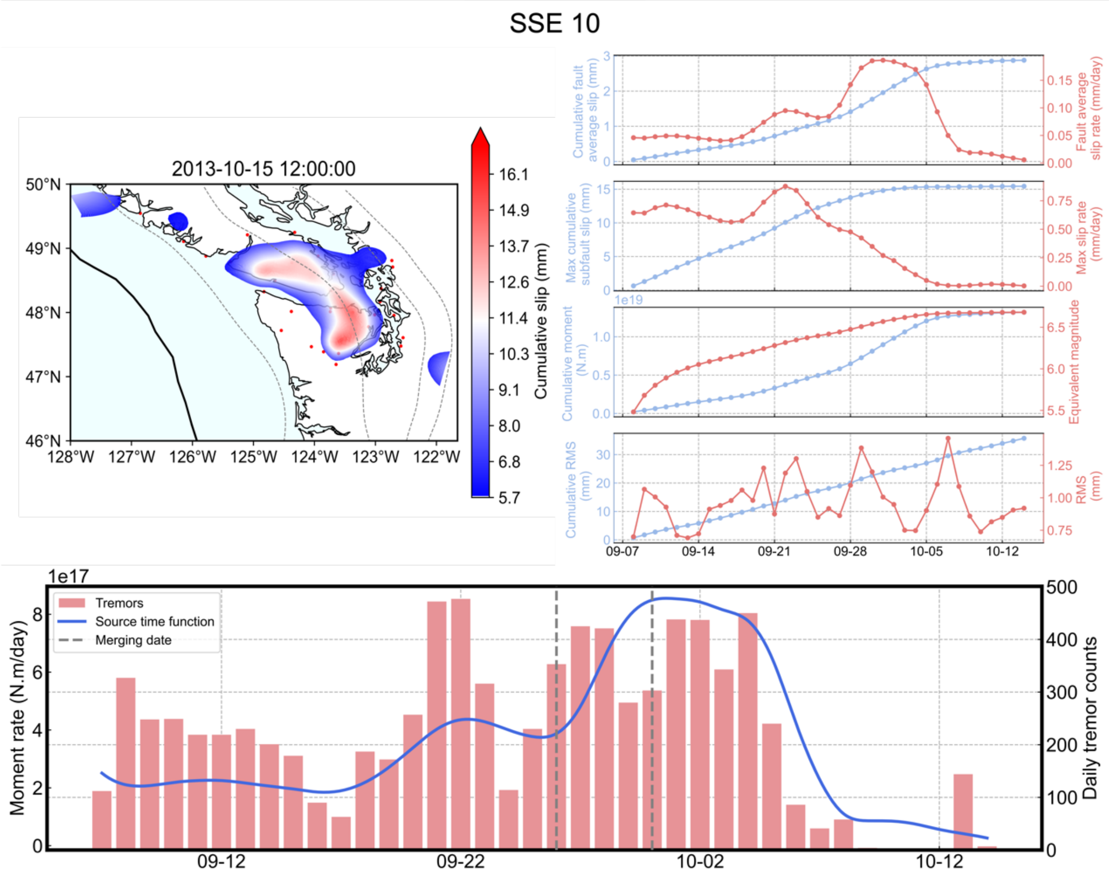

Figure S33: SSE 10. We select 35 stations used for inversion. Compared with the results of Bletery and Nocquet [9], Our results indicate that the magnitude is Mw 6.68, which is 0.08 greater than their value of 6.6. The results also show two times of slow slip fronts merging. During these periods, the rate of energy (moment) release significantly increases, validating the correctness of the inversion method. However, the moment curve only exhibits a single peak and does not show two peaks.

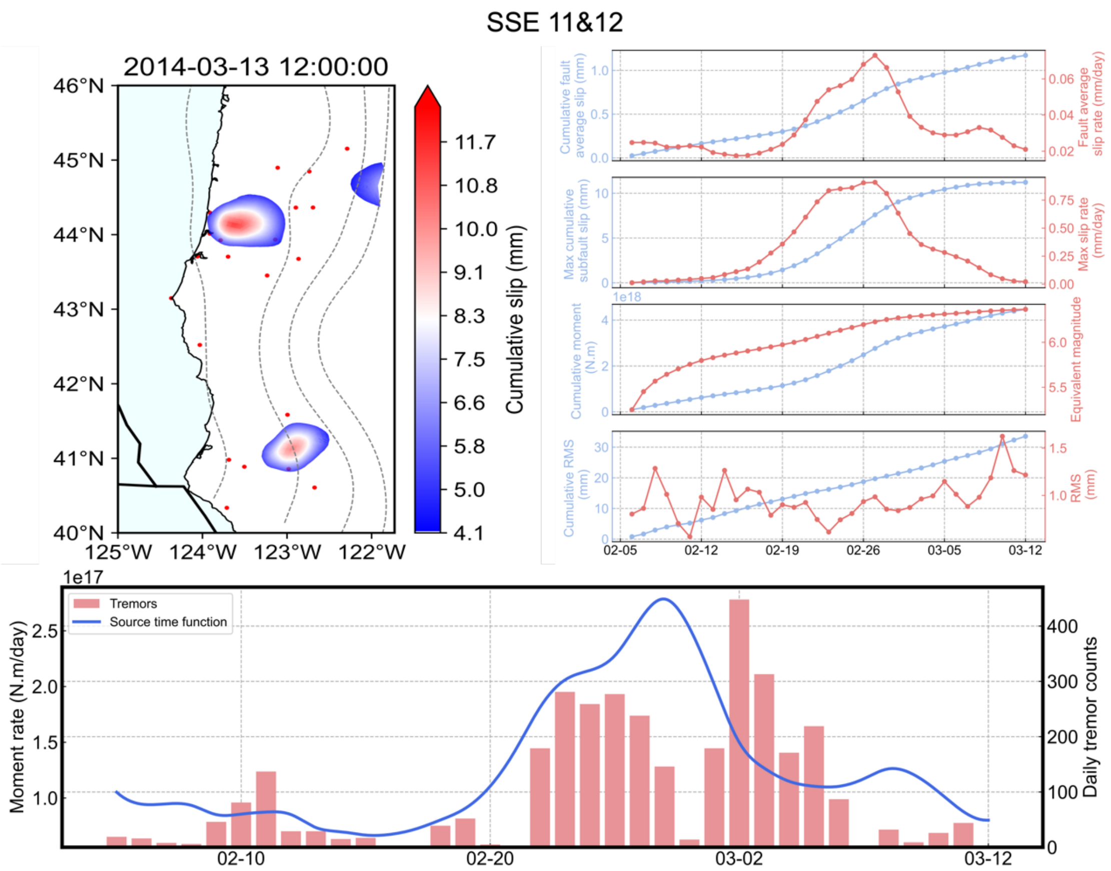

Figure S34: SSE 11&12. We select 21 stations used for inversion. This SSE consist of two small SSEs, and the primary slip is concentrated in the northern SSE.

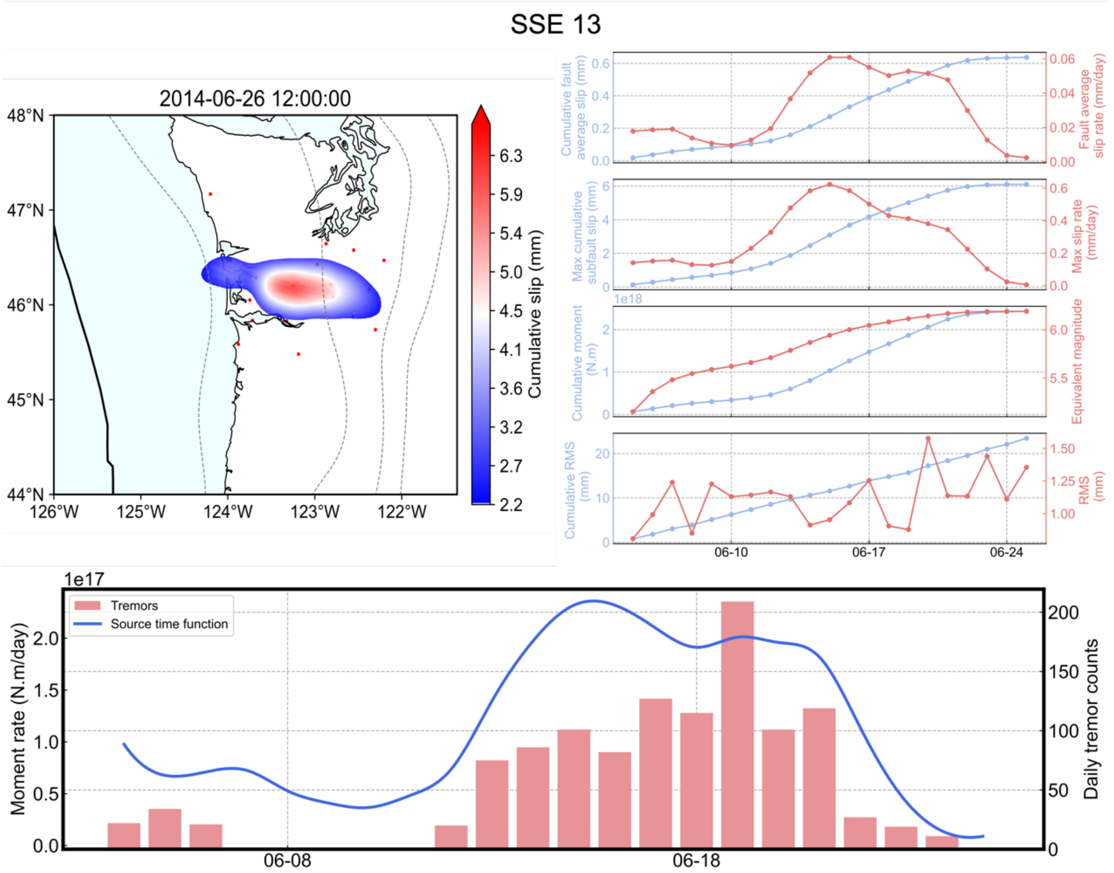

Figure S35: SSE 13. We select 19 stations used for inversion. The event has a main peak of moment rates that precedes the peak in the number of tremors by three days. Tremors migrate northward along the strike, while the center of slip remains nearly unchanged.

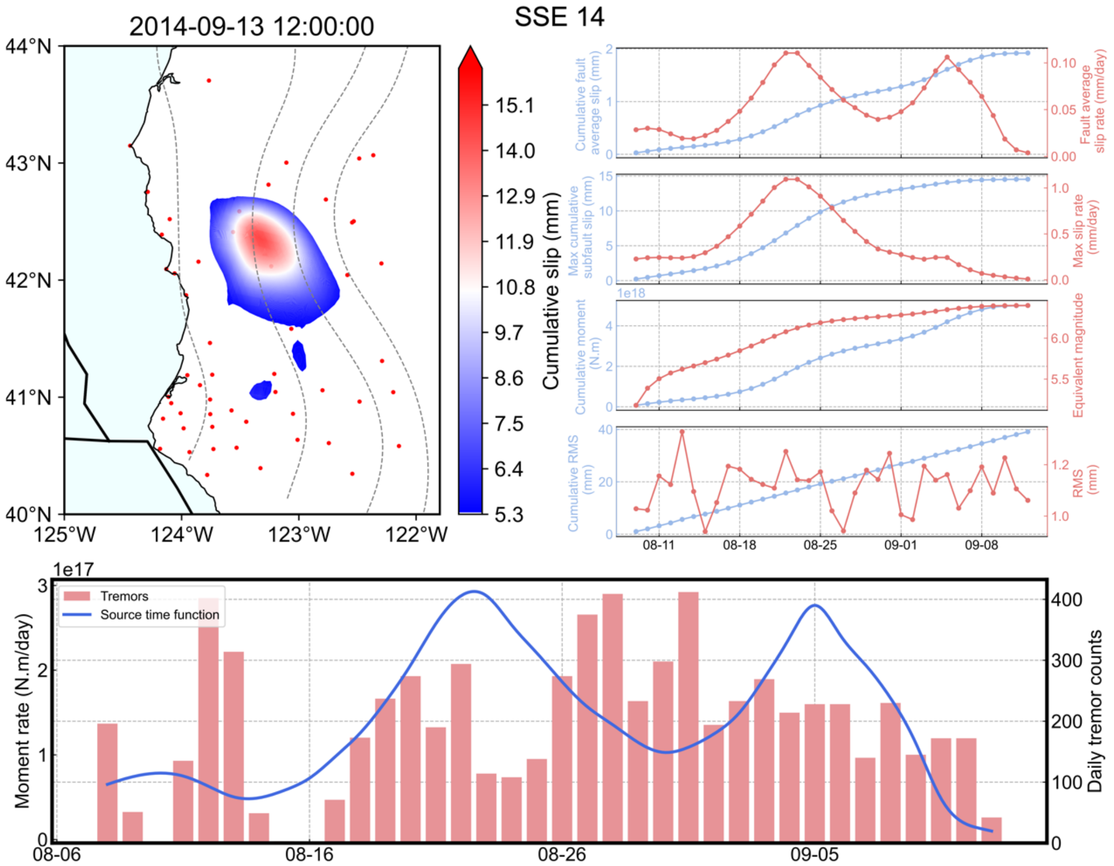

Figure S36: SSE 14. We select 68 stations used for inversion. This SSE has two main peaks of the moment rates. After reaching its peak on August 22nd, the slip rate entered a period of decline and gradually migrated southward along the strike. The migration speed of tremors is faster than that of slip, accompanied by an increase in tremor counts. The peak of the tremor count histogram corresponds to the migration period and lies between the two peaks of the moment rate curve. After the migration ceased, the moment rate curve entered a new peak.

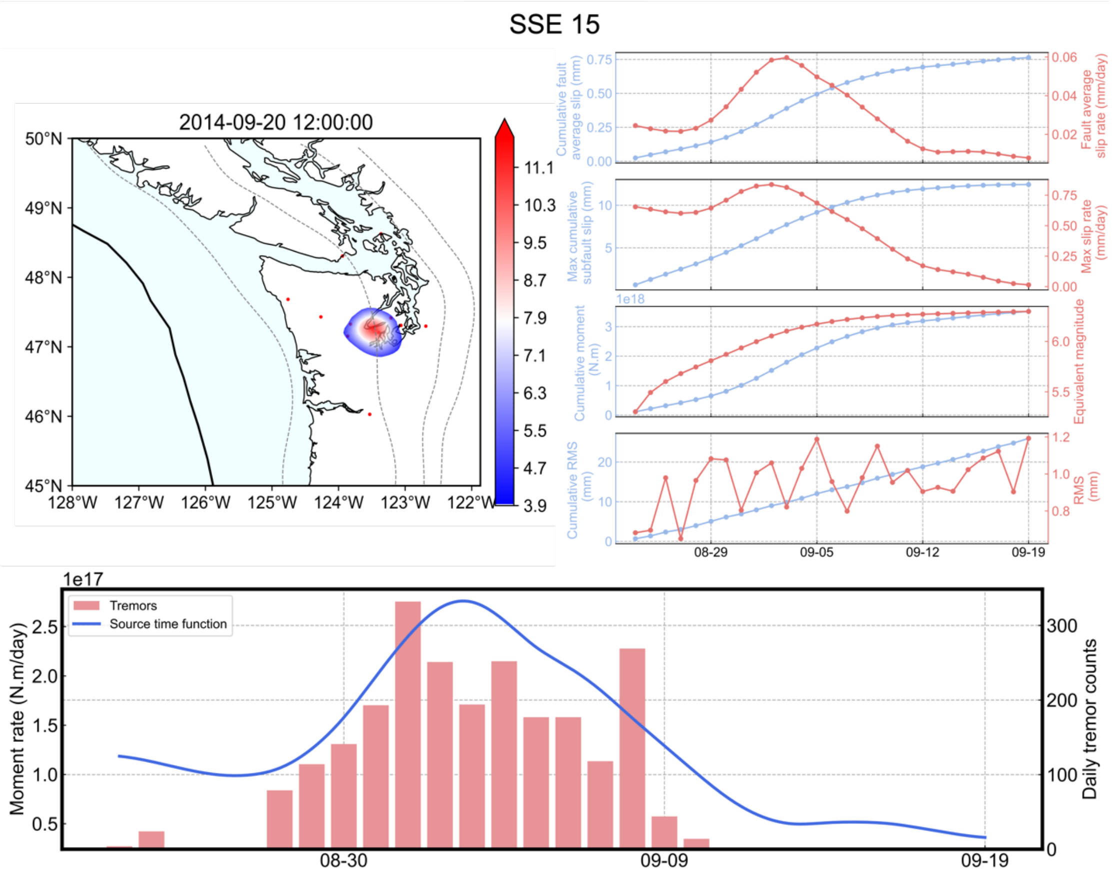

Figure S37: SSE 15. We select 11 stations used for inversion. The event has only one peak of moment rate and is accompanied by a tremor outbreak.

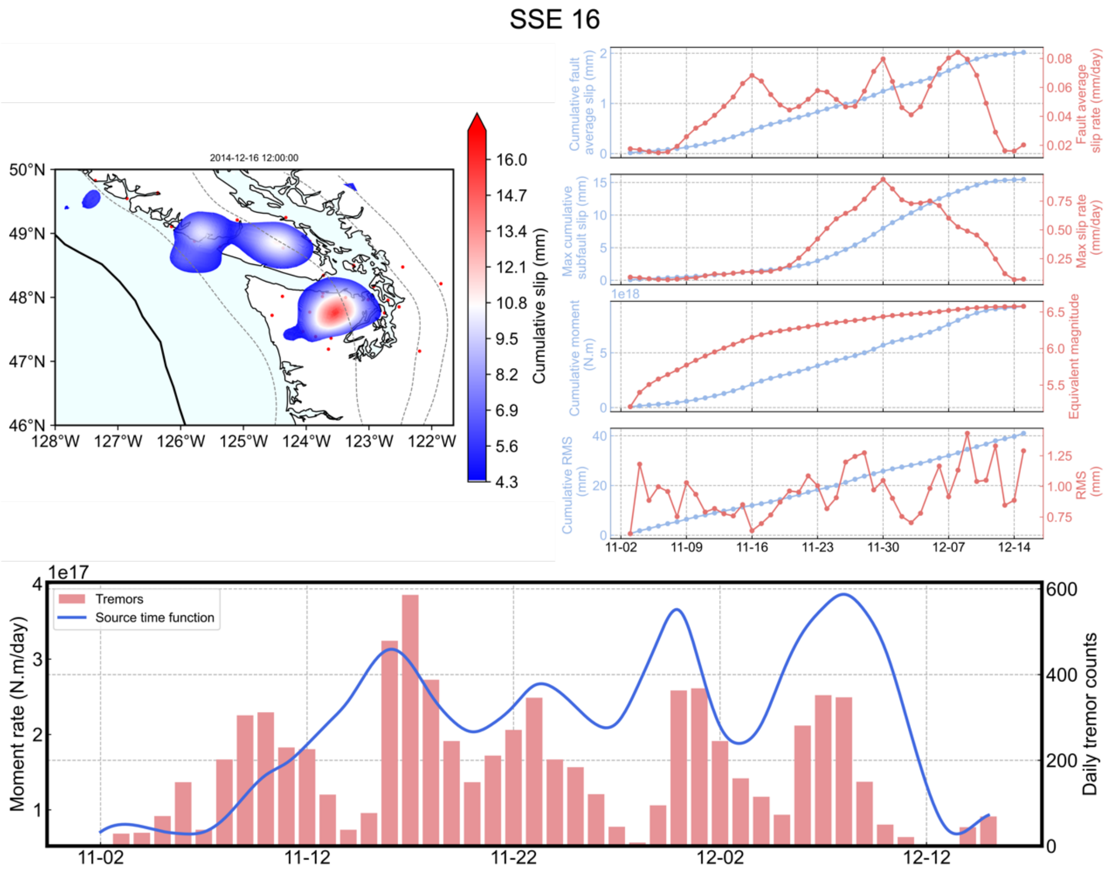

Figure S38: SSE 16. We select 28 stations used for inversion. The event has 4 peak of moment rate and is accompanied by a tremor outbreak.

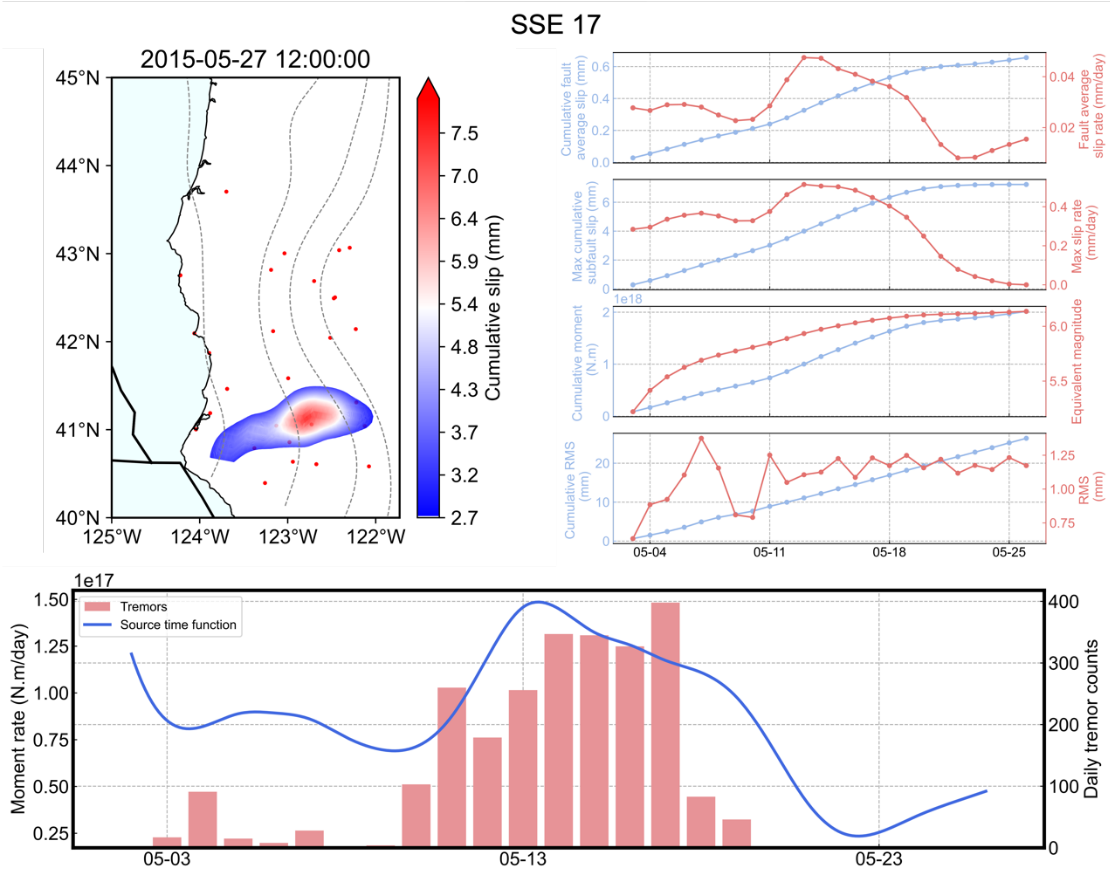

Figure S6: SSE 17. We select 44 stations used for inversion. The event has only one peak of moment rate and is accompanied by a tremor outbreak.

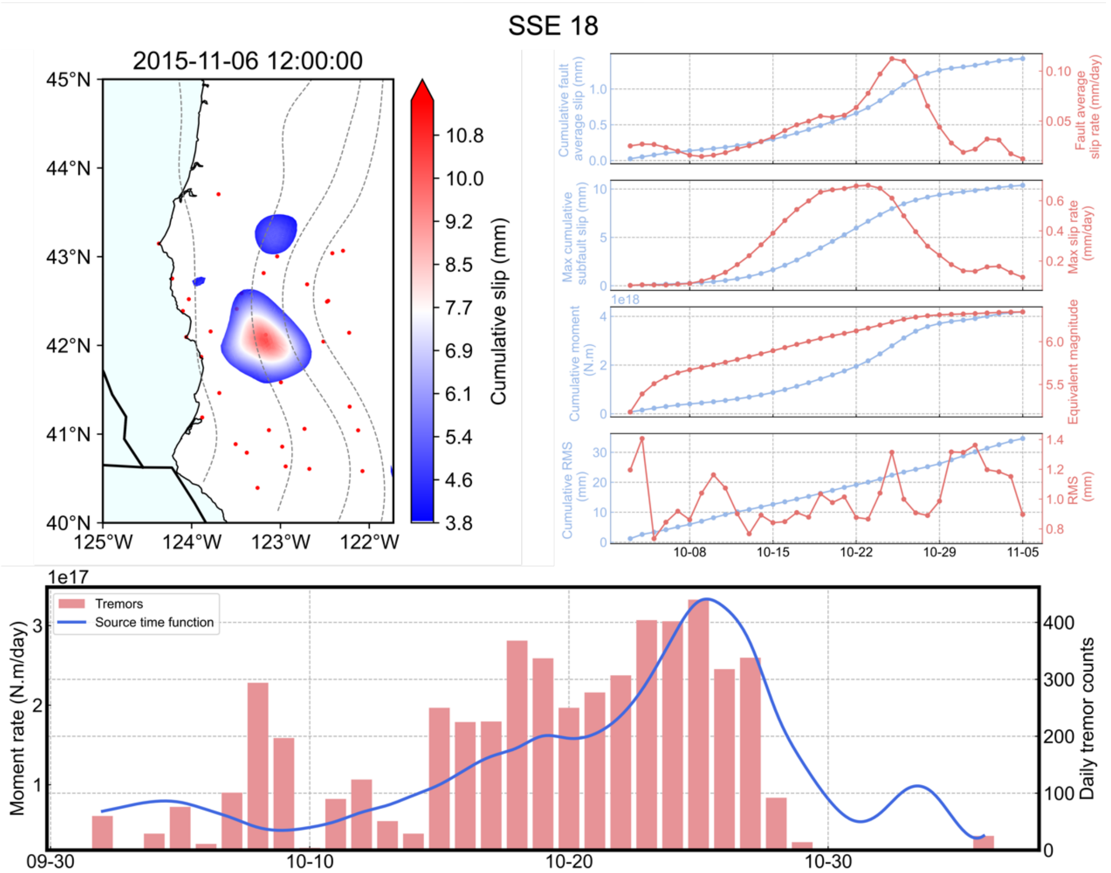

Figure S40: SSE 18. We select 50 stations used for inversion. This event has one main peak of moment rates. During the acceleration phase of slip rates, there is a trend of northward propagation along the strike.

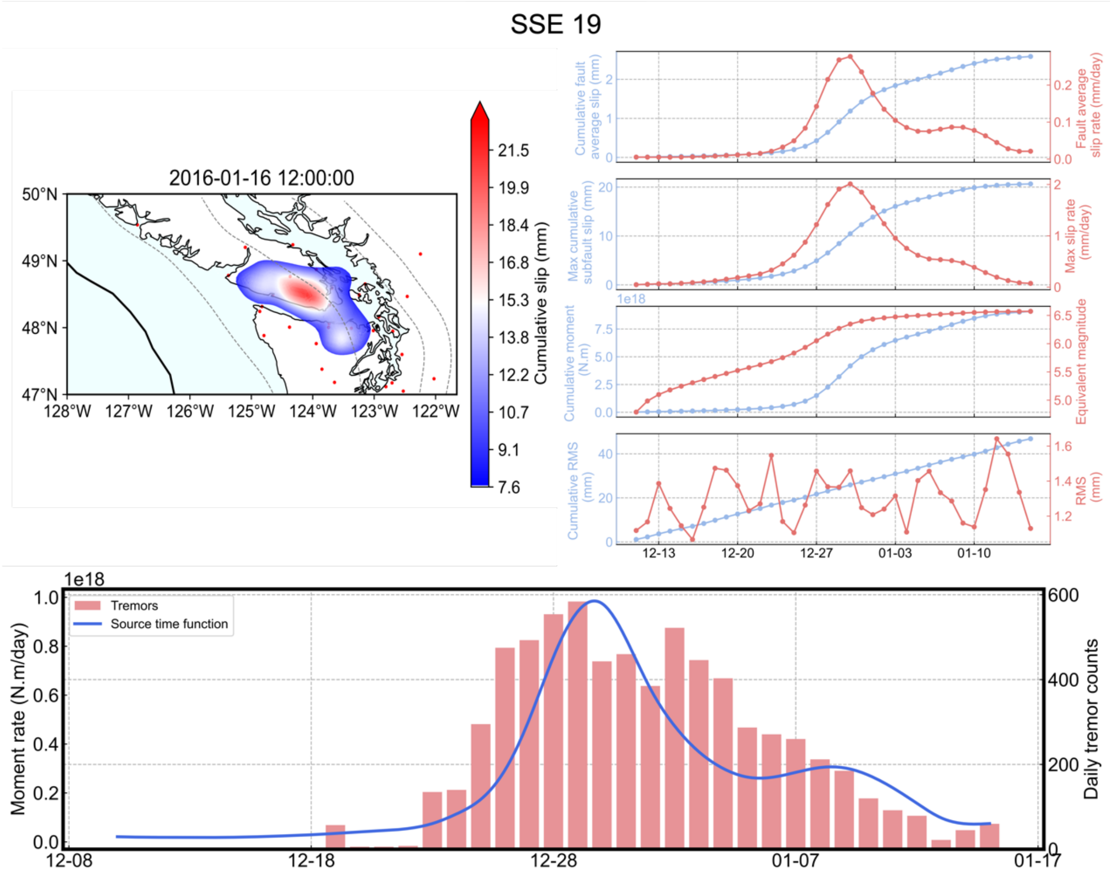

Figure S41: SSE 19. We select 37 stations used for inversion. This event has one main peak of moment rates. After reaching its peak, it migrates southward along the strike.

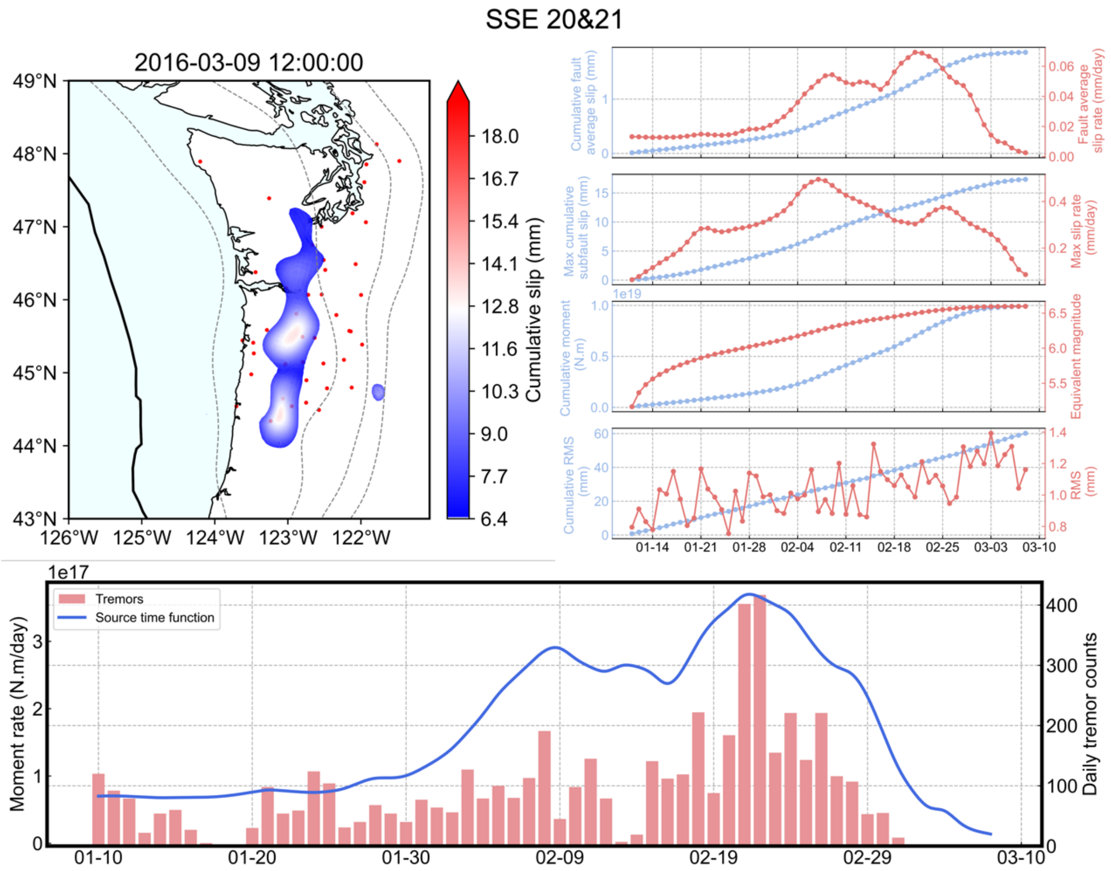

Figure S42: SSE 20&21. We select 43 stations used for inversion. This SSE exhibits two migration events, both occurring during the acceleration phase. The first event migrates southward along the strike at a speed of 2.7 km/day, while the second event migrates northward along the strike at a speed of 7.8 km/day. Both events ultimately halt near the Columbia River, which corresponds to the dividing line of zones 3 and 4 in the paper of Michel, et al. [10].

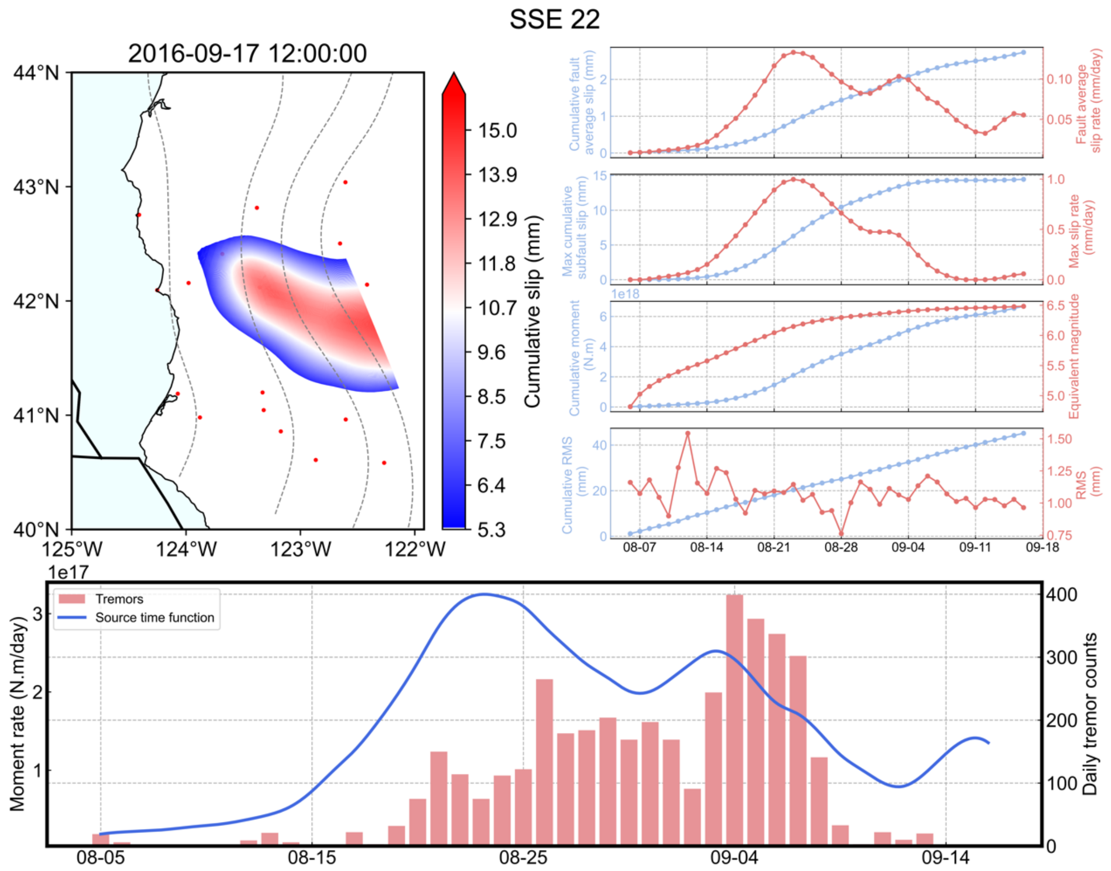

Figure S43: SSE 22. We select 18 stations used for inversion. This SSE has one main peak of moment rate followed by a smaller peak. Slips and tremors propagate in both the strike and dip directions.

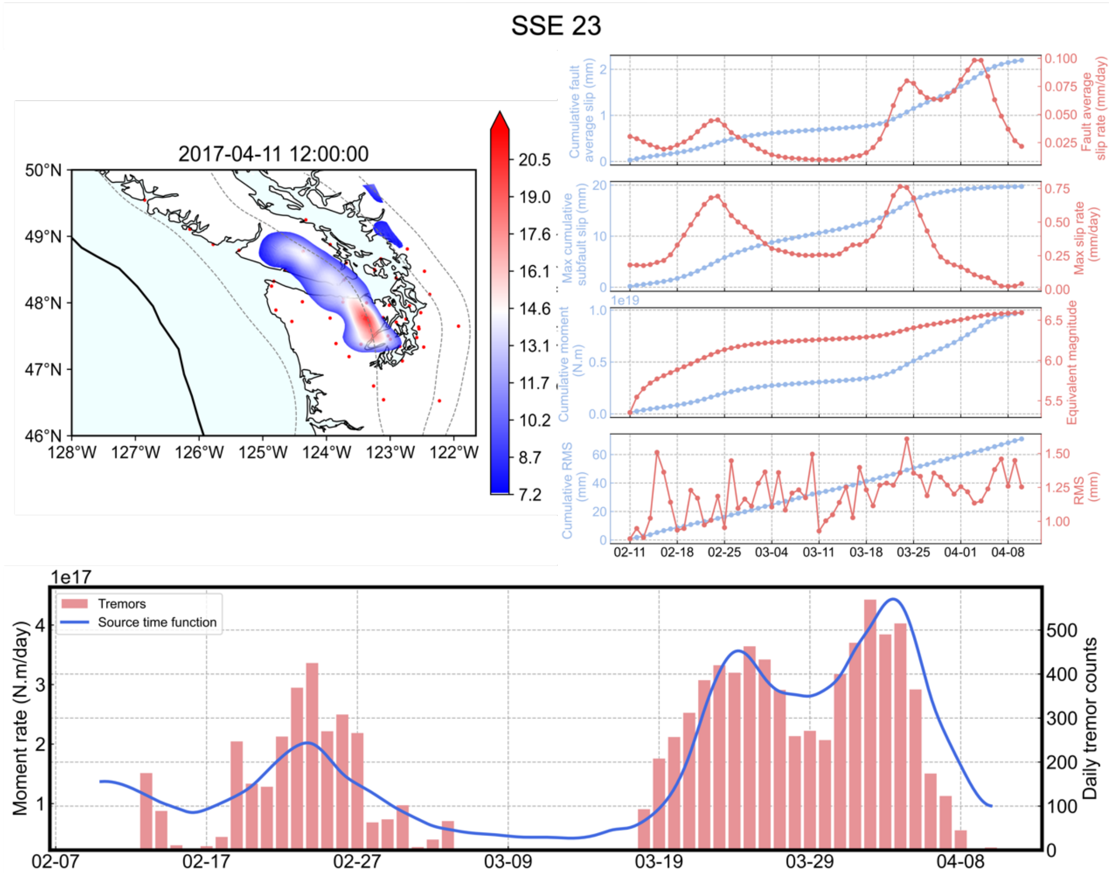

Figure S44: SSE 23. We select 50 stations used for inversion. This SSE is a continuous migration event and has three peaks of moment rate. The slip positions of peaks 1 and 2 can be used to divide the zone of SSEs into discrete segments, which correspond to the zones 1 and 2 in the paper of Michel, et al. [10]. However, the slip positions of peaks 2 and 3 can divide zone 1 in the paper of Michel, et al. [10] into two parts. This phenomenon is not only evident in this event but also event 11.

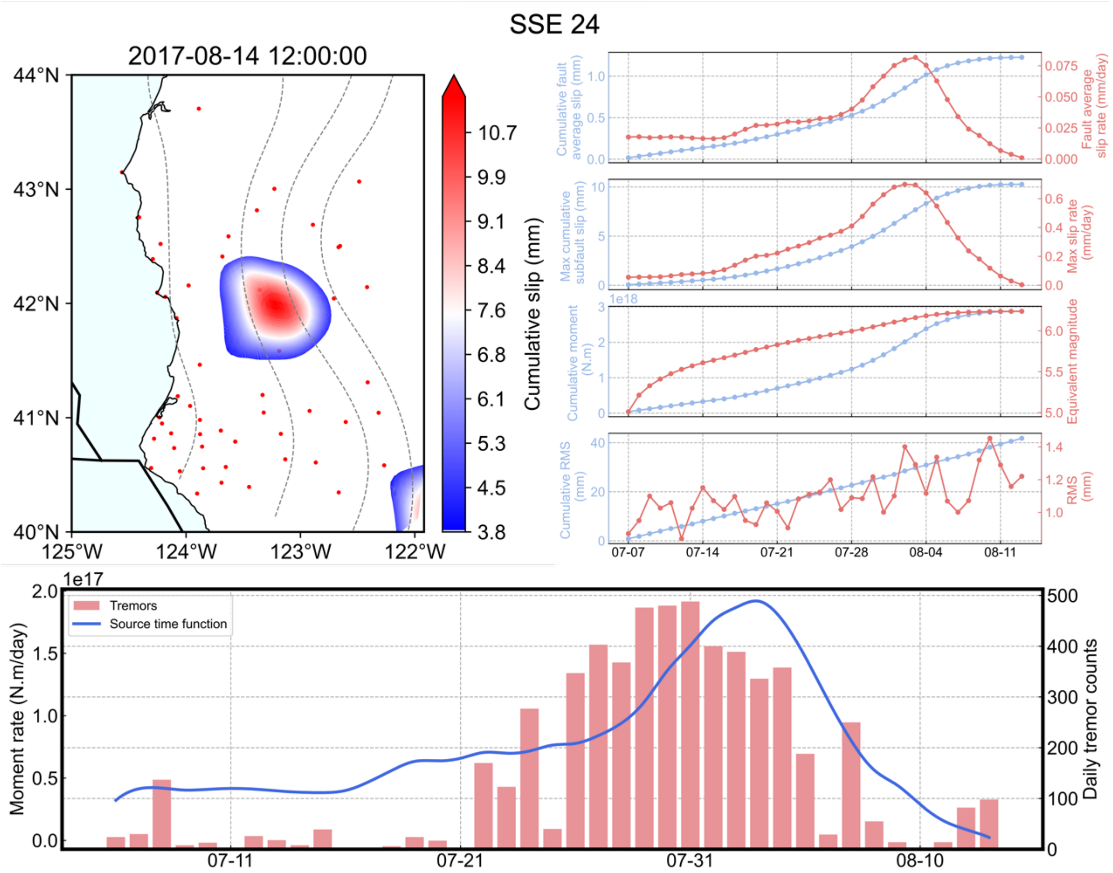

Figure S45: SSE 24. We select 68 stations used for inversion. This event has one peaks of moment rate coinciding with a continuous tremor burst.

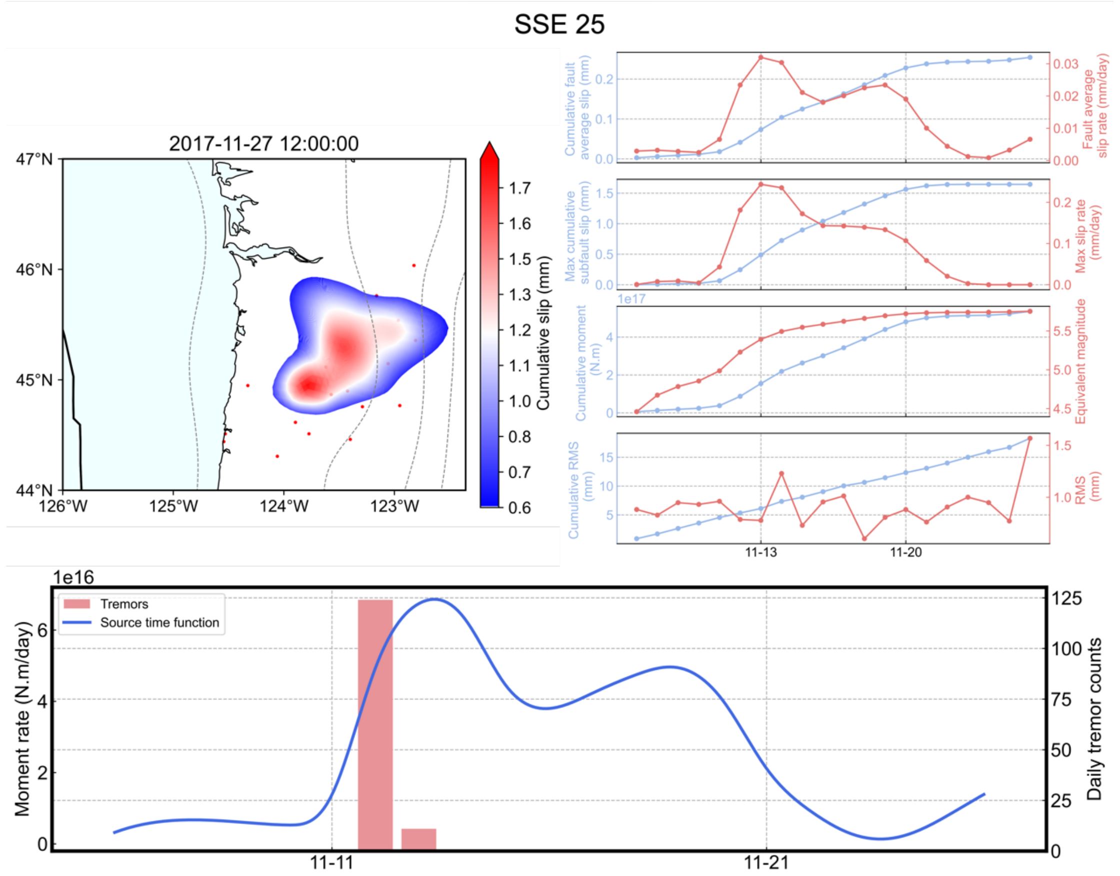

394

395 **Figure S46: SSE 25.** We select 18 stations used for inversion. This is a small event and

396 the magnitude is Mw 5.7. Tremors occur only on two days.

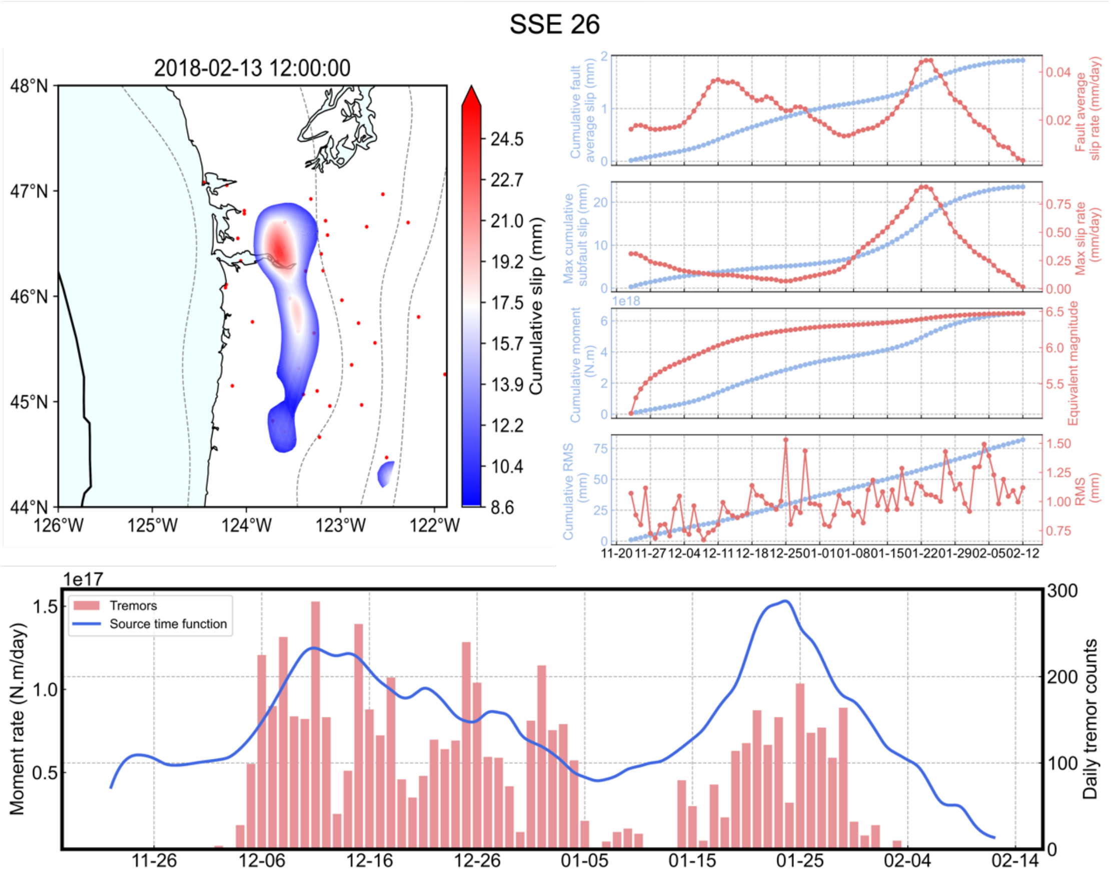

Figure S47: SSE 26. We select 50 stations used for inversion. This SSE is a continuous migration event and has 2 main peaks of moment rate. Near the first peak of the moment rate curve, there are four smaller peaks corresponding to peaks in the tremor count histogram. The first smaller peak coincides with the peak in tremor counts, the second peaks a day earlier than the tremor counts, the third peaks four days earlier, and the fourth peaks five days earlier. Similar to sse 21, the slip finally stops near $46.8°N$. The slip positions of peaks 1 and 2 correspond to the zones 3 and 4 in the paper of Michel, et al. [10].

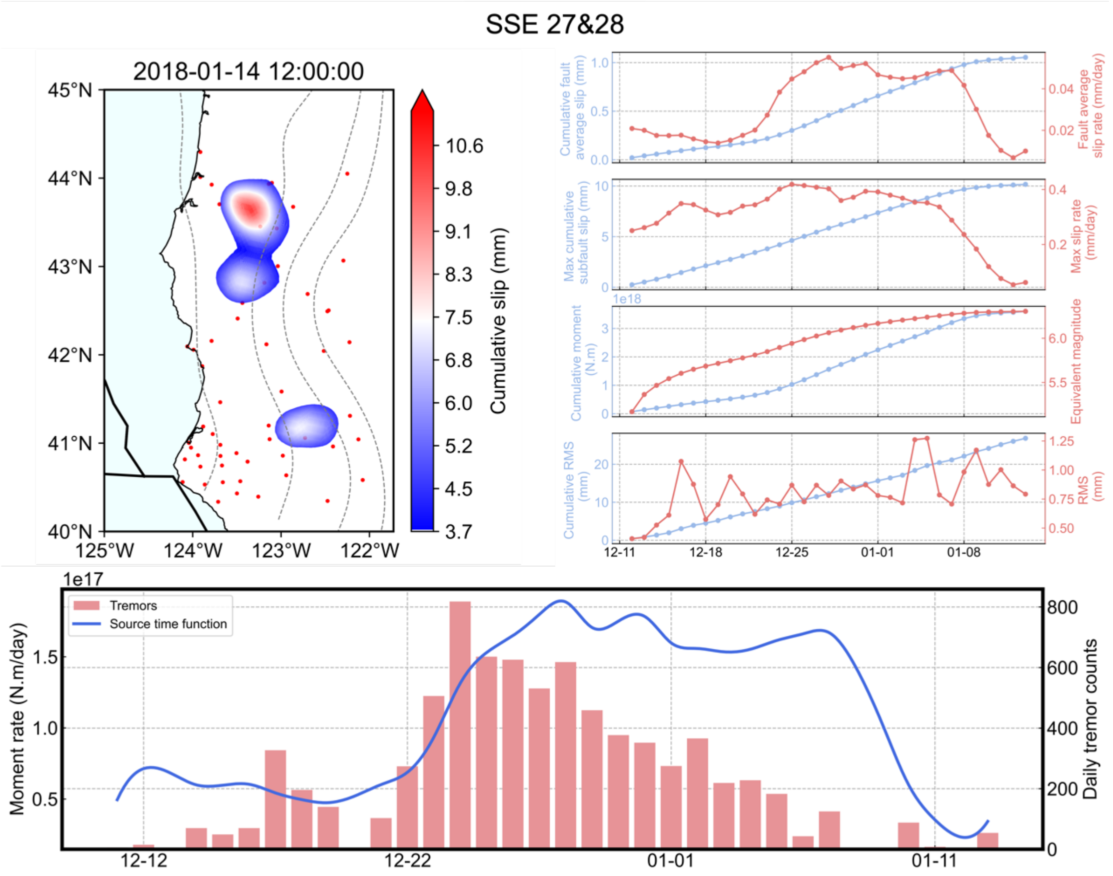

Figure S48: SSE 27&28. We select 79 stations used for inversion. Although the north and south slip patches can be distinguished, during this period, there is a high tremor count and dispersed distribution, making it difficult to differentiate. Therefore, we simultaneously invert these two events.

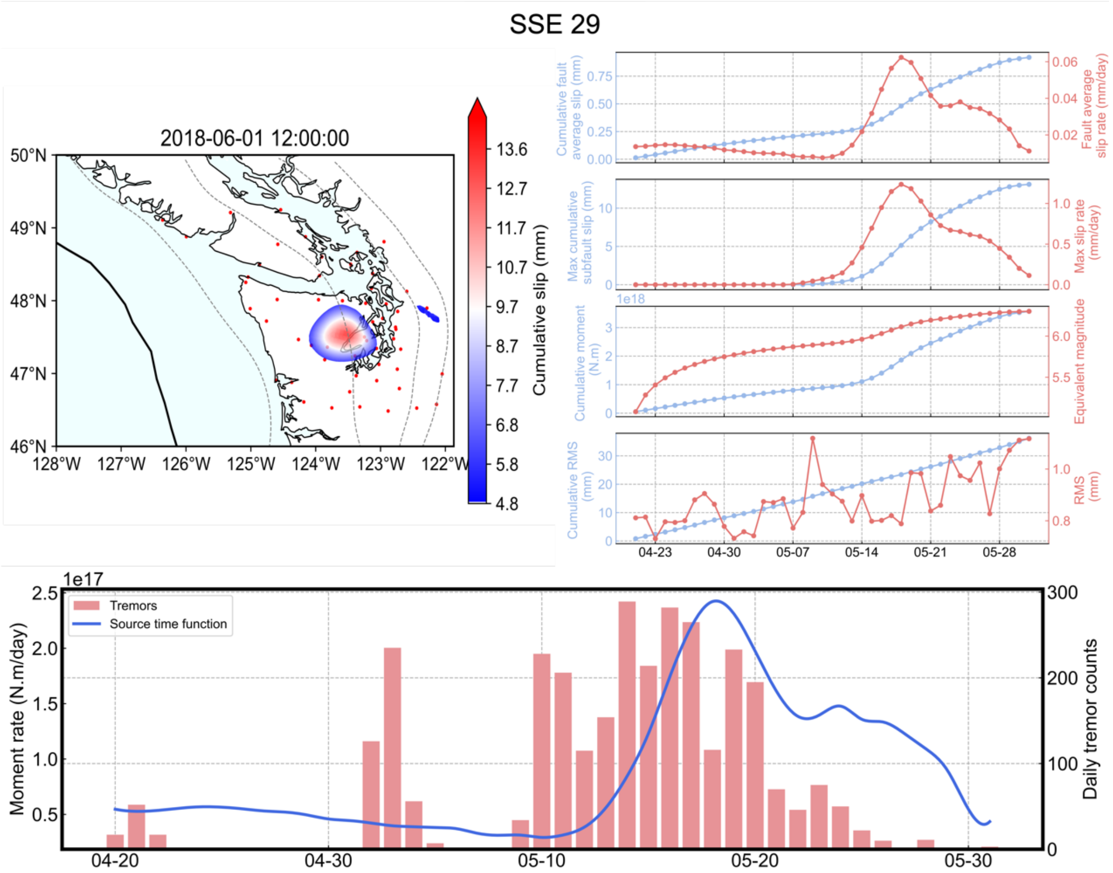

Figure S49: SSE 29. We select 59 stations used for inversion. The event has a main peak of moment rate that follows the peak in the number of tremors by four days.

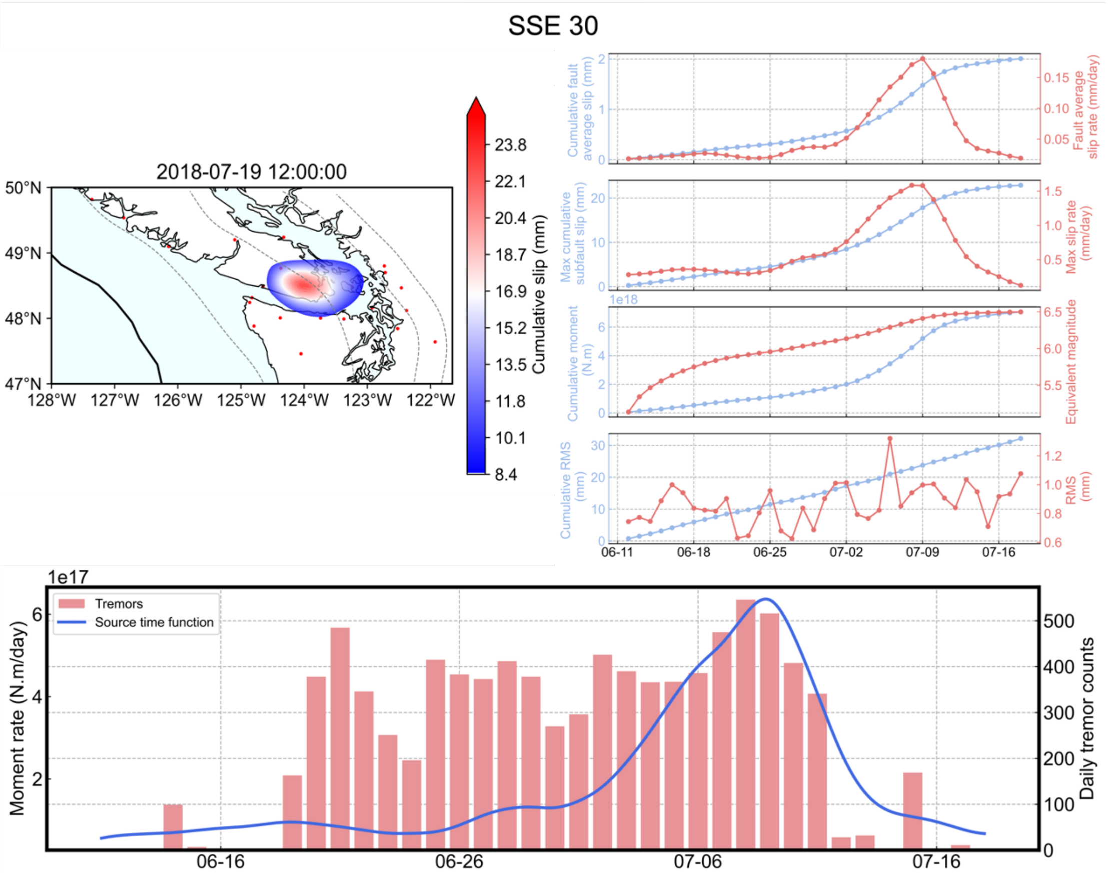

Figure S50: SSE 30. We select 26 stations used for inversion. The event has a main peak of moment rate. Tremor counts increase significantly and migrate along the trend before the moment rate reaches its peak, approximately 20 days prior. However, the slip zone remains unchanged.

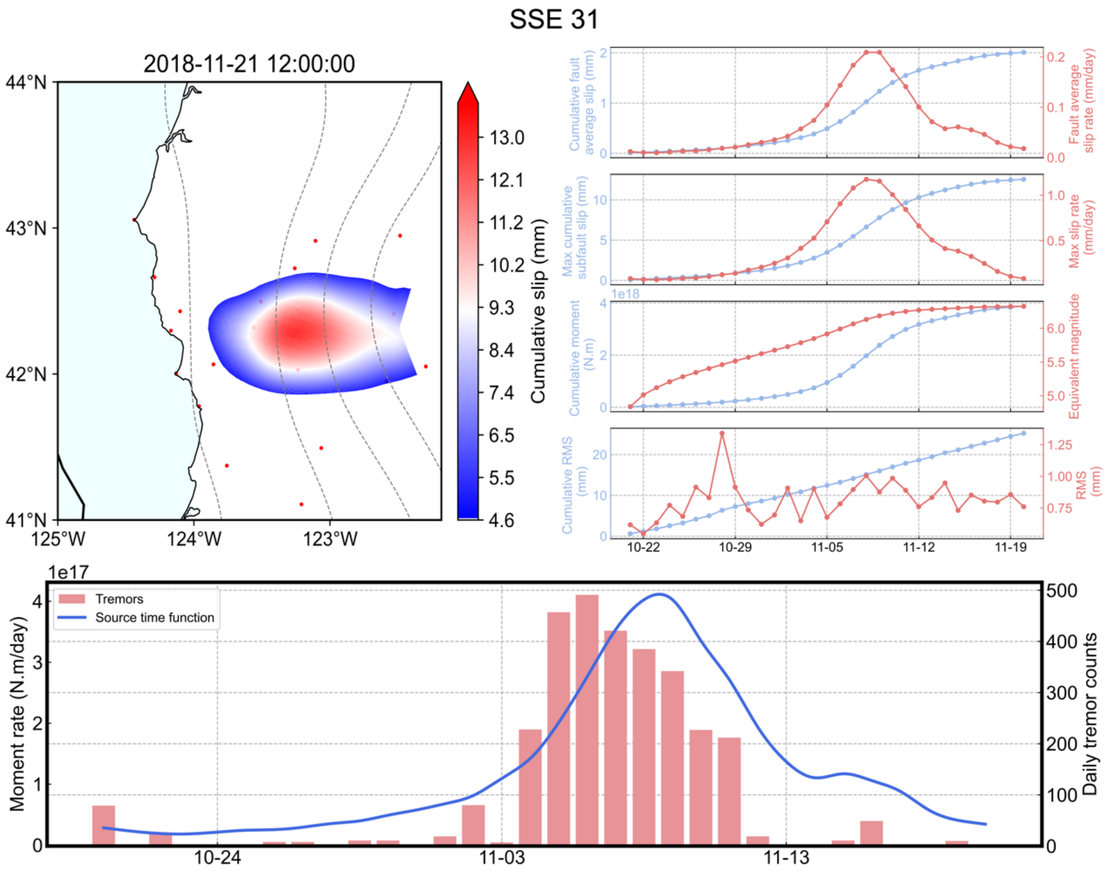

Figure S51: SSE 31. We select 27 stations used for inversion. The event has a main peak of moment rate that follows the peak in the number of tremors by four days.

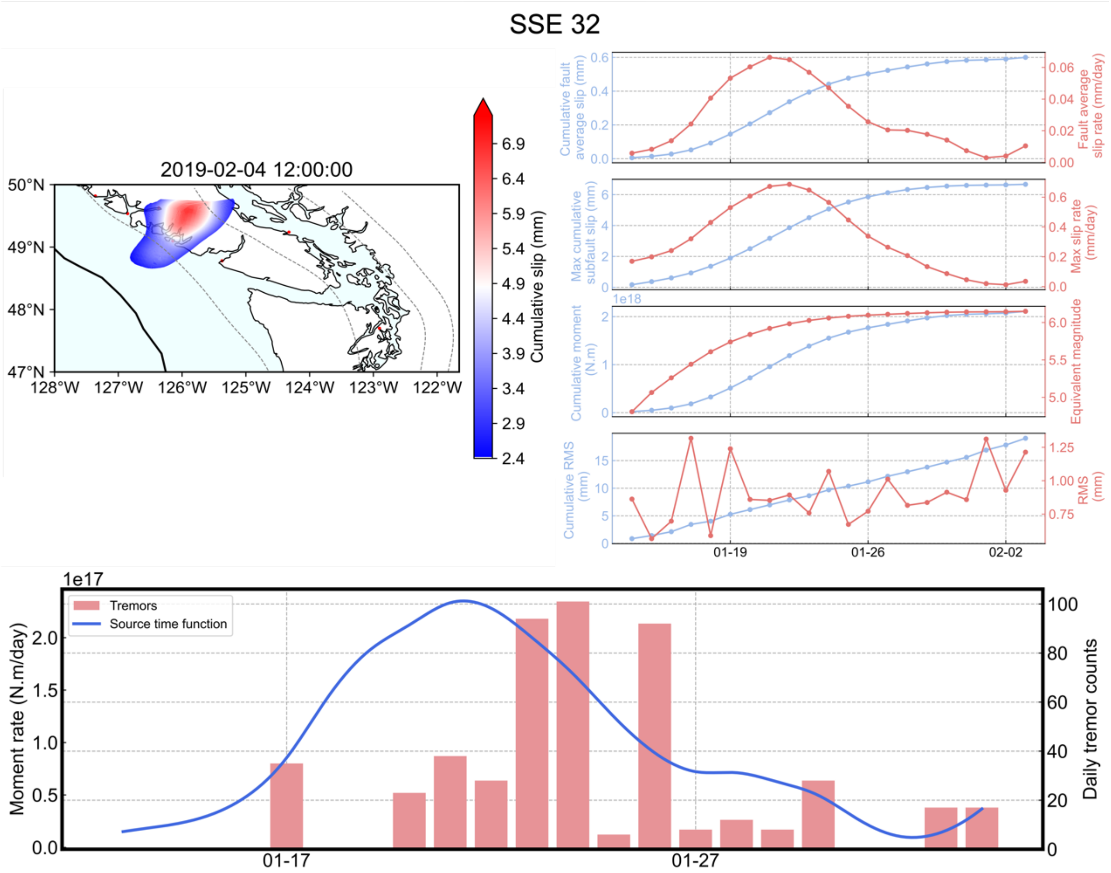

Figure S52: SSE 32. We select 9 stations used for inversion. The event has a main peak of moment rate with a continuous tremor burst. The moment is Mw 6.1. However, constrained by the scope of the Slab2.0 fault, our inversion results indicate slip is concentrated at the northern boundary of the fault. It suggests that there might be additional faults beyond the northern range of Slab2.0 that were not included, so actual magnitudes might be larger than our inversion results suggest.

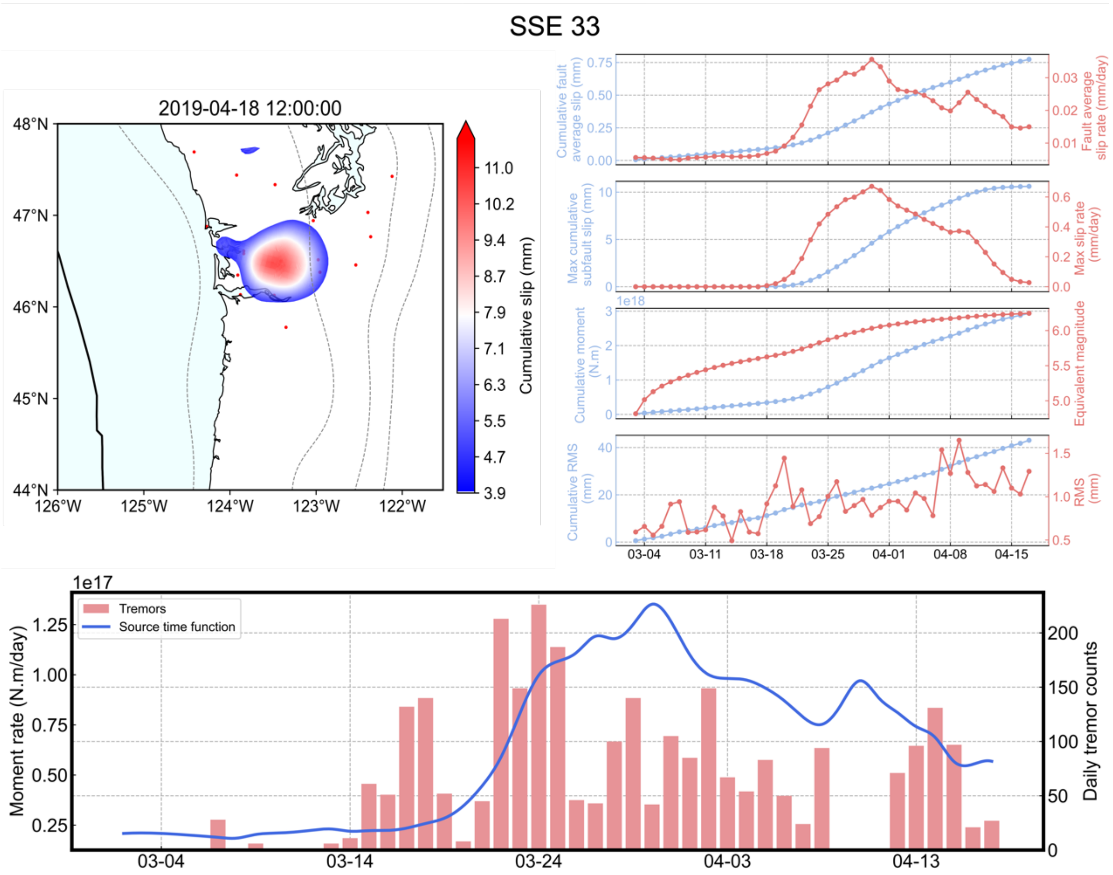

Figure S53: SSE 33. We select 22 stations used for inversion. This SSE has one main peak of moment rate followed by a smaller peak. The second smaller peak is due to a short-distance migration, occurring five days earlier than the peak in tremor counts.

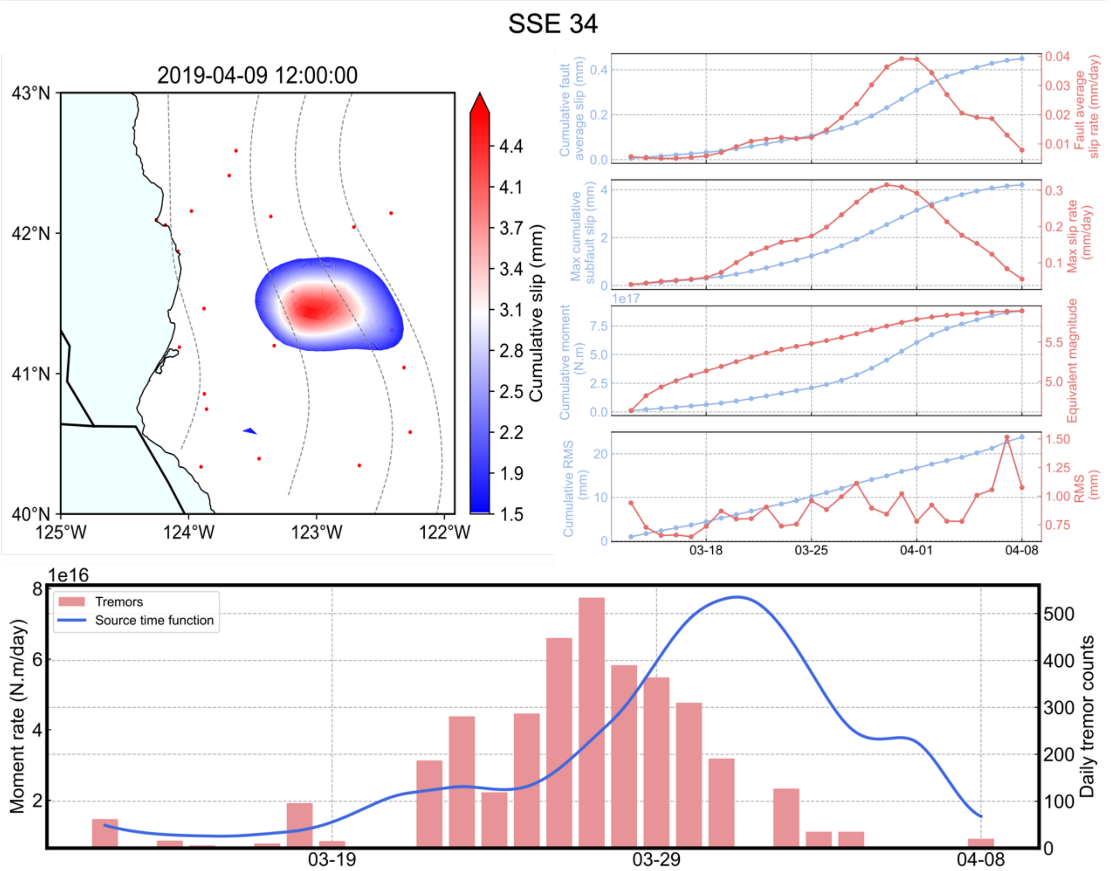

Figure S54: SSE 34. We select 32 stations used for inversion. The event has a main peak of moment rate that follows the peak in the number of tremors by five days.

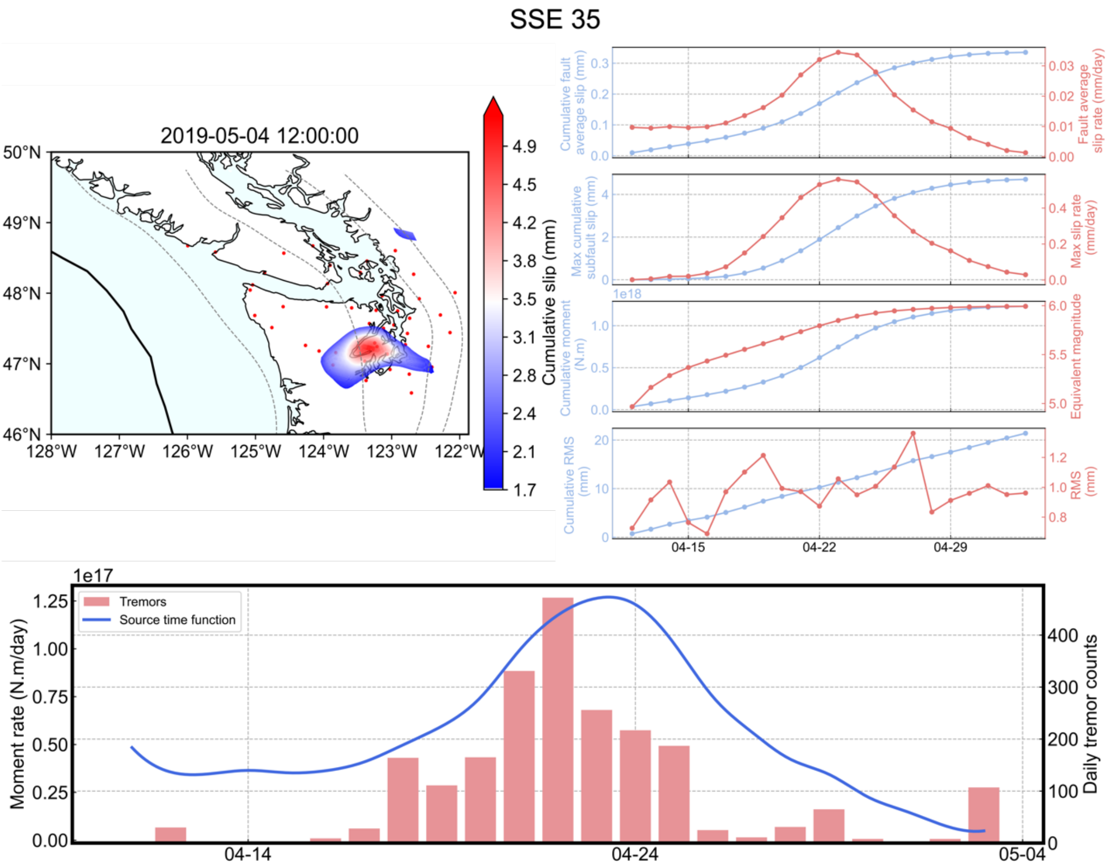

Figure S55: SSE 35. We select 59 stations used for inversion. The event has a main peak of moment rate that follows the peak in the number of tremors by three days.

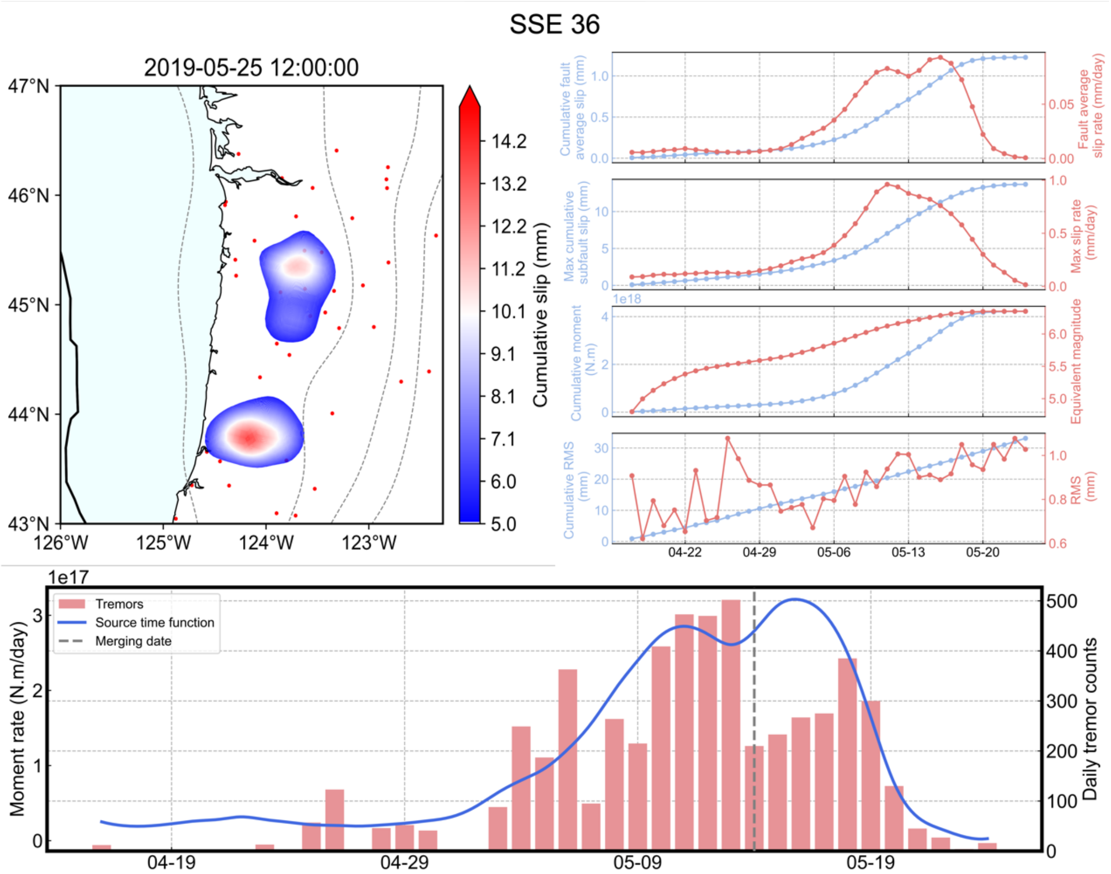

Figure S56: SSE 36. We select 66 stations used for inversion. After reaching its peak, the slip in the northern region migrates southward along the strike. On May 14th, the slip zone in the southern area, which is in a decaying phase, merges with the slip from the northern migration. Following the merger, the moment rate rises again and peaks two days later.

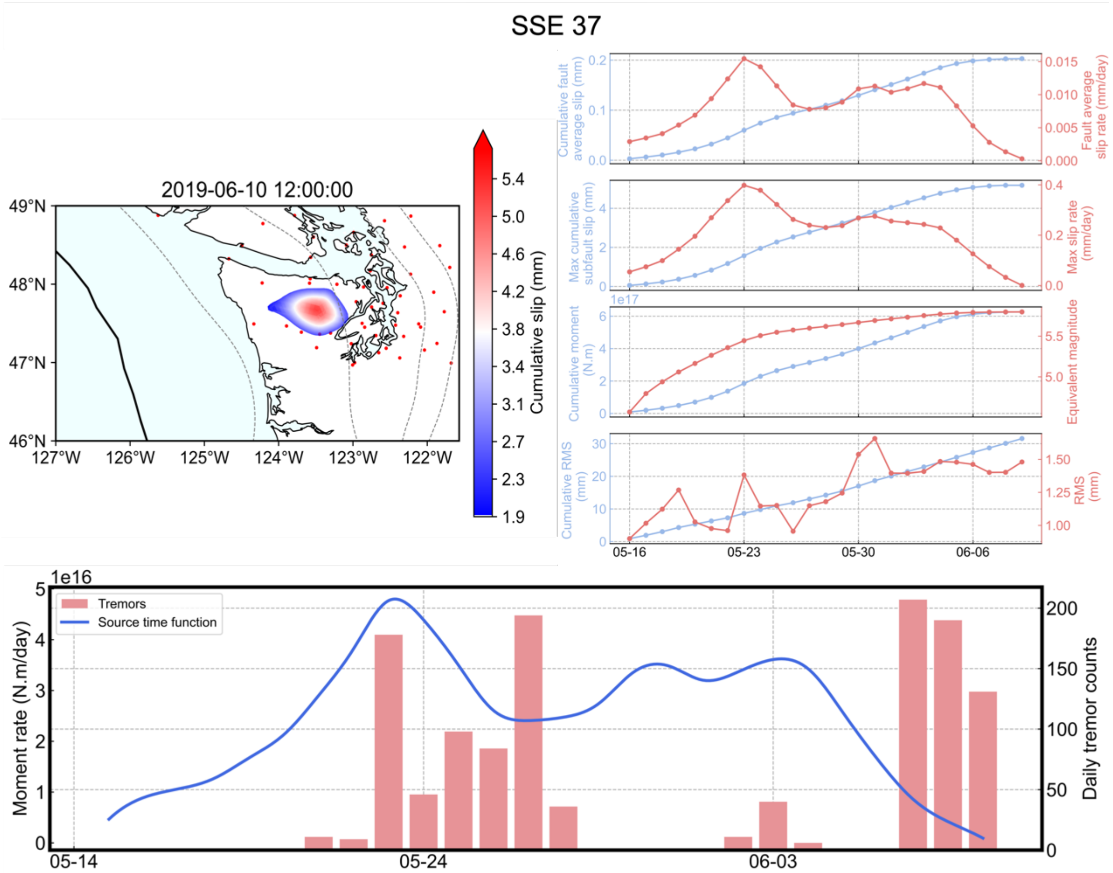

Figure S57: SSE 37. We select 63 stations used for inversion. The event is a small event and the magnitude is Mw 5.8.

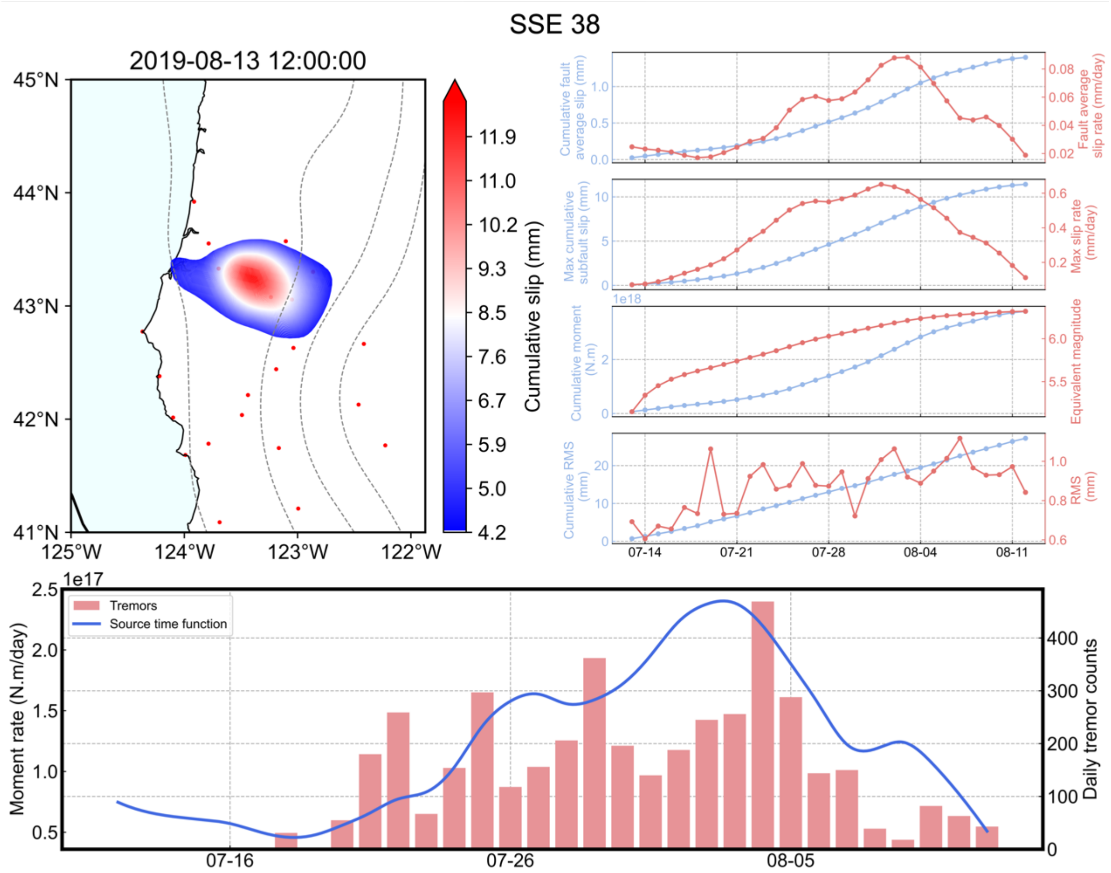

Figure S58: SSE 38. We select 30 stations used for inversion. This event has one main peak of moment rates that precedes the peak in the number of tremors by one day.

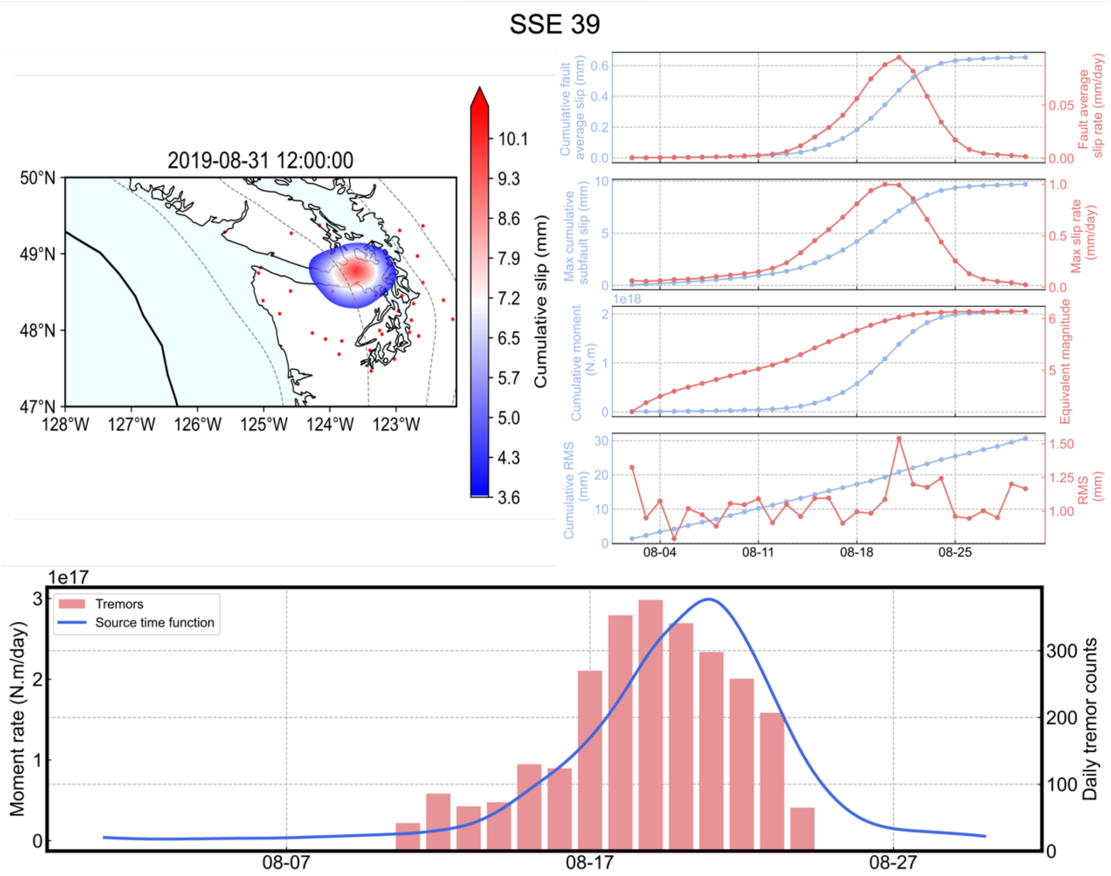

Figure S59: SSE 39. We select 51 stations used for inversion. The event has a main peak of moment rate that follows the peak in the number of tremors by two days.

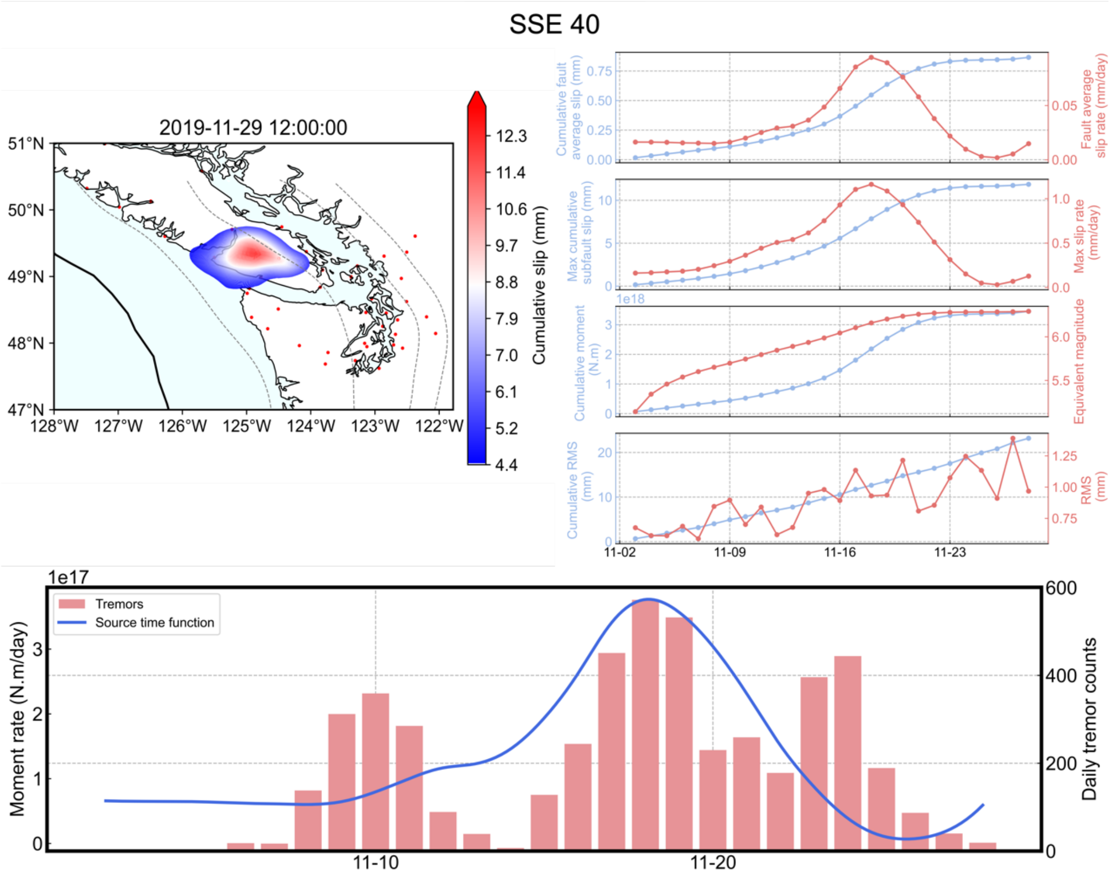

Figure S60: SSE 40. We select 57 stations used for inversion. This event has one main peak of moment rates and coincides with a continuous tremor burst.

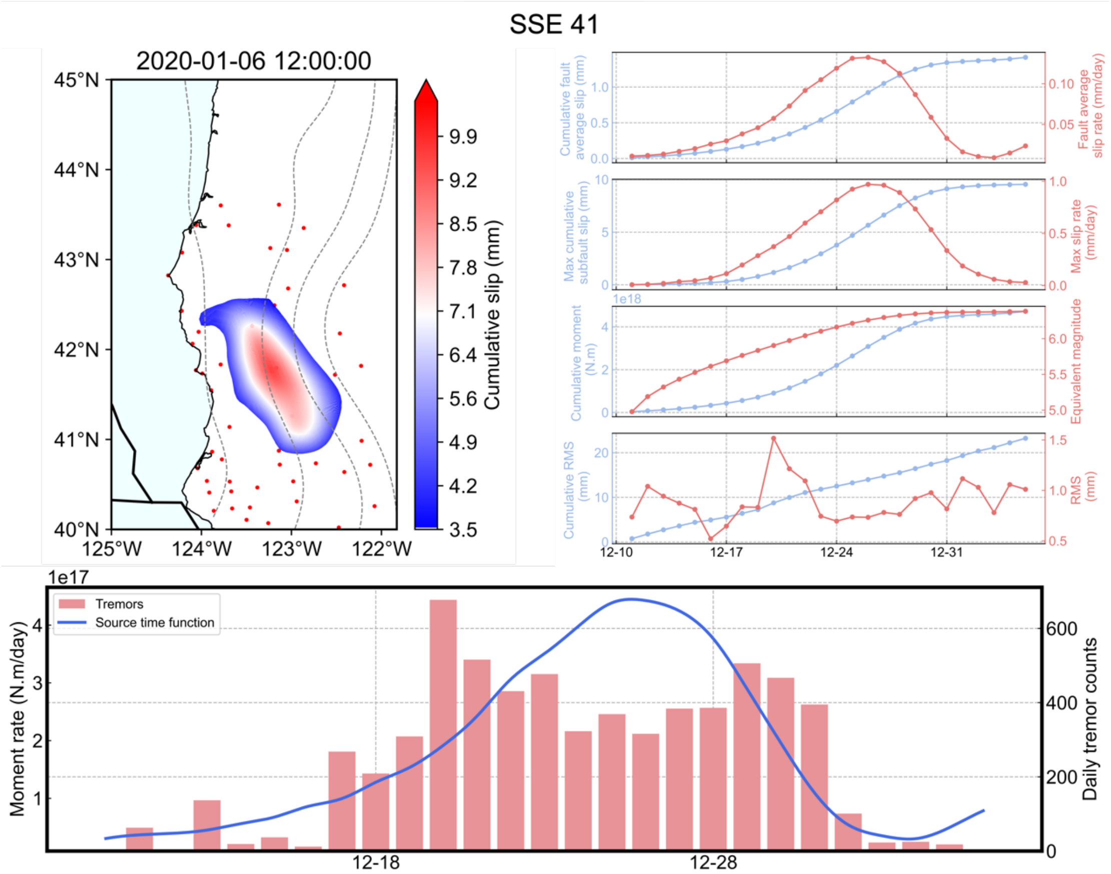

Figure S61: SSE 41. We select 57 stations used for inversion. This event has a main peak in moment rate occurring during tremor migration, with the peak located at the trough in the tremor count histogram.

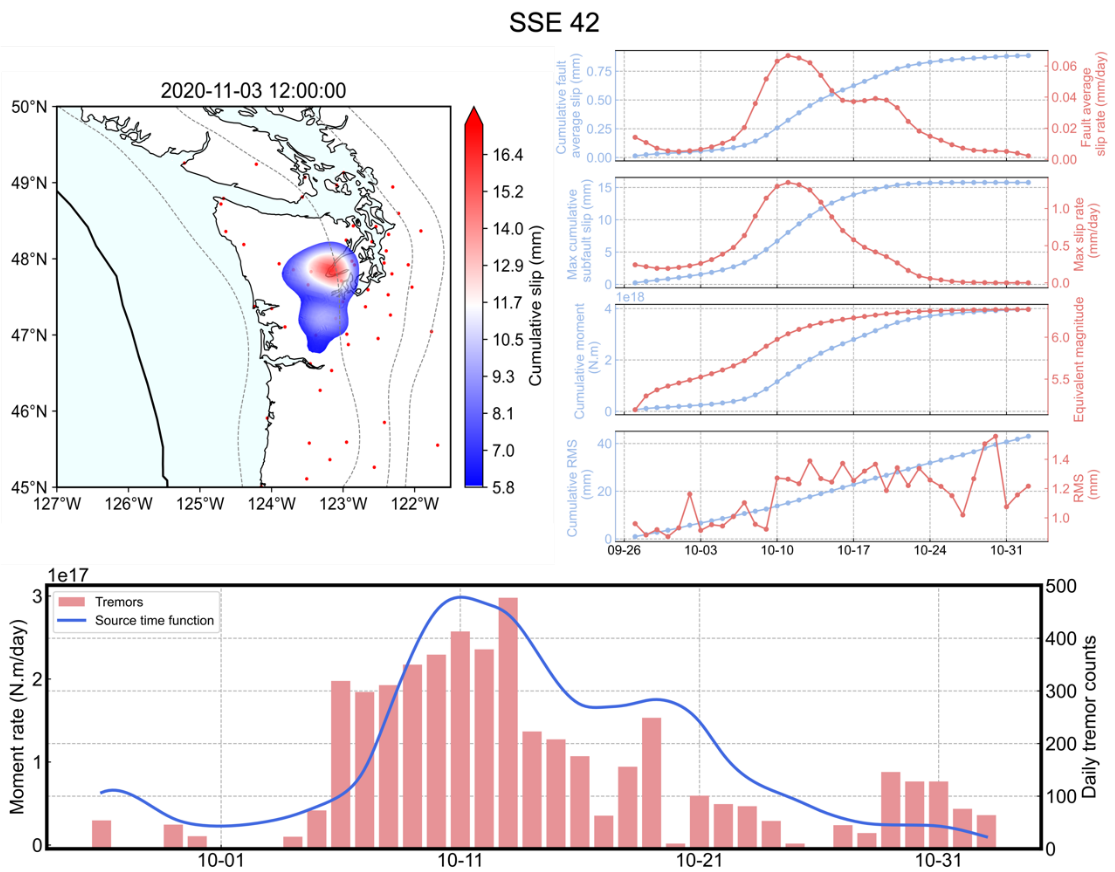

Figure S62: SSE 42. We select 70 stations used for inversion. This SSE is a continuous migration event. The SSE has one main peak of moment rate followed by a smaller peak. After reaching its peak, the slip migrates southward along the strike and reaches another minor peak. These two peak regions correspond to zones 2 and 3 in Michael's paper.

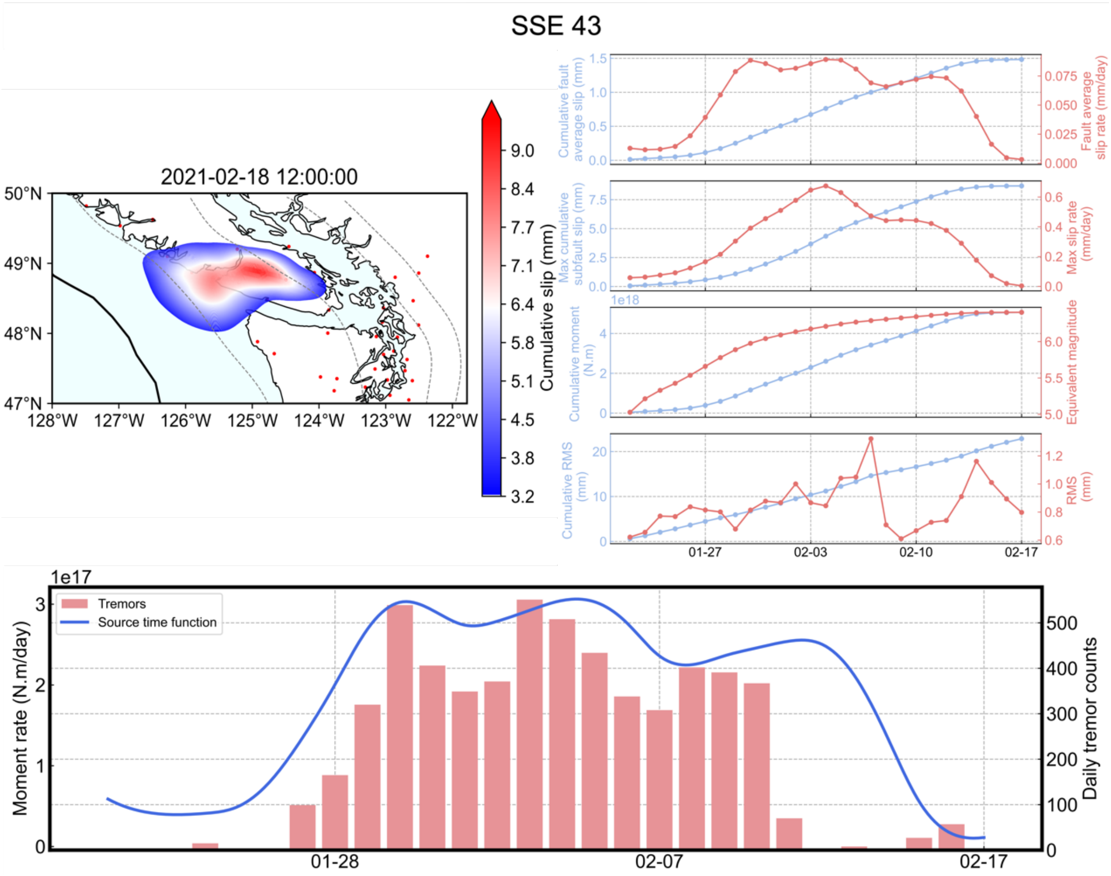

Figure S63: SSE 43. We select 44 stations used for inversion. This SSE is a continuous migration event. This SSE has 3 main peaks of moment rates. The position of peak 2 in this event aligns closely with peak 3 of SSE 23. While SSE 23 events migrate from south to north along the strike, this event migrates from north to south. Perhaps we can divide Zone 1 in the paper of Michel, et al. [10] into three parts: central Vancouver Island, southern Vancouver Island, and the Salish Sea.

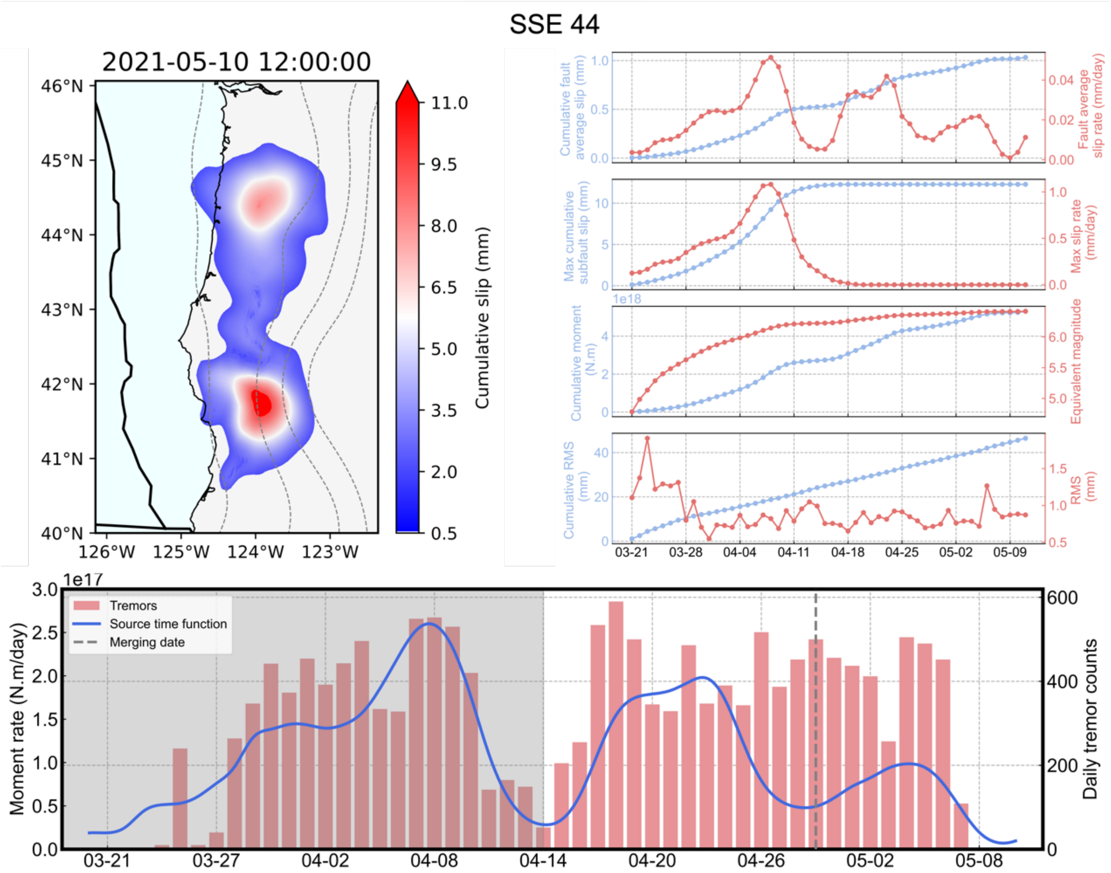

Figure S64: SSE 44. We select 40 stations used for inversion. SSE 44 experiences a merging event during the decaying phase of slip.

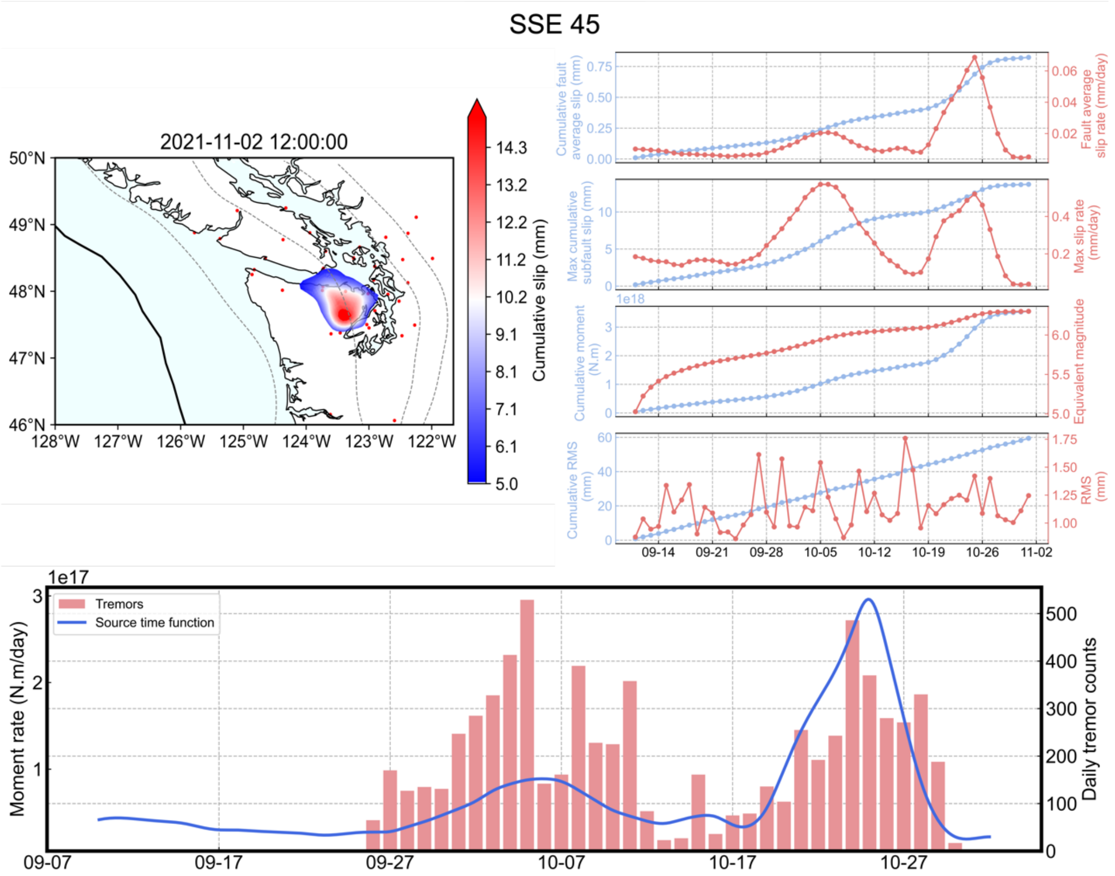

Figure S65: SSE 45. We select 44 stations used for inversion. This SSE has 2 peaks of moment rate release coincides with a continuous tremor burst.

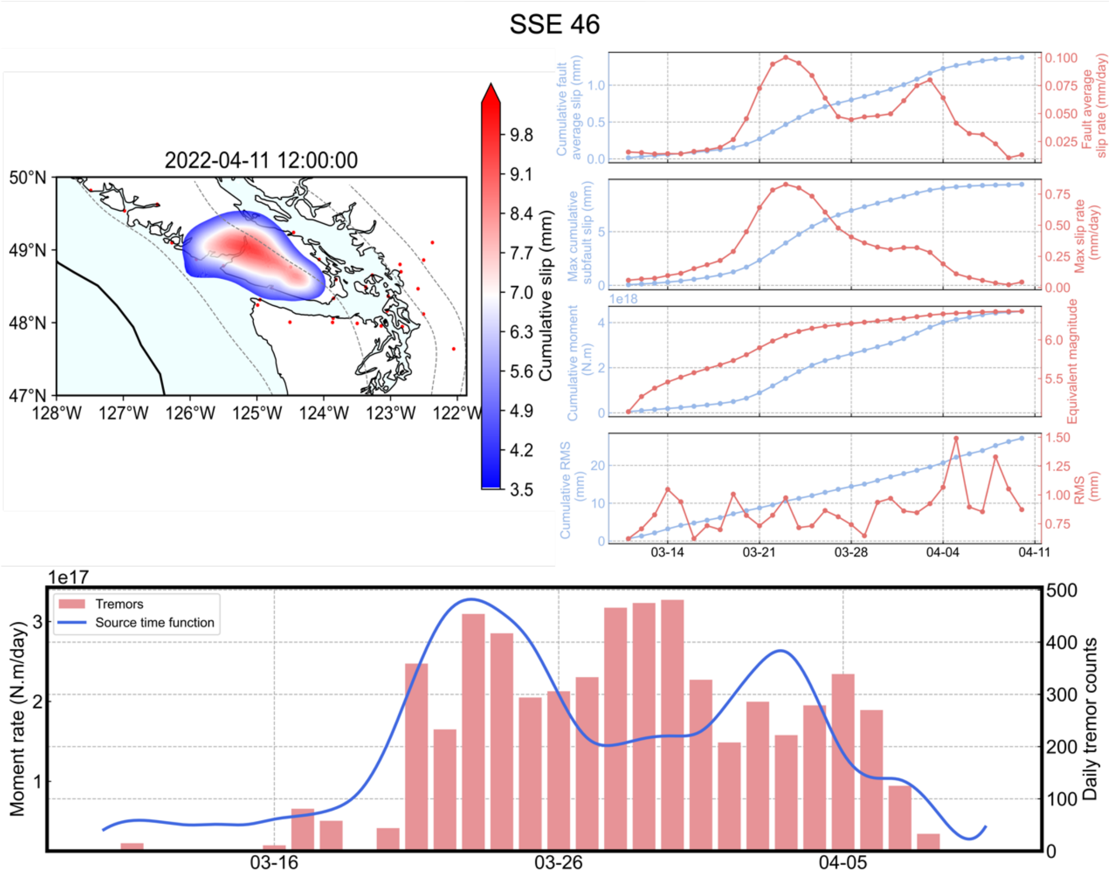

Figure S66: SSE 46. We select 33 stations used for inversion. This SSE is a continuous migration event. This SSE has 2 main peaks of moment rates. The slip position of the peak in this event aligns with slip position of the peak in SSE 43, supporting conclusion of SSE43.

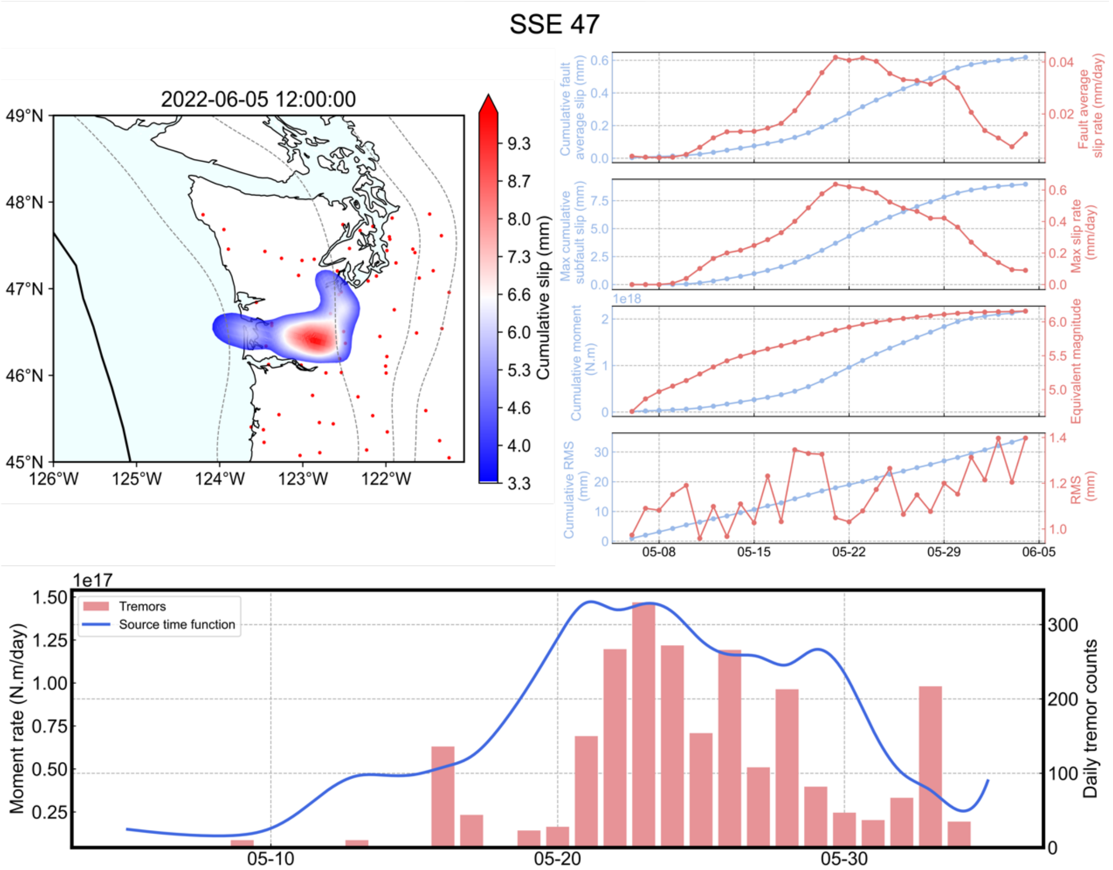

Figure S67: SSE 47. We select 86 stations used for inversion. The event has a main peak of moment rates.

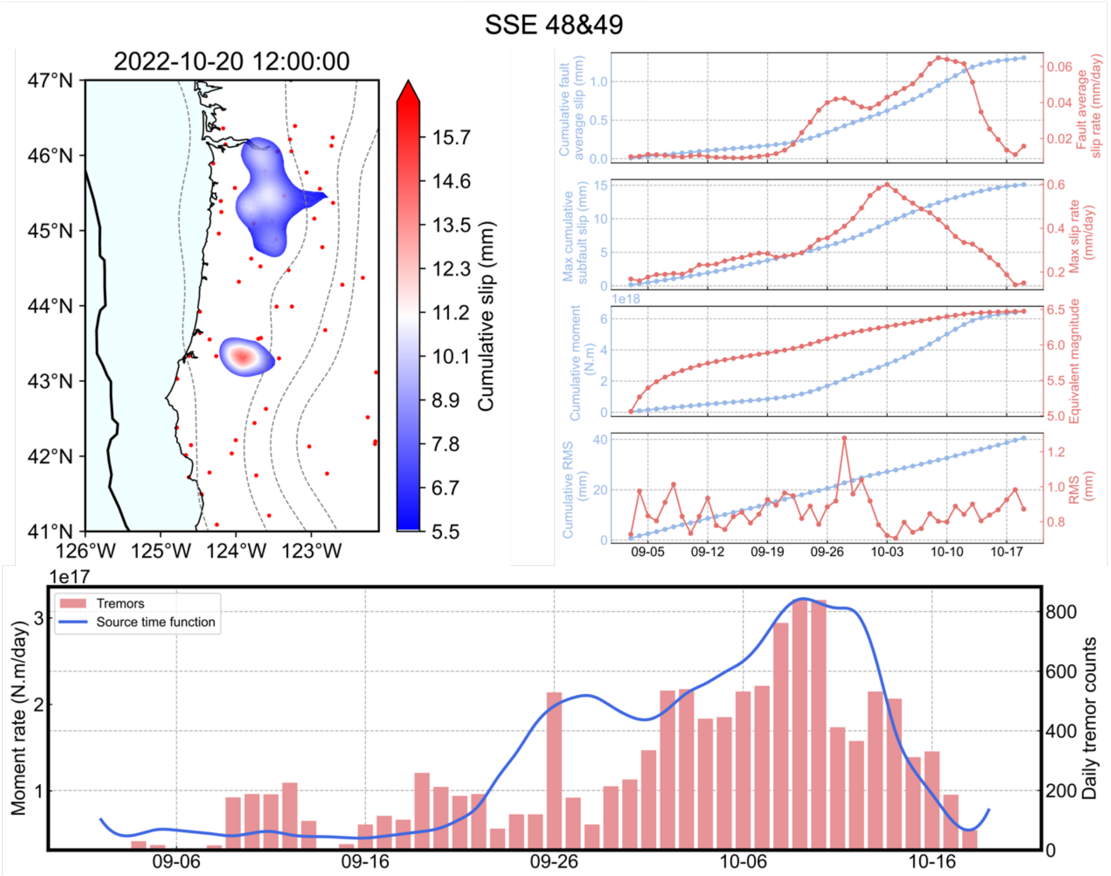

Figure S68: SSE 48&49. We select 80 stations used for inversion. This SSE exhibits two events, one is a typical small SSE in the south, while the other is a migrating SSE in the north, with a migration speed of 7.2 km/day along the strike. The slip finally stops near 45.0°N, corresponding to the boundary between zones 4 and 5 in the paper of Michel, et al. [10].

References

1. Wang, J., *et al.* Detecting slow slip events in the Cascadia subduction zone from GNSS time series using deep learning. *GPS Solutions* **28**, 1-16 (2024).
2. Gualandi, A., Serpelloni, E. & Belardinelli, M.E. Blind source separation problem in GPS time series. *Journal of Geodesy* **90**, 323-341 (2016).
3. Michel, S., Gualandi, A. & Avouac, J.-P. Interseismic coupling and slow slip events on the Cascadia megathrust. *Pure and Applied Geophysics* **176**, 3867-3891 (2019).
4. Vinutha, H.P., Poornima, B. & Sagar, B.M. Detection of outliers using interquartile range technique from intrusion dataset. in *Information and Decision Sciences: Proceedings of the 6th International Conference on FICTA* 511-518 (2018).
5. Schneider, T. Analysis of incomplete climate data: Estimation of mean values and covariance matrices and imputation of missing values. *Journal of climate* **14**, 853-871 (2001).
6. Wiemer, S. & Wyss, M. Minimum magnitude of completeness in earthquake catalogs: Examples from Alaska, the western United States, and Japan. *Bulletin of the Seismological Society of America* **90**, 859-869 (2000).
7. Hayes, G.P., *et al.* Slab2, a comprehensive subduction zone geometry model. *Science* **362**, 58-61 (2018).
8. Molina-Ormazabal, D., Ampuero, J.-P. & Tassara, A. Diverse slip behaviour of velocity-weakening fault barriers. *Nature Geoscience* **16**, 1200-1207 (2023).
9. Bletery, Q. & Nocquet, J.-M. Slip bursts during coalescence of slow slip events in Cascadia. *Nature communications* **11**, 2159 (2020).
10. Michel, S., Gualandi, A. & Avouac, J.-P. Similar scaling laws for earthquakes and Cascadia slow-slip events. *Nature* **574**, 522-526 (2019).
