# Supplementary material for: Secondary acceleration of slip fronts driven by slow slip event coalescence in subduction zones
Source: Nat Commun. 2025 Oct 29;16:9561. doi: 10.1038/s41467-025-64616-3 (PMC12572340; doi:10.1038/s41467-025-64616-3)
Supplement: Supplementary file 2 — Description of Additional Supplementary Files [file 41467_2025_64616_MOESM2_ESM.pdf]

Description of Additional Supplementary Files

File Name: Supplementary Movie 1
Description: The 11-year slip evolution of SSEs in Cascadia
